# Daeshiho-tang attenuates inflammatory response and oxidative stress in LPS-stimulated macrophages by regulating TLR4/MyD88, NF-κB, MAPK, and Nrf2/HO-1 pathways

**DOI:** 10.1038/s41598-023-46033-y

**Published:** 2023-11-02

**Authors:** Yong Jin Oh, Seong Eun Jin, Hyeun-Kyoo Shin, Hyekyung Ha

**Affiliations:** KM Science Research Division, Korea Institute of Oriental Medicine, 1672 Yuseong-daero, Yuseong-Gu, Daejeon, 34054 Korea

**Keywords:** Immunology, Molecular biology

## Abstract

Daeshiho-tang (DSHT), a traditional herbal formula with diverse pharmacological effects, has shown promise in medicine owing to its anti-hypertensive, anti-diabetic, and anti-inflammatory properties. However, the precise molecular mechanism underlying these effects remains unclear. Thus, we investigated the effect of DSHT on inflammatory response and oxidative stress to understand its molecular mechanism using lipopolysaccharide (LPS)-induced macrophage (RAW 264.7) cells. DSHT decreased the contents of nitric oxide (NO) and prostaglandin E_2_ (PGE_2_) through downregulating inducible nitric oxide synthase (iNOS) and cyclooxygenase-2 (COX-2) protein expressions. DSHT suppressed the LPS-induced TLR4 as well as MyD88, subsequently suppressing the NF-κB activation and the phosphorylation of MAPK (p38, ERK, and JNK). Radical scavenging activity results revealed a dose-dependent response of DSHT with diminished ABTS activity, a hallmark of oxidative stress potential. Furthermore, DSHT enhanced Nrf2 and HO-1 expression in response to LPS. Collectively, our findings indicated that DSHT exert anti-inflammatory effect and regulating oxidative stress by modulating TLR4/MyD88, NF-κB, MAPK, and Nrf2/HO-1 pathways, consequently can provide potential therapeutic strategy for the prevention and treatment of inflammation and oxidative stress-related diseases.

## Introduction

Inflammation is an adaptive reaction that regulates numerous pathophysiological conditions against noxious stimuli and conditions, including viral or bacterial infection and cellular damage^1–3^. A precisely modulated inflammatory response protect the body from damage; however inappropriate and excessive inflammation can promote a spectrum of immune system disorders, including allergy, atherosclerosis, ischemic heart disease, and tumor progression^4–6^.

Lipopolysaccharides (LPS), commonly known as endotoxin, act as inflammatory agents, promoting the activation of macrophages to generate inflammatory mediators and cytokines^7,8^.

In particular, the toll-like receptor (TLR4) functions as a first responder, becoming activated on the surface of various immune cells, including macrophages, dendritic cells, and B cells. Upon binding to LPS, TLR4 triggers the recruitment of adaptor molecules, namely myeloid differentiation factor 88 (MyD88) and toll/interleukin-1 receptor domain-containing adapter inducing interferon-β, which subsequently trigger downstream signaling pathways such as nuclear factor kappa-light-chain-enhancer of activated B (NF-κB) and mitogen-activated protein kinase (MAPK)^9–11^. The activation of these signaling transductions plays a pivotal role in the secretion of interleukin (IL) 1 beta (IL-1β), IL-6, tumor necrosis factor alpha (TNF-α), nitric oxide (NO) and prostaglandin E_2_ (PGE_2_)^12–14^. The excessive accumulation of these inflammatory factors induces tissue and cellular damage, thereby exacerbating the inflammatory disease^15–17^.

Oxidative stress lead to elevated levels of reactive oxygen species (ROS), which impact numerous biological processes, including inflammation^4,18^. Upon LPS stimulation, macrophages trigger inflammatory cascades that in turn induced oxidative stress, consequently, contributing to the development of inflammation and abnormal inflammatory cytokine production^4,19^. Further, increased ROS level in LPS-stimulated macrophages not only sustain inflammation but also drive excessive production of inflammatory cytokines^4,19^.

*Daeshiho-tang* (DSHT), known as *Dachaihu-tang* in China; *Daisaiko-to* in Japan, has been used throughout East Asia, particularly Korea, to alleviate symptoms such as fatigue, tenesmus, abdominal pain, nocturnal emission, and dry throat^20^. DSHT consists of seven herbal medicine, Bupleuri Radix (*Bupleurum falcatum* L.), Scutellariae Radix (*Scutellaria baicalensis* Georgi), Paeoniae Radix (*Paeonia lactiflora* Pall.), Rhei Radix et Rhizoma (*Rheum palmatum* L.), Ponciri Fructus Immaturus (*Poncirus trifoliata* (L.) Raf.), Pinelliae Tuber (*Pinellia ternate* (Thunb.) Makino). Six ingredients from DSHT such as saikosaponin (Bupleuri Radix), baicalin and wogonin (Scutellariae radix), sennoside (Rhei Rhizoma), albiflorin and paeoniflorin (Paeoniae Radix), and naringin (Ponciri Fructus Immaturus) have demonstrated anti-inflammatory properties^21–25^. Several studies have revealed that DSHT possesses numerous medicinal properties, including anti-hypertensive^26^, anti-diabetic^27^, and anti-hepatotoxic effect^28^. Moreover, Liu et al. recently demonstrated that DSHT regulated the inflammatory response^29^. Despite its well-documented pharmacological effects, the molecular mechanism of DSHT’s anti-inflammatory activity and oxidative stress remain unexplored. Therefore, this study aims to scrutinize the effect of DSHT on inflammatory response and oxidative stress in LPS-stimulated macrophages to identify its underlying mechanism of action.

## Results

### DSHT induces cell proliferation against macrophages

**Figure 1:**
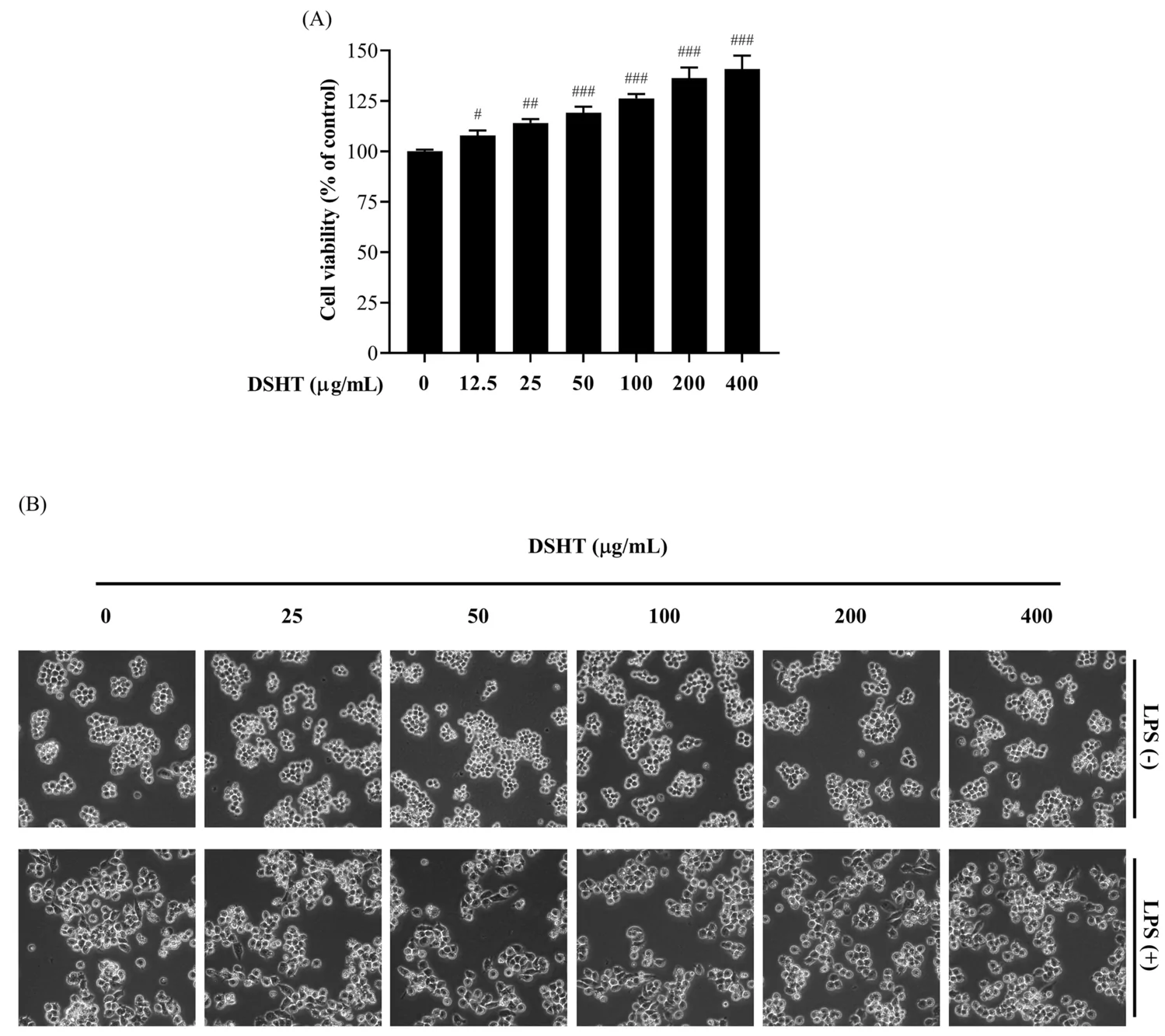
Effect of DSHT on the cell proliferation and morphology of RAW 264.7 macrophages. (**A**) Cells were treated with the indicated concentration of DSHT (12.5–400 μg/mL) for 24 h. Cell proliferation was determined using the colorimetric Cell counting Kit-8 (CCK-8) assay. (**B**) Cell morphology was visualized under an inverted-phase contrast microscope (× 200). Data are presented as mean ± standard deviation (SD) (n = 4). ^#^*p* < 0.05, ^##^*p* < 0.01, and ^###^*p* < 0.001 vs. control.

We first investigated the impact of DSHT on cell proliferation using RAW 264.7 cells. As illustrated in Fig. 1A, DSHT promoted a concentration-dependent elevation in cell proliferation, with a notable increase of 107.8–140.8%. To evaluate the effects of DSHT on morphology, macrophages were exposed to varying concentrations of DSHT and/or LPS (Fig. 1B). In the absence of LPS, DSHT-treated macrophages exhibited no marked morphological alterations, maintaining their round shape similar to that in the normal state. Conversely, LPS-treated macrophages underwent a morphological transition, adopting a polygonal spindle-shaped pseudopodia, a hallmark of macrophage activation, and treatment with DSHT had no effect on cell morphology.

### DSHT inhibits LPS-triggered inflammatory mediators through the regulation of iNOS and COX-2

**Figure 2:**
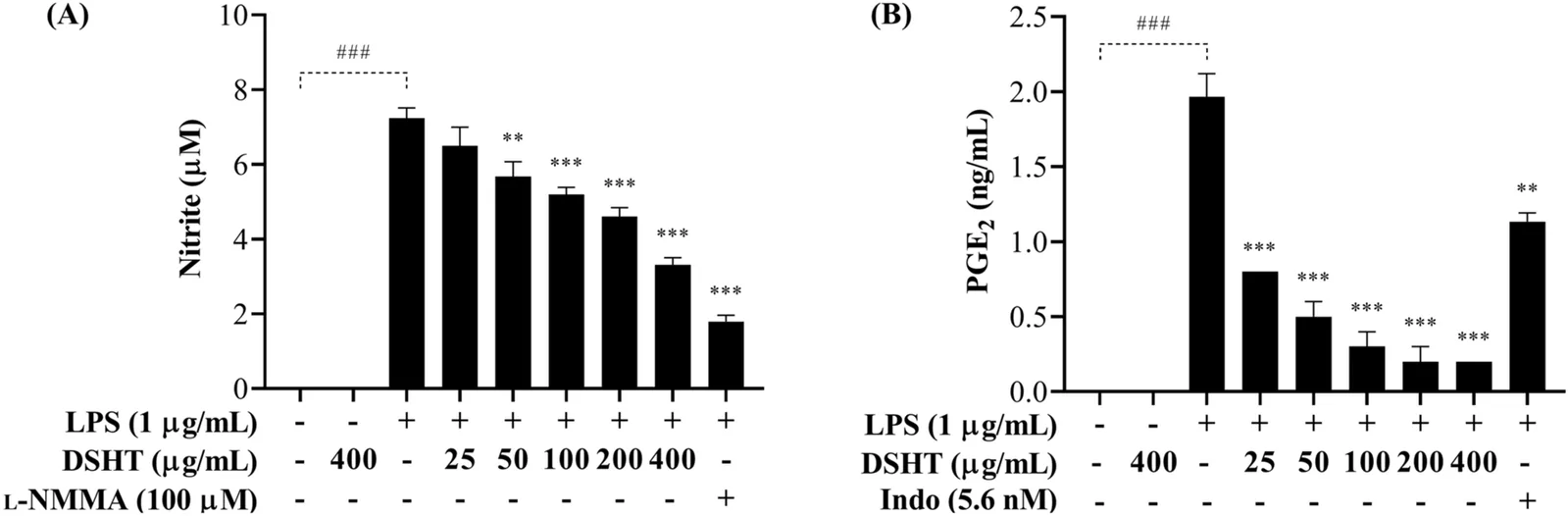
Effect of DSHT on the release of NO and PGE_2_ in LPS-stimulated RAW 264.7 macrophages. Content of NO (**A**) and PGE_2_ (**B**) on the RAW 264.7 cells treated with the indicated concentration of DSHT (25–400 μg/mL), L-NMMA (100 μM), and indomethacin (5.6 nM) and stimulated with or without LPS (1 μg/mL) for 24 h. Data are presented as mean ± SD (n = 3). ^###^*p* < 0.001 vs. untreated control; **p < 0.01 and ***p < 0.001 vs LPS.

**Figure 3:**
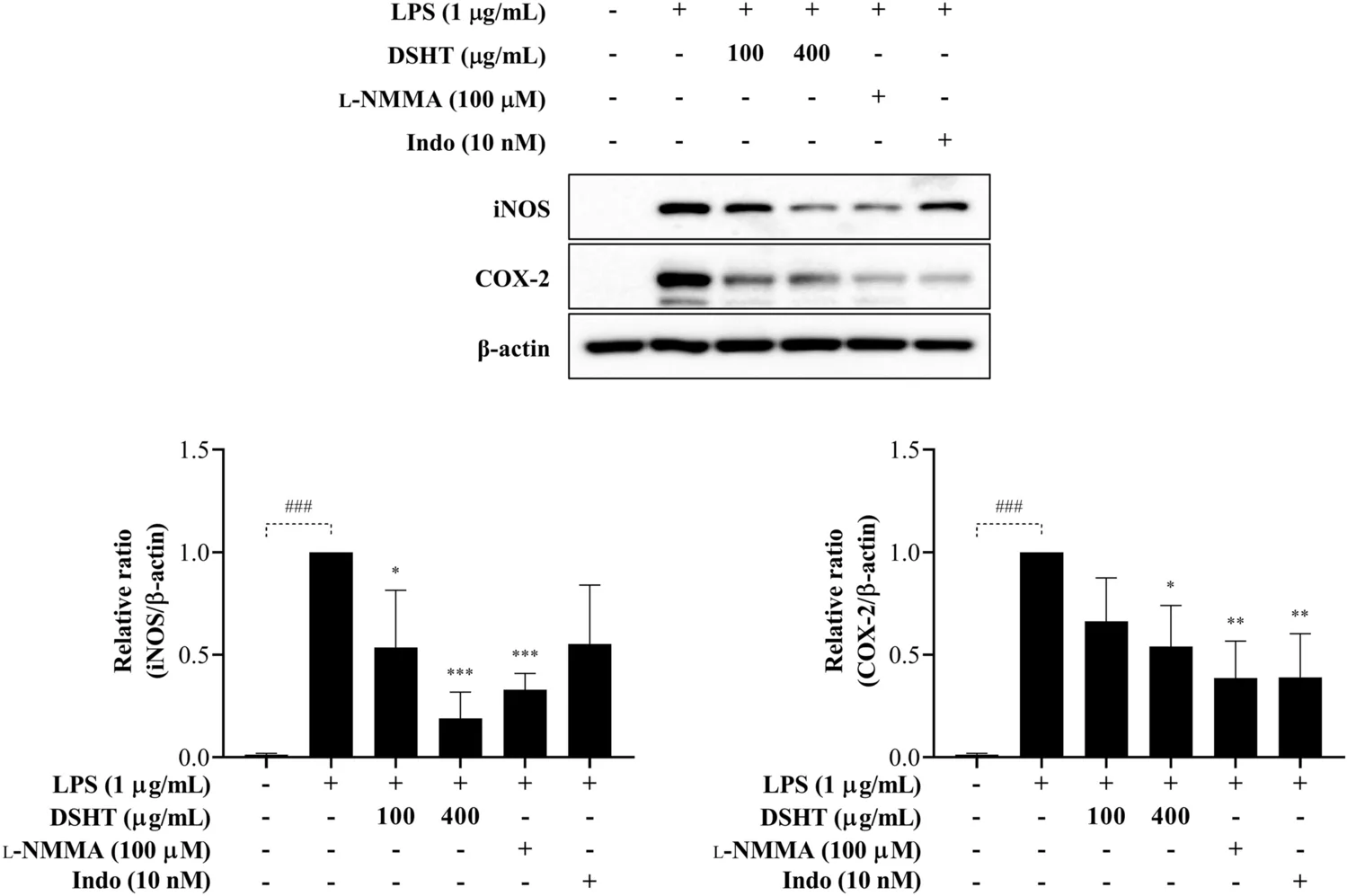
Effect of DSHT on iNOS and COX-2 expression in LPS-stimulated RAW 264.7 macrophages. Cells were treated with the indicated concentration of DSHT (100 and 400 μg/mL), L-NMMA (100 μM), and indomethacin (Indo; 10 nM) and stimulated with LPS (1 μg/mL) for 18 h. L-NMMA and indomethacin were employed as positive controls for the iNOS and COX-2 inhibitors, respectively. Data are presented as mean ± SD (n = 3). ^###^*p* < 0.001 vs. control; *p < 0.05, **p < 0.01, and ***p < 0.001 vs. LPS.

The generation of inflammatory mediators NO and PGE_2_ is governed by inducible nitric oxide synthase (iNOS) and cyclooxygenase-2 (COX-2) regulation in response to inflammation. Therefore, we used LPS-activated macrophages to stimulate an inflammatory environment and cause the release of NO and PGE_2_, to evaluate DSHT activity. As depicted in Fig. 2A and B, the secretions of NO and PGE_2_ were significantly elevated upon LPS stimulation; however, DSHT treatment alone had no significant effect on these mediators. DSHT concentration-dependently decreased the content of these LPS-stimulated mediators with an inhibition rate of 10.2–54.2% for NO and 62.4–92.2% for PGE_2_, respectively. Consistently, the positive control, L-NMMA and indomethacin also decreased the levels of NO and PGE_2_, respectively. Subsequently, we carried out western blotting to determine that the iNOS and COX-2 expression were involved to the effect of DSHT on the regulation of NO and PGE_2_. Akin to Fig. 2, LPS-treated macrophage upregulated iNOS and COX-2, while DSHT dose-dependently suppressed their expression (Fig. 3). Furthermore, at a high dose of 400 μg/mL, DSHT reduced the iNOS and COX-2 expression by 81% (*p* < 0.001) and 46.1% (*p* < 0.05), respectively.

### DSHT inhibits LPS-induced NF-κB pathway by regulating its translocation

**Figure 4:**
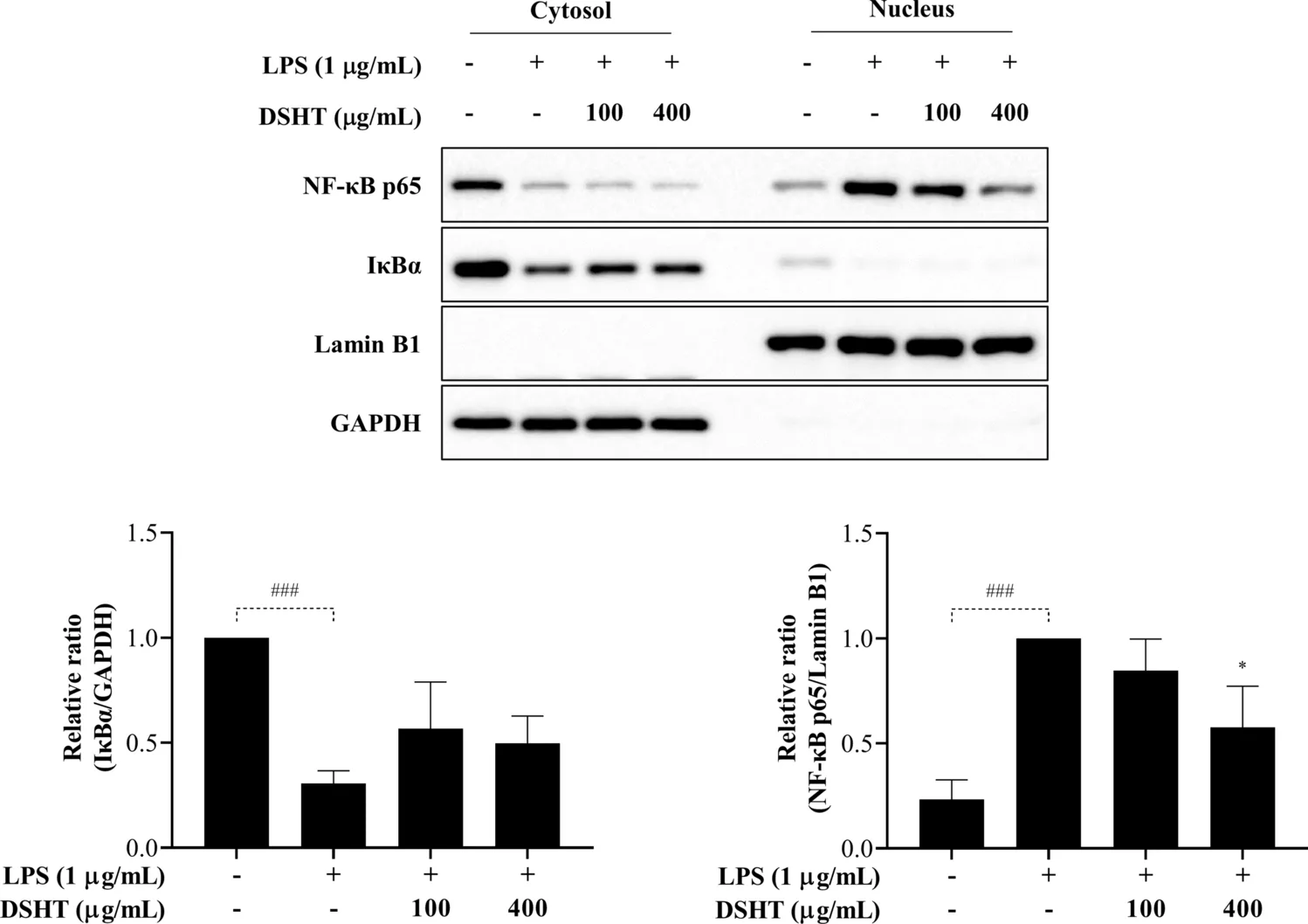
Inactivation of NF-κB pathways by DSHT in LPS-stimulated RAW 264.7 macrophages. Cells were treated with the indicated concentration of DSHT (100 and 400 μg/mL) and stimulated with LPS (1 μg/mL) for 30 min. Effect of DSHT on the expression of NF-κB and IκBα. GAPDH and Lamin B1 were employed as cytosol and nucleus loading control, respectively. Data are presented as mean ± SD (n = 3). ^###^*p* < 0.001 vs. control; *p < 0.05 vs. LPS.

To clarify the correlation between DSHT’s anti-inflammatory activity and inflammatory signaling pathways, we assessed DSHT activity on IκBα and NF-κB and its underlying mechanism. As shown in Fig. 4, LPS promotes the nuclear accumulation of NF-κB concomitant with decreased cytosolic IκBα and NF-κB, which implies NF-κB activation. In terms of translocated NF-κB, DSHT exhibited dose-dependent reduction of 15.2% and 42.1% (*p* < 0.05) at 100 and 400 μg/mL, respectively, with a significant effect observed at 400 μg/mL.

### DSHT regulated LPS-induced MAPK pathway by inhibiting its phosphorylation

**Figure 5:**
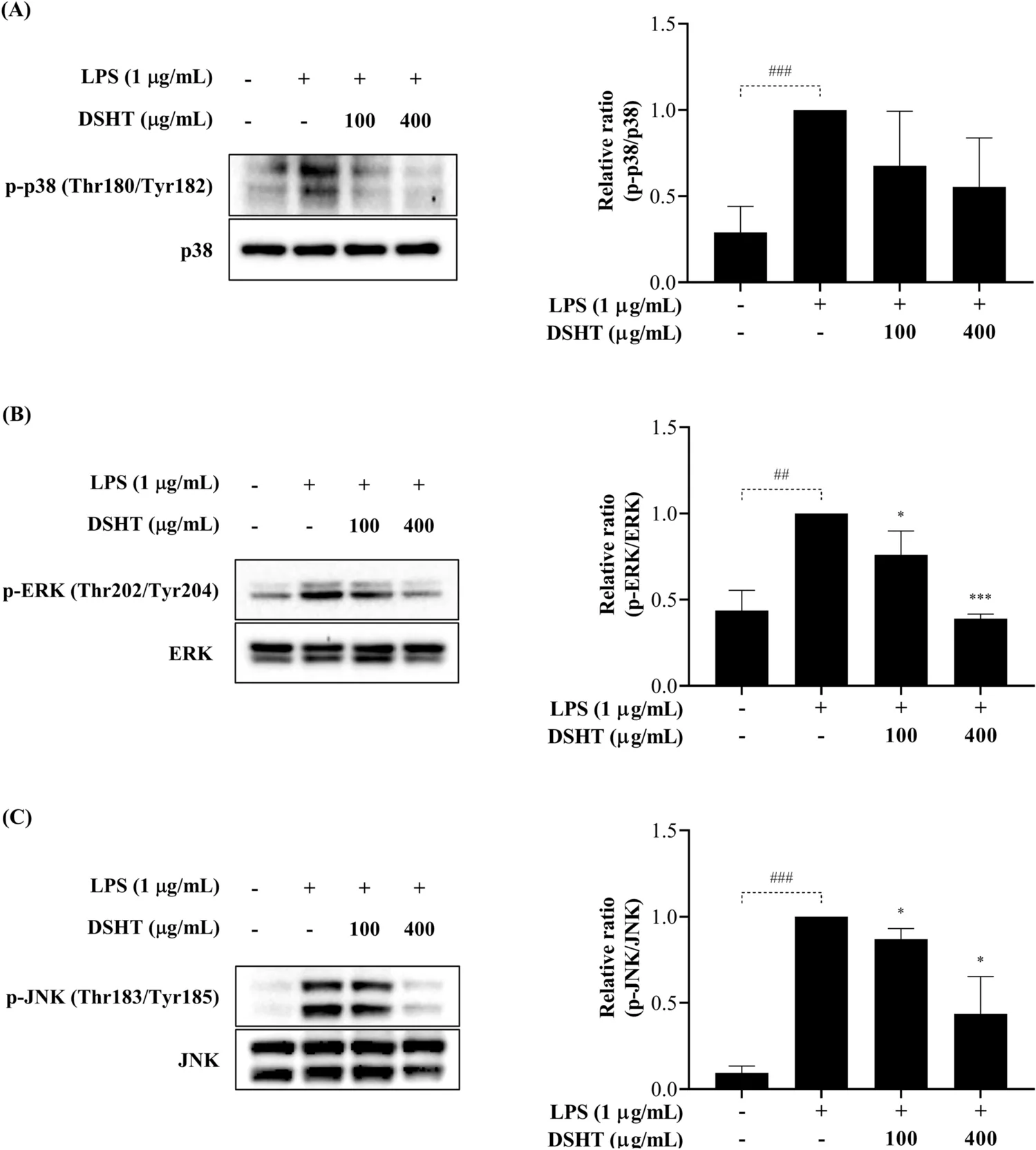
Effect of DSHT on the phosphorylation of p38 (**A**), ERK (**B**), and JNK (**C**) pathways in LPS-stimulated RAW 264.7 macrophages. Cells were treated with the indicated concentration of DSHT (100 and 400 μg/mL) and stimulated with LPS (1 μg/mL) for 30 min. Data are presented as mean ± SD (n = 3). ^##^*p* < 0.01 and ^###^*p* < 0.001 vs. control; *p < 0.05 and ***p < 0.001 vs. LPS.

We further examined whether the impact of DSHT’s anti-inflammatory effect is involved in MAPK phosphorylation using LPS-stimulated p38 MAPK (p38), extracellular signal regulated kinases (ERK), and c-Jun N-terminal kinase (JNK) (Fig. 5A–C). Foreseeably, LPS significantly promotes phosphorylation of these proteins (*p* < 0.01 and *p* < 0.001). DSHT concentration-dependently decreased the ERK and JNK phosphorylation and were reduced to 39.2% (*p* < 0.001) and 56.2% (*p* < 0.05), respectively, at 400 μg/mL. However, the phosphorylation of p38 was not significantly reduced by DSHT.

### DSHT blocks the transduction of LPS-mediated TLR4 pathway

**Figure 6:**
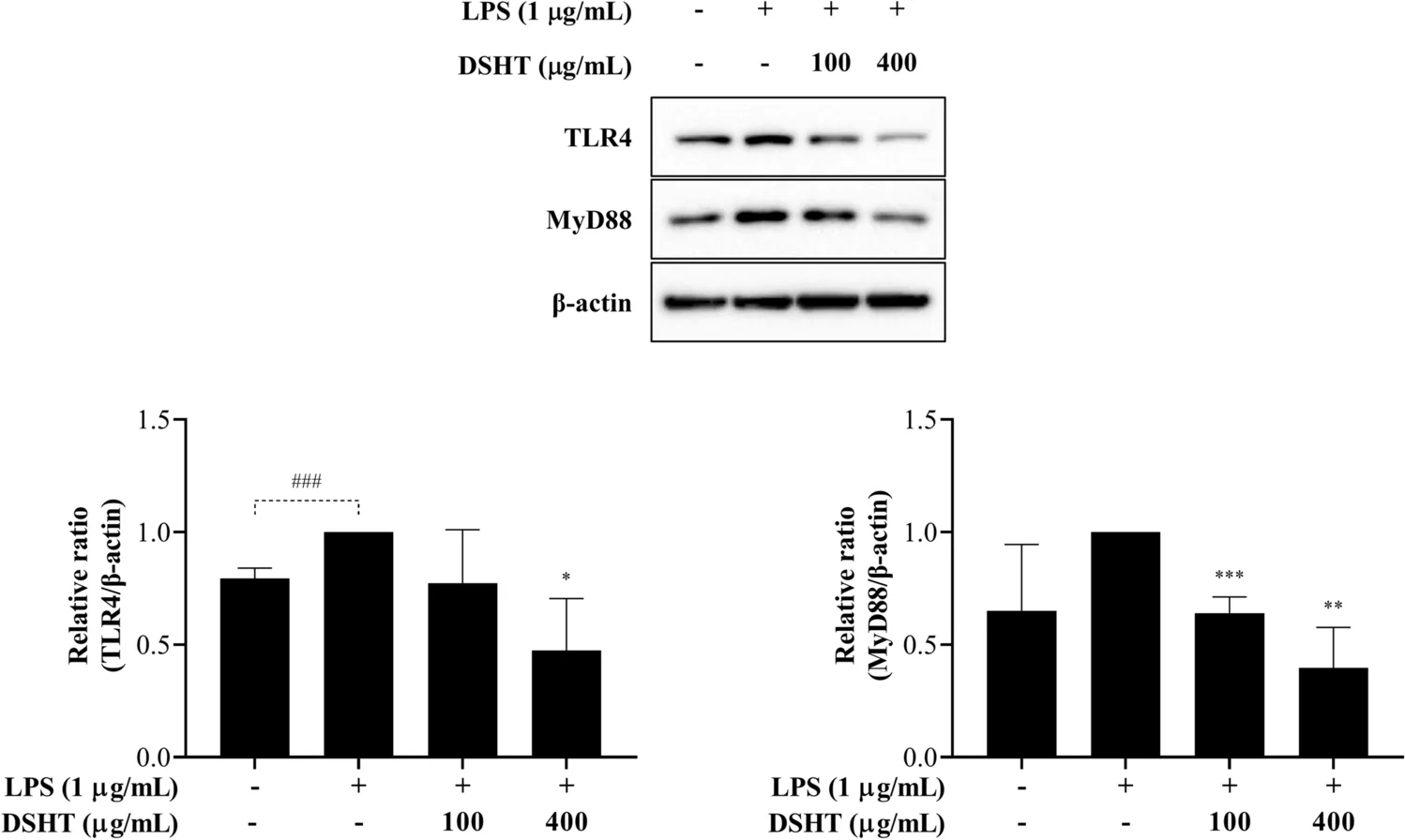
Effect of DSHT on TLR4/MyD88 pathways in LPS-stimulated RAW 264.7 macrophages. Cells were treated with the indicated concentration of DSHT (100 and 400 μg/mL) and stimulated with LPS (1 μg/mL) for 8 h. Data are presented as mean ± SD (n = 3). ^###^*p* < 0.001 vs. control; *p < 0.05, **p < 0.01, and ***p < 0.001 vs. LPS.

To verify whether the impact of DSHT on the regulation of NF-κB and MAPK in terms of its anti-inflammatory effects was related to the TLR4/MyD88 pathways, we assessed the effect of DSHT on TLR4 and MyD88 expression. As illustrated in Fig. 6, the TLR4 and MyD88 expressions were increased by LPS, but there was no significant induction observed in MyD88 expression. Meanwhile, TLR4 expression revealed a tendency to be significant following LPS stimulation. Akin to the MAPK results, both the TLR4 and MyD88 expressions decreased by DSHT in concentration-dependent manner and were reduced to 52.9% (*p* < 0.05) and 60.3% (*p* < 0.01), respectively, at 400 μg/mL, but there was no significant reduction in MyD88 at 100 μg/mL.

### DSHT regulates ABTS radical scavenging activity

**Figure 7:**
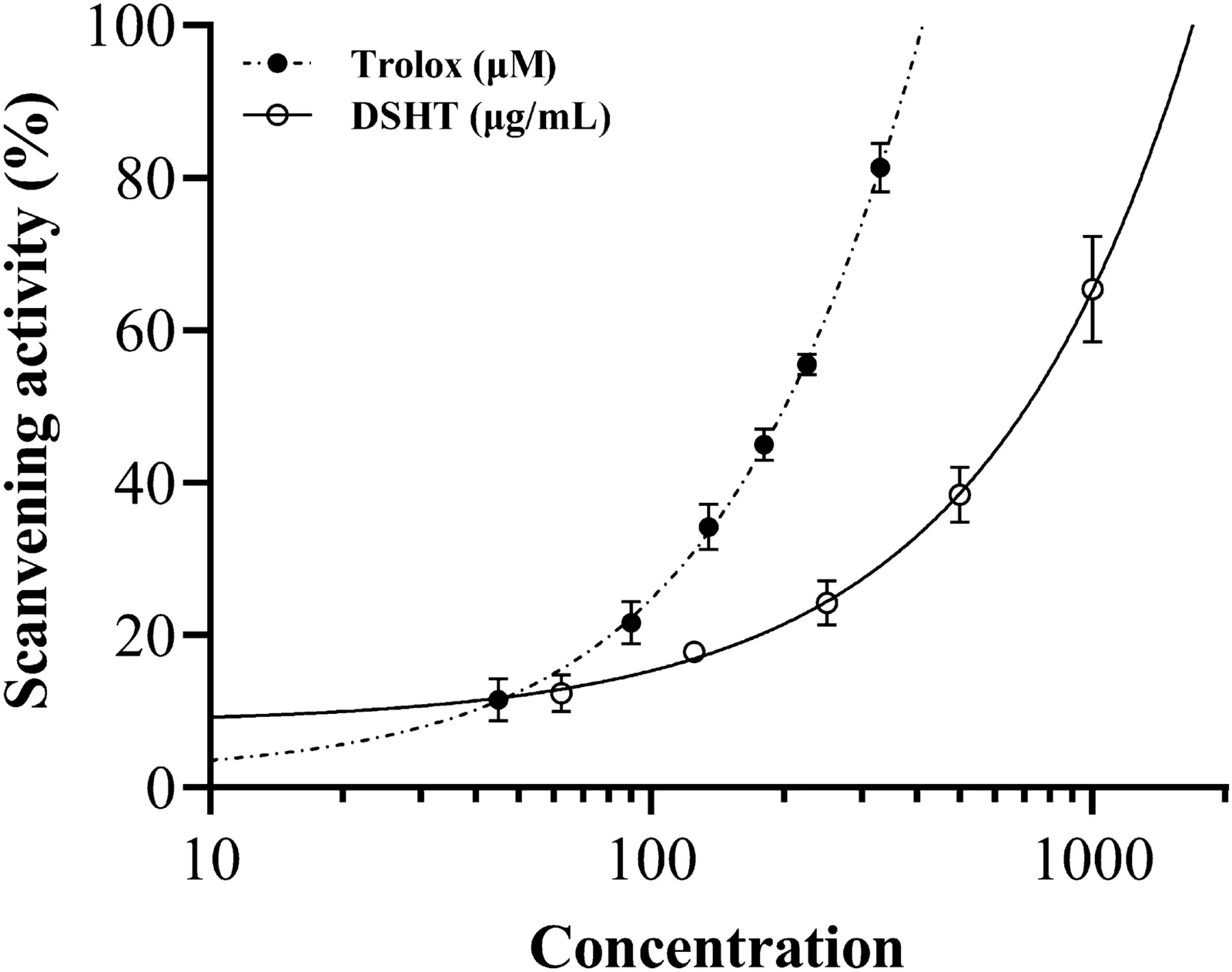
ABTS radical scavenging effects of indicated concentrations of trolox (45–330 μM) (**A**) and DSHT (62.5–1000 μg/mL) (**B**). Data are presented as mean ± SD (n = 4).

To elucidate whether the impact of DSHT’s activity is associated with the oxidative stress, we evaluated 2,2'-Azino-bis-3-ethyl benzothiozoline-6-sulfonic acid (ABTS) radical scavenging activity (Fig. 7). Trolox, which served as a free radical scavenger, exhibited potent ABTS radical scavenging activity and significantly increased by up to 80%. Similarly, DSHT showed potent scavenging activity against ABTS radical and remarkably induced by up to 70%. The IC_50_ values of trolox and DSHT were 200.9 μM and 710.0 μg/mL, respectively. These results showed that DSHT can regulate oxidative stress by activating ABTS radical scavenging.

### DSHT induces LPS-triggered Nrf2/HO-1 pathway

**Figure 8:**
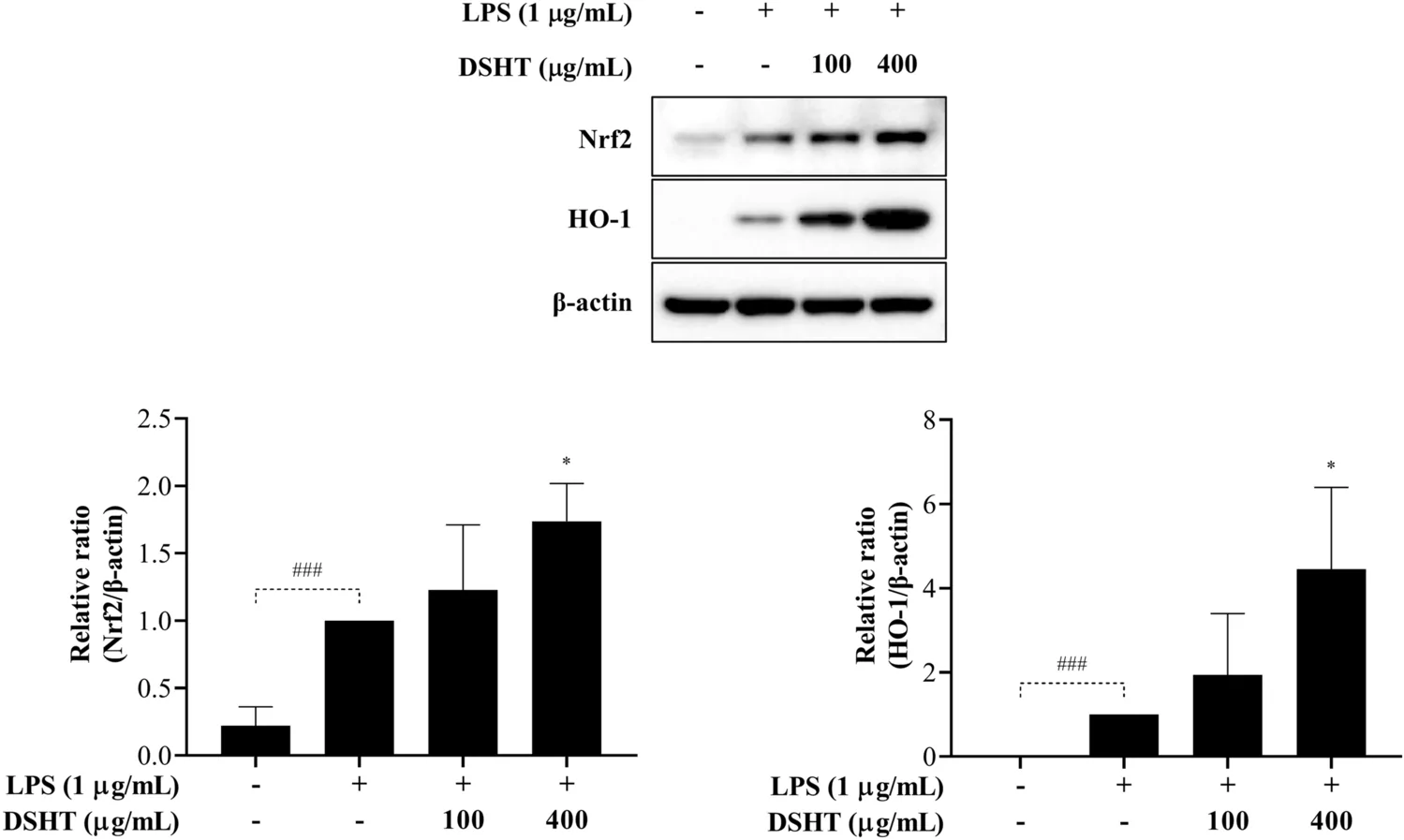
Effect of DSHT on the expression of Nrf2/HO-1 pathway in LPS-stimulated RAW 264.7 macrophages. Cells were treated with the indicated concentration of DSHT (100 and 400 μg/mL) and stimulated with LPS (1 μg/mL) for 18 h. Data are presented as mean ± SD (n = 3). ^###^*p* < 0.001 vs. control; *p < 0.05 vs. LPS.

To investigate whether the regulatory effect of DSHT on oxidative stress was related to the activation of Nrf2/HO-1, we analyzed the nuclear factor-erythroid 2 p-45-related factor 2 (Nrf2) and heme oxygenase 1 (HO-1) expression (Fig. 8). Based on results, Nrf2 and HO-1 were slightly increased following LPS treatment. Co-treatment with LPS and DSHT dose-dependently induced these proteins, which indicates Nrf2 activation. Furthermore, the Nrf2 and HO-1 expressions showed a 1.7-fold (*p* < 0.05) and 4.5-fold (*p* < 0.05) induction at 400 μg/mL, respectively, and this effect was significant.

## Discussion

In this study, we used an LPS-stimulated macrophages to ascertain whether DSHT can modulate the inflammatory response and oxidative stress. The results indicated that DSHT has anti-inflammatory impact induced by LPS through regulating inflammatory signaling and oxidative stress, such as TLR4/MyD88, NF-κB, MAPK, and Nrf2/HO-1 pathways.

Excessive release of inflammatory cytokine from activated macrophages make them a potential target for regulating inflammation and inflammatory diseases^31^. NO, synthesized from _L_-arginine through the iNOS, holds a pivotal role in physiological conditions. However, LPS-stimulated macrophages overexpress iNOS, causing excessive NO production. This not only contributes to the development of inflammatory disease but also induced oxidative stress^32,33^. Meanwhile, COX-2, a catalytic enzyme, converts arachidonic acid to generate PGE_2_, and excessive PGE_2_ is induced by COX-2 leads to various inflammatory responses^16,33,34^. Suppression of NO and PGE_2_ has emerged as a promising therapeutic strategy against inflammation-related disorders. Our findings demonstrated that DSHT dose-dependently inhibited LPS-induced NO and PGE_2_ secretion in macrophages, which was related with the inhibition of iNOS and COX-2.

Among the macrophage activation-associated intercellular signaling pathways, transcription factor NF-κB is crucial for regulating the inflammatory response. In a physiological setting, it remains inactive in the cytoplasm while complexed with IκBα^35,36^, however, upon exposure to irritant such as LPS, NF-κB translocate into the nucleus, triggering the transcriptional activation of inflammation-related genes. Another key player, MAPK, a serine/threonine protein kinase, includes p38, ERK, and JNK. These kinases influence various cellular processes including proliferation, stress response, inflammation, and apoptosis. Furthermore, MAPK orchestrated the regulation of pro-inflammatory mediators and expression of cytokines^37^. The cell surface receptor, TLR4, induced by LPS, is capable of recruiting MyD88 to activate NF-κB and MAPKs. To understand the regulatory mechanism underlying the effect of DSHT on the LPS-stimulated inflammatory response, we assessed the expression level of TLR4/MyD88, NF-κB, and MAPK pathways. Our results revealed that DSHT reduced LPS-triggered TLR4 and MyD88 expression. Furthermore, DSHT exhibited the ability to modulate the phosphorylation of ERK and JNK, suggesting its role in the inhibition of MAPK signaling pathway on LPS-triggered macrophage activation. Additionally, in the nucleus, DSHT significantly reduces the expression level of NF-κB in LPS-stimulated macrophages while restoring the expression level of degraded IκBα in the cytosol, which is indicative of NF-κB inactivation. These observations suggest that the effect of DSHT on inflammatory response are mediated through NF-κB and MAPK signaling transductions, elucidating the molecular mechanism underlying DSHT’s beneficial effects.

Oxidative stress, an imbalance between oxidants and antioxidants, arises from excessive production of ROS. While ROS are essential molecules for biological processes, their overproduction contributes to the production of inflammatory cytokines and progression of inflammatory-related disease^4,18^. Therefore, the regulation of oxidative stress is important to maintain a balance in ROS generation. ABTS, commonly used to measure radical scavenging activity, reacts with potent antioxidants. DSHT induced a dose-dependent ABTS radical scavenging activity, demonstrating its antioxidant effects. HO-1, recognized for its pivotal role in the antioxidant pathway^38^, Also exerts control over the generation of anti-inflammatory cytokines and mediators^39,40^. Nrf2, major regulator of redox homeostasis, is also involved in oxidative stress and inflammatory response through the induction of detoxifying enzyme including HO-1. This study revealed that DSHT slightly induced the expression of Nrf2 and HO-1 in the presence of LPS. Furthermore, co-administration of LPS and DSHT significantly increased their expression level compared to LPS alone. These observations collectively demonstrate that the ABTS radical scavenging effect of DSHT was associated with the enhancement of HO-1 through Nrf2 activation in the presence of LPS, thus alleviating oxidative stress.

## Conclusion

Our study demonstrated that DSHT regulated LPS-induced inflammatory response and mitigated oxidative stress, a modulation of intricately linked to the suppression of TLR4/MyD88, NF-κB, MAPK, and Nrf2/HO-1 signaling transductions. These findings collectively underscored the potential pharmacological benefits of DSHT for the prevention and treatment of disorders related to inflammation and oxidative stress.

## Method

### Materials

The following materials were purchased for the study: Dulbecco’s modified Eagle’s medium (DMEM), fetal bovine serum (FBS), and penicillin and streptomycin (P/S) from Gibco BRL (Grand Island, NY, USA); LPS (*Escherichia coli*; O111:B4); L-NMMA, indomethacin and radioimmunoprecipitation assay lysis buffer (RIPA) from Sigma-Aldrich (St. Louis, MO, USA); CCK-8 from Dojindo (Kumamoto, Japan); Griess reagent from Promega (Madison, WI, USA); 2,2′-Azino-bis-3-ethyl benzothiozoline-6-sulfonic acid (ABTS)-based antioxidant kit and PGE_2_ enzyme-linked immunosorbent assay (ELISA) detection kit from Cayman (Ann Arbor, MI, USA); Primary antibodies from Cell Signaling Technology (Beverly, MA, USA) against iNOS (#13120), COX-2, NF-κB p65, IκBα, phospho-p38 (Thr180/Tyr182), p38, phospho-ERK (Thr202/Tyr204), ERK, phospho-JNK (Thr183/Tyr185), JNK, β-actin, GAPDH, Lamin B1, MyD88, Nrf2, and HO-1, and TLR4 from Santa Cruz Biotechnology (Dallas, TX, USA); horseradish peroxidase (HRP)-conjugated secondary antibodies against rabbit and mouse from Jackson ImmunoResearch (West Grove, PA, USA); Halt protease & phosphatase inhibitor cocktail and NE-PER nuclear and cytoplasmic extraction reagents from Thermo Fisher Scientific (Rockford, IL, USA); ECL chemiluminescence from GE Healthcare (Chicago, IL, USA). Polyvinylidene fluoride membrane from Millipore (Kenilworth, NJ, USA).

### Preparation of DSHT extract

The DSHT extract was prepared with water and phytochemical analysis was completed according to Seo and Shin^30^. Briefly, the dried herbal medicines were chopped, mixed, then extracted at 100 °C for 2 h using a COSMOS-660 extractor (Kyungseo E&P, Incheon, Korea) according to *Bangyakhappyeon*^20^. The decoction of DSHT was filtered using a 53 μm sieve, lyophilized by PVTFD100R (IlShinBioBase, Dongducheon, Korea), and stored at – 18 °C in the herbarium of KM Science Research Division, Korea Institute of Oriental Medicine (voucher specimen No. KE61) until use. The amount of albiflorin (CAS NO. 39011-90-0), baicalein (CAS NO. 491-67-8), baicalin (CAS NO. 21967-41-9), benzoic acid (CAS NO. 65-85-0), gallic acid (CAS NO. 149-91-7), naringin (CAS NO. 10236-47-2), paeoniflorin (CAS NO. 23180-57-6), poncirin (CAS NO. 14941-08-3), wogonin (CAS NO. 632-85-9), wogonoside (CAS NO. 51059-44-0) in the DSHT extract were 1.5, 3.78, 45.98, 0.96, 2.62, 5.04, 7.74, 7.55, 0.77, and 8.34 mg/g, respectively^30^.

### Cell culture and morphology assessment

The RAW 264.7 (murine macrophage) cells were from American Type Culture Collection (Rockville, MD, USA). Macrophages were incubated in a DMEM supplemented with 5.5% FBS, and 1% P/S and grown at 37 °C in a humidity chamber containing 5% CO_2_. The morphological change of DSHT was observed in cells stimulated with or without LPS (1 μg/mL) for 18 h and examined using an inverted phase contrast microscope (× 200) (Olympus, Tokyo, Japan).

### Cell proliferation assay

The cells (1 × 10^4^ cells/well) were seeded in a clear 96-well plate and grown overnight. The following day, cells were subjected to a variety of concentrations of DSHT (12.5–400 μg/mL) and incubated for an additional 24 h. To determine cell proliferation, a CCK-8 kit was employed following the manufacturer’s instruction. Absorbance at 450 nm was read using a plate reader (Bio-Rad, Hercules, CA, USA), and cell proliferation was expressed using the following equation:

1 $$\mathrm{Cell \, proliferation }(\mathrm{\%}) =\frac{\text{ DSHT-treated \, cells}}{\text{ Untreated \, cells}}\times 100$$

### Determination of NO and PGE_2_

The cells (7.5 × 10^4^ cells/well) were seeded in a clear 48-well plate and subjected to varying concentrations of DSHT (25–400 μg/mL), L-NMMA (100 μM), and indomethacin (5.6 nM) with or without LPS (1 μg/mL), and incubated for an additional 24 h. The NO synthesis was determined by using Griess reagents. This involved mixing 50 μL of cell culture supernatant with sulfanilamide solution and allowing the mixture to incubate for 10 min at room temperature. Naphthyl ethylenediamine dihydrochloride (NED) was then added to the cells and incubated for 10 min, and absorbance was measured at 540 nm using a microplate reader (Bio-rad). A sodium nitrite standard curve was used to determine nitrite concentration. A commercial PGE_2_ ELISA kit was employed to verify the PGE_2_ level following the manufacturer’s instruction.

### Western blotting

The whole cell extracts were obtained with RIPA buffer, the Halt protease & phosphatase inhibitor cocktail, and PMSF solution. Cytosolic and nuclear extraction kit was used following the manufacturer’s procedure to obtain cytosol (CE) and nuclear extract (NE). To detect the protein expression of iNOS, COX-2, NF-κB p65, IκBα, phospho-p38, p38, phospho-ERK, ERK, phospho-JNK, JNK, Nrf2, HO-1, MyD88, TLR4, β-actin, GAPDH, and Lamin B1, electrophoresis was conducted to separate 20 μg of whole cell extracts, CE, and NE, which were then transferred onto the PVDF membrane. The membranes were blocked with 5% skim milk solution for 1 h and then incubated with the appropriate primary antibody at 4 °C for overnight. After binding of primary antibodies, the membranes were incubated with goat-anti-rabbit IgG-HRP and goat-anti-mouse IgG-HRP secondary antibodies for 1 h at room temperature; the proteins were detected by ECL chemiluminescence and developed using Chemi-Doc instrument (Bio-rad). The quantifications of the protein expression were calculated using Image J 1.53 software (National Institute of Health, Bethesda, MD, USA). The western blot original images are provided as supplementary information available with this article online.

### ABTS radical scavenging activity

The scavenging capacity of DSHT to prevent ABTS oxidation was compared with Trolox, as a positive control. The absorbance was measured at 750 nm wavelength. Each assay was performed using a freshly prepared ABTS solution following the manufacturer’s instruction.

### Statistical analysis

Consistent results were obtained by conducting the experiments at least three or four times. Data are expressed as mean ± standard deviation (SD). Student’s t-test were employed to determine statistical significance. *p*-value lower than 0.05 was considered statistically significant.

## Supplementary Information

Supplementary Figures.

## Data Availability

The data used to support the finding of this study are all included in the article.
