# Correction: Daeshiho-tang attenuates inflammatory response and oxidative stress in LPS-stimulated macrophages by regulating TLR4/MyD88, NF-κB, MAPK, and Nrf2/HO-1 pathways

**DOI:** 10.1038/s41598-026-69985-3

**Published:** 2026-09-15

**Authors:** Yong Jin Oh, Seong Eun Jin, Hyeun‑Kyoo Shin, Hyekyung Ha

**Affiliations:** KM Science Research Division, Korea Institute of Oriental Medicine, 1672 Yuseong‑daero, Yuseong‑Gu, Daejeon, 34054 Korea

Correction to: *Scientific Reports* https://doi.org/10.1038/s41598-023-46033-y, published online 02 November 2023

The original version of the Article contained an error in Figure 1B. Due to the error during figure assembly, the same representative microscopic image was inadvertently used for both the 50 μg/mL and 100 μg/mL DSHT conditions in the LPS (-) group in Figure 1B.

The original Figure 1 and accompanying legend appear below.

**Fig. 1:**
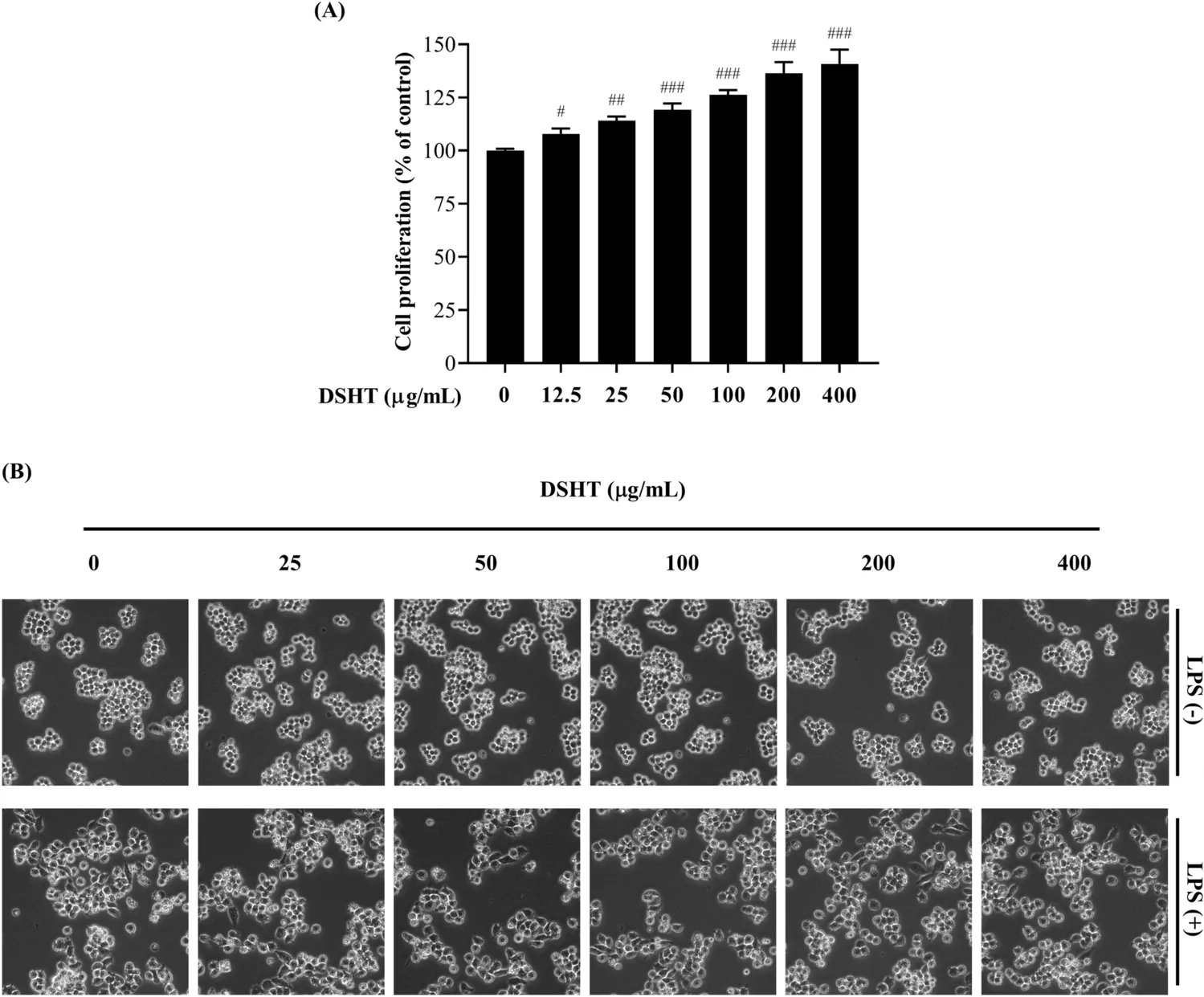
Effect of DSHT on the cell proliferation and morphology of RAW 264.7 macrophages. (**A**) Cells were treated with the indicated concentration of DSHT (12.5–400 μg/mL) for 24 h. Cell proliferation was determined using the colorimetric Cell counting Kit-8 (CCK-8) assay. (**B**) Cell morphology was visualized under an inverted-phase contrast microscope (× 200). Data are presented as mean ± standard deviation (SD) (n = 4). ^#^*p* < 0.05, ^##^*p* < 0.01, and ^###^*p* < 0.001 vs. control.

The original Article has been corrected.

