# Supplementary material for: Daeshiho-tang attenuates inflammatory response and oxidative stress in LPS-stimulated macrophages by regulating TLR4/MyD88, NF-κB, MAPK, and Nrf2/HO-1 pathways
Source: Sci Rep. 2023 Nov 2;13:18891. doi: 10.1038/s41598-023-46033-y (PMC10622541; doi:10.1038/s41598-023-46033-y)
Supplement: Supplementary file 1 — Supplementary Figures. [file 41598_2023_46033_MOESM1_ESM.docx]

**Supplementary Information**

Daeshiho-tang attenuates inflammatory response and oxidative stress in LPS-stimulated macrophages by regulating TLR4/MyD88, NF-κB, MAPK, and Nrf2/HO-1 pathways

Yong Jin Oh, Seong Eun Jin, Hyeun-Kyoo Shin, and Hyekyung Ha*

KM Science Research Division, Korea Institute of Oriental Medicine, 1672 Yuseong-daero, Yuseong-gu, Daejeon 34054, Korea

*To whom correspondence should be addressed. E-mail address: [hkha@kiom.re.kr](mailto:hkha@kiom.re.kr)

• Are you able to provide images showing full length membranes, with membrane edge visible, for this?

- Except for p-p38, p38, p-ERK, ERK, p-JNK, and JNK membranes, other membranes were cut before being incubated with antibodies.

• Were the blots cut prior to hybridization with antibodies?

- Yes, we were cut prior to hybridization with antibodies, but, we cut it from full length membrane and used it.


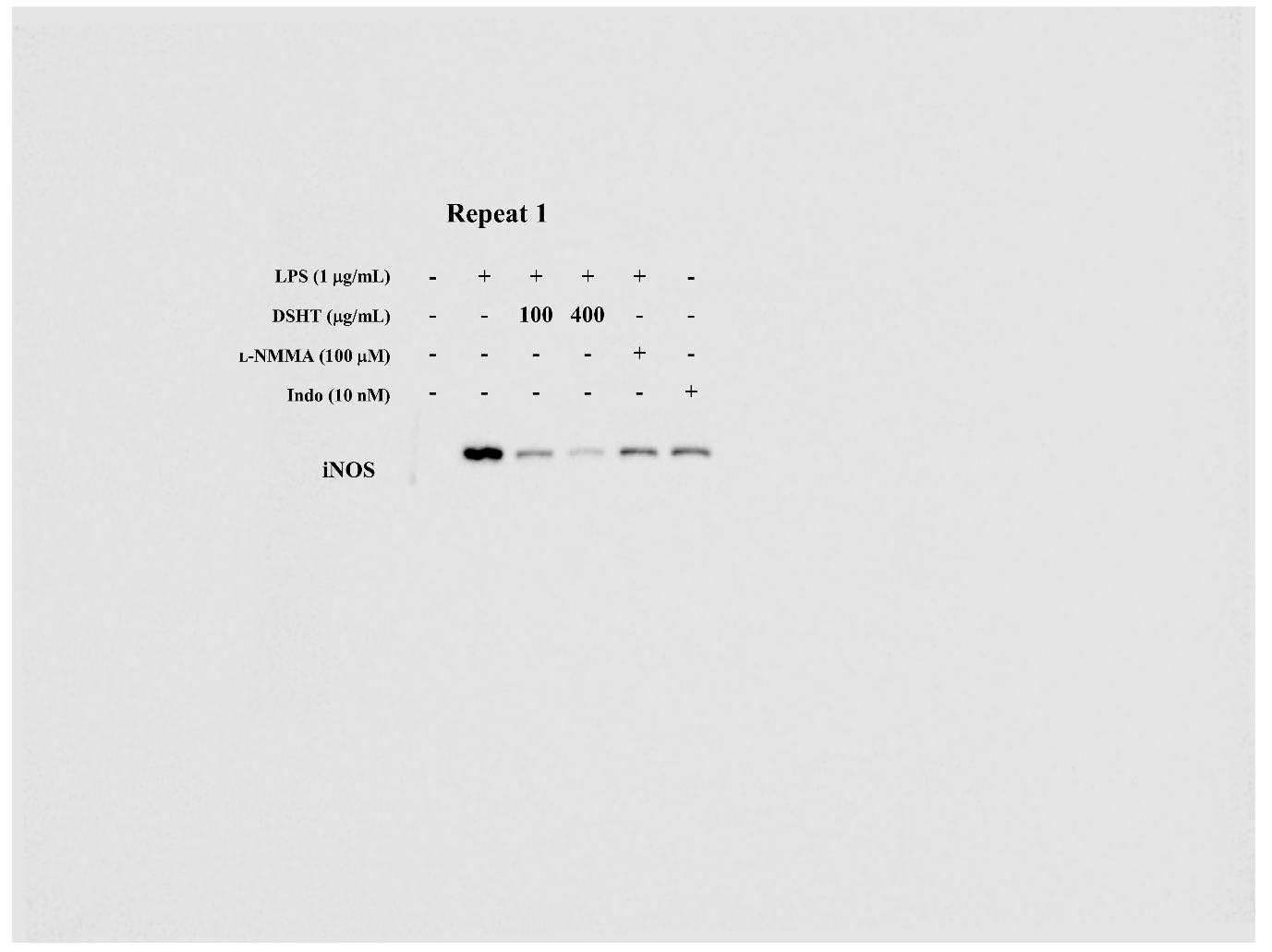


**Supplementary Fig 1**. Full-length western blots for iNOS, COX-2, and β-actin for three repeats. Blot was cut in three before being incubated with specific antibodies. Main figures are displayed using red box.Western blot used for Fig 3.

**
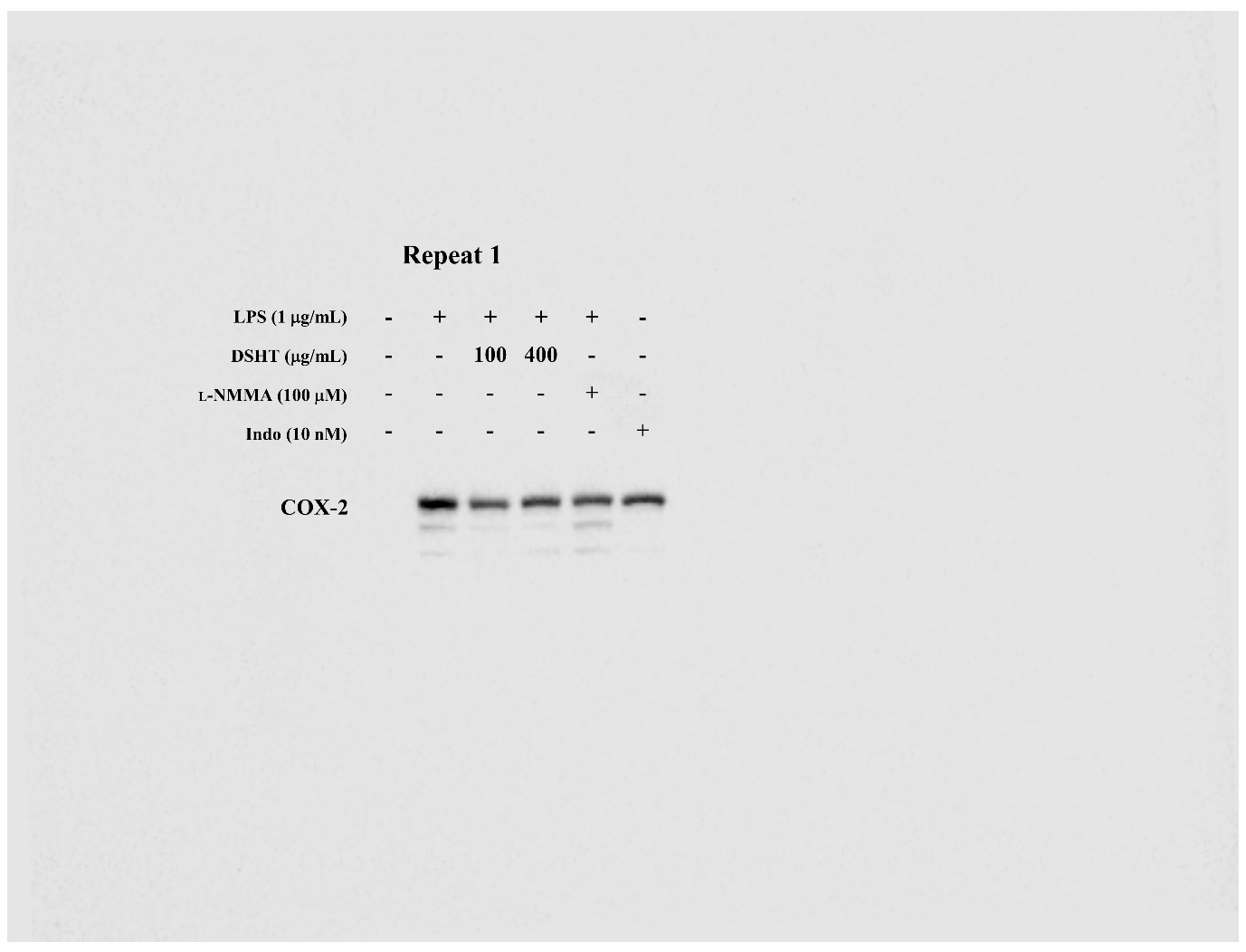
**

**Supplementary Fig 1**. Continued.

**
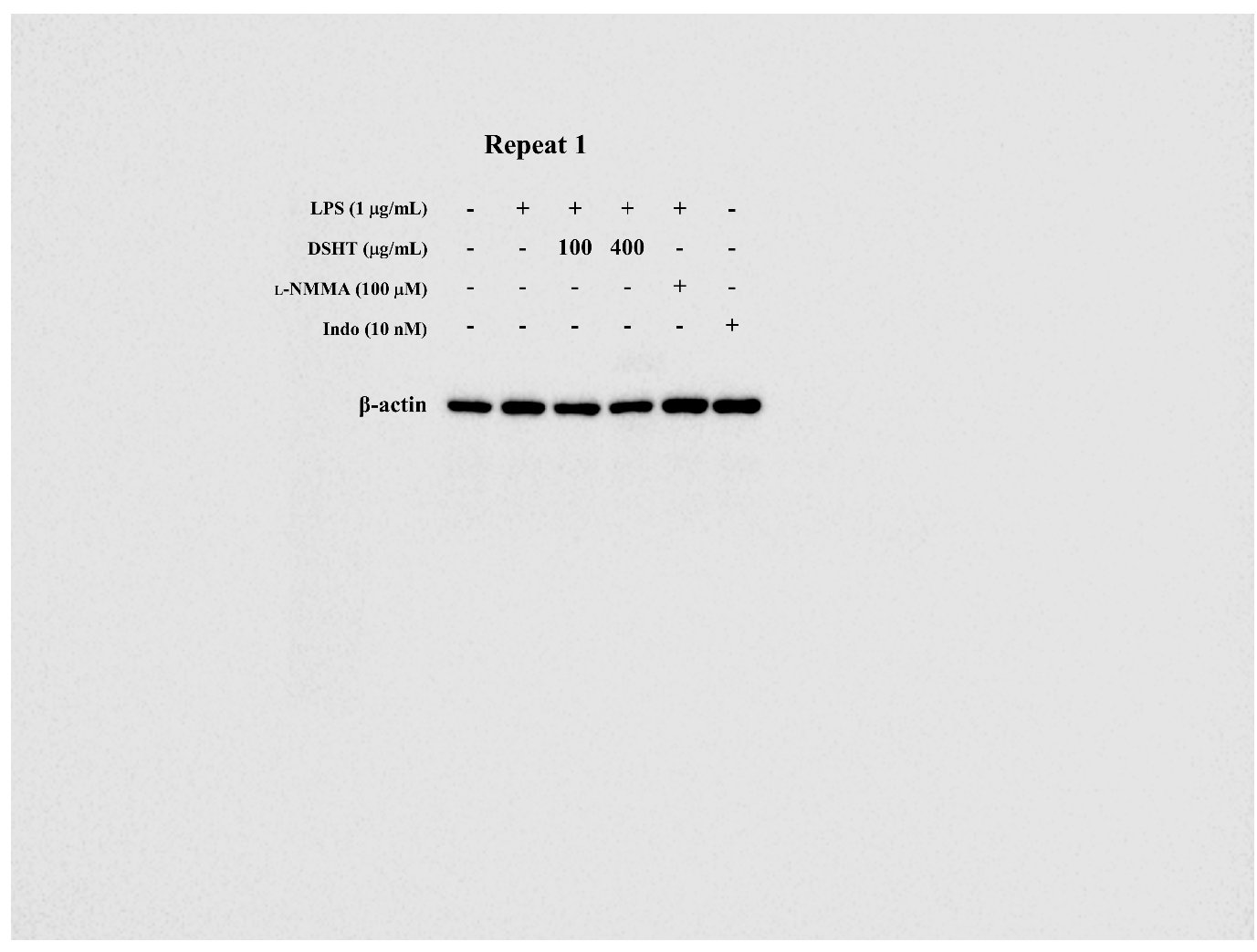
**

**Supplementary Fig 1**. Continued.

**
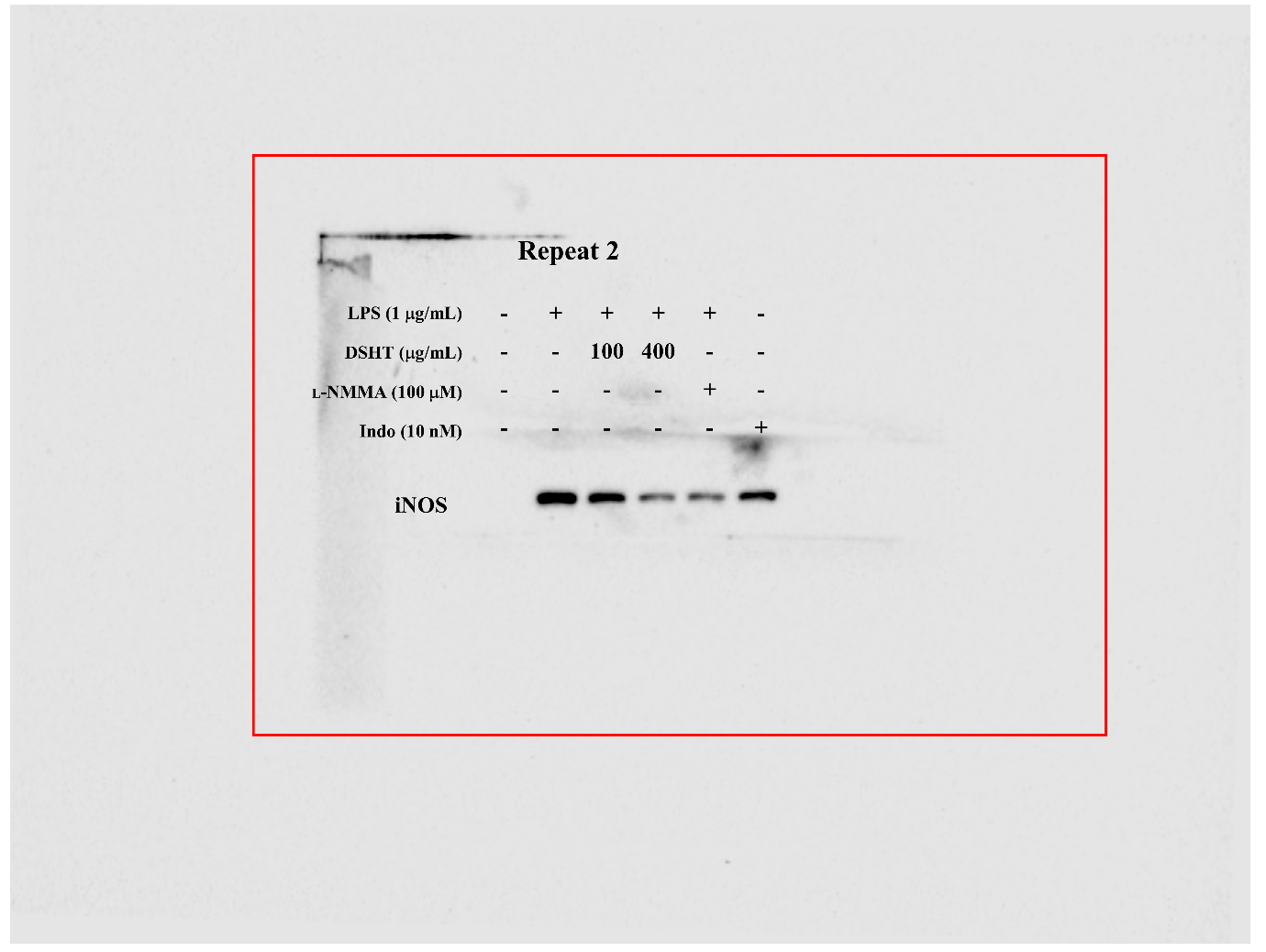
**

**Supplementary Fig 1**. Continued.

**
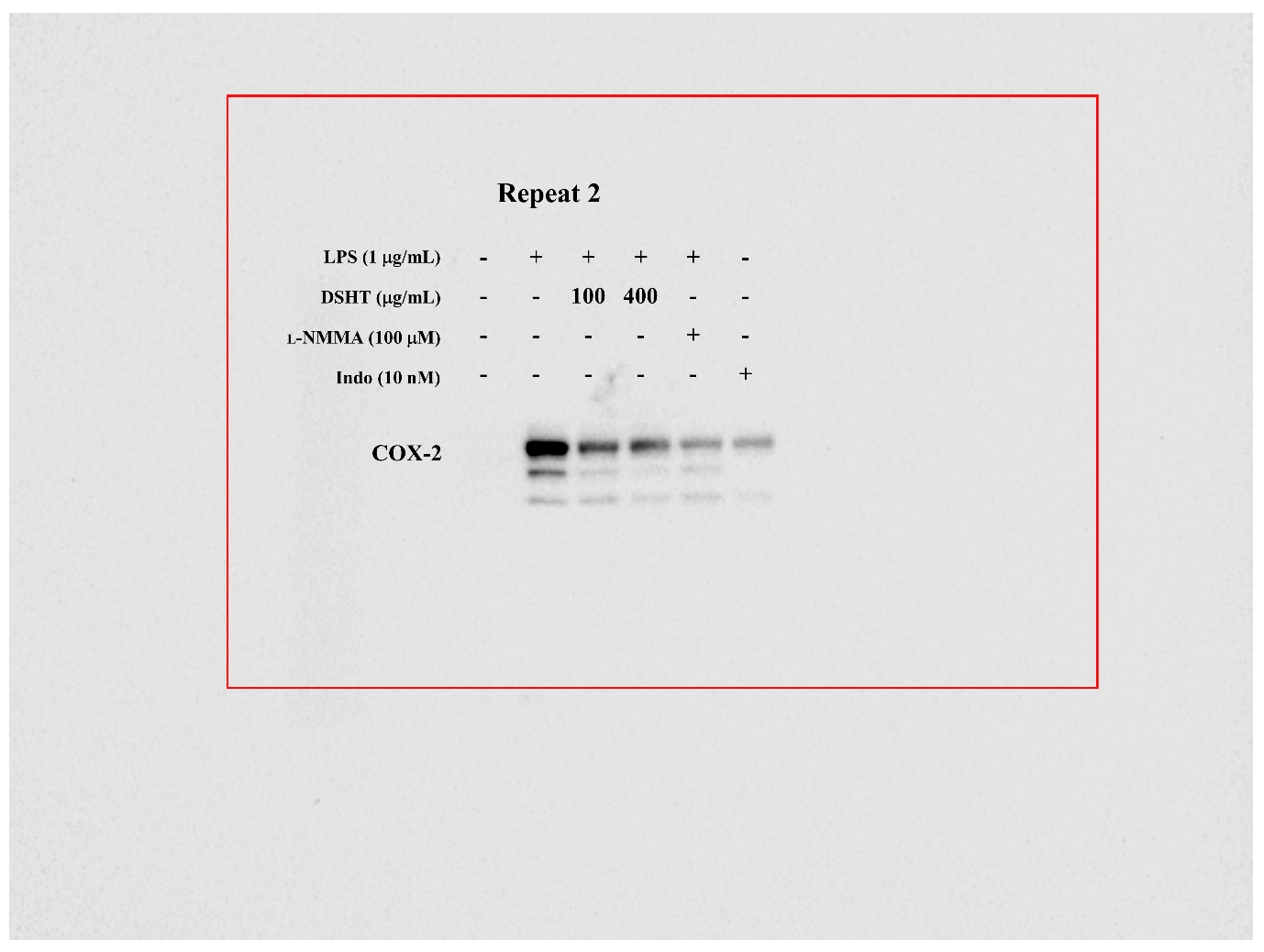
**

**Supplementary Fig 1**. Continued.


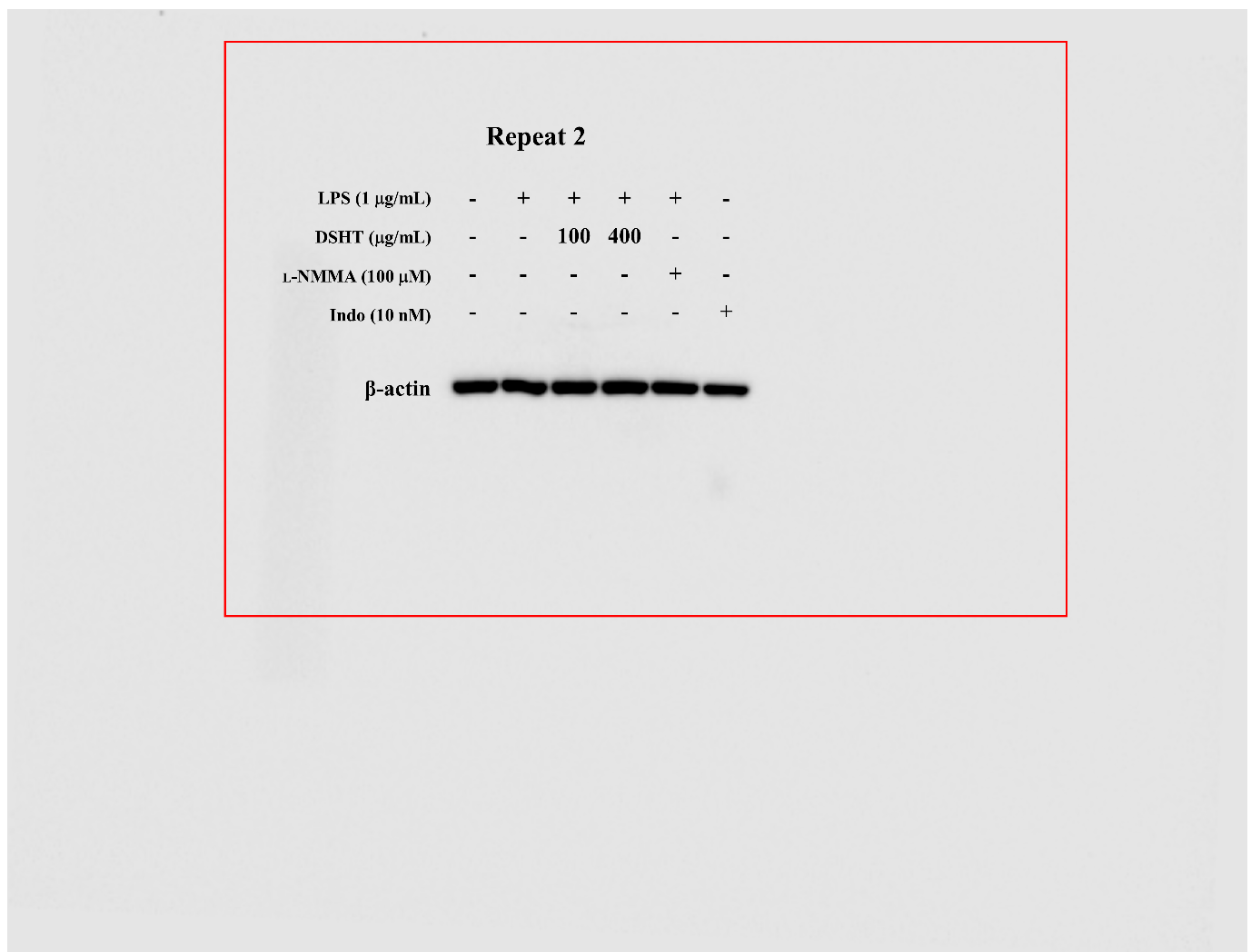


**Supplementary Fig 1**. Continued.


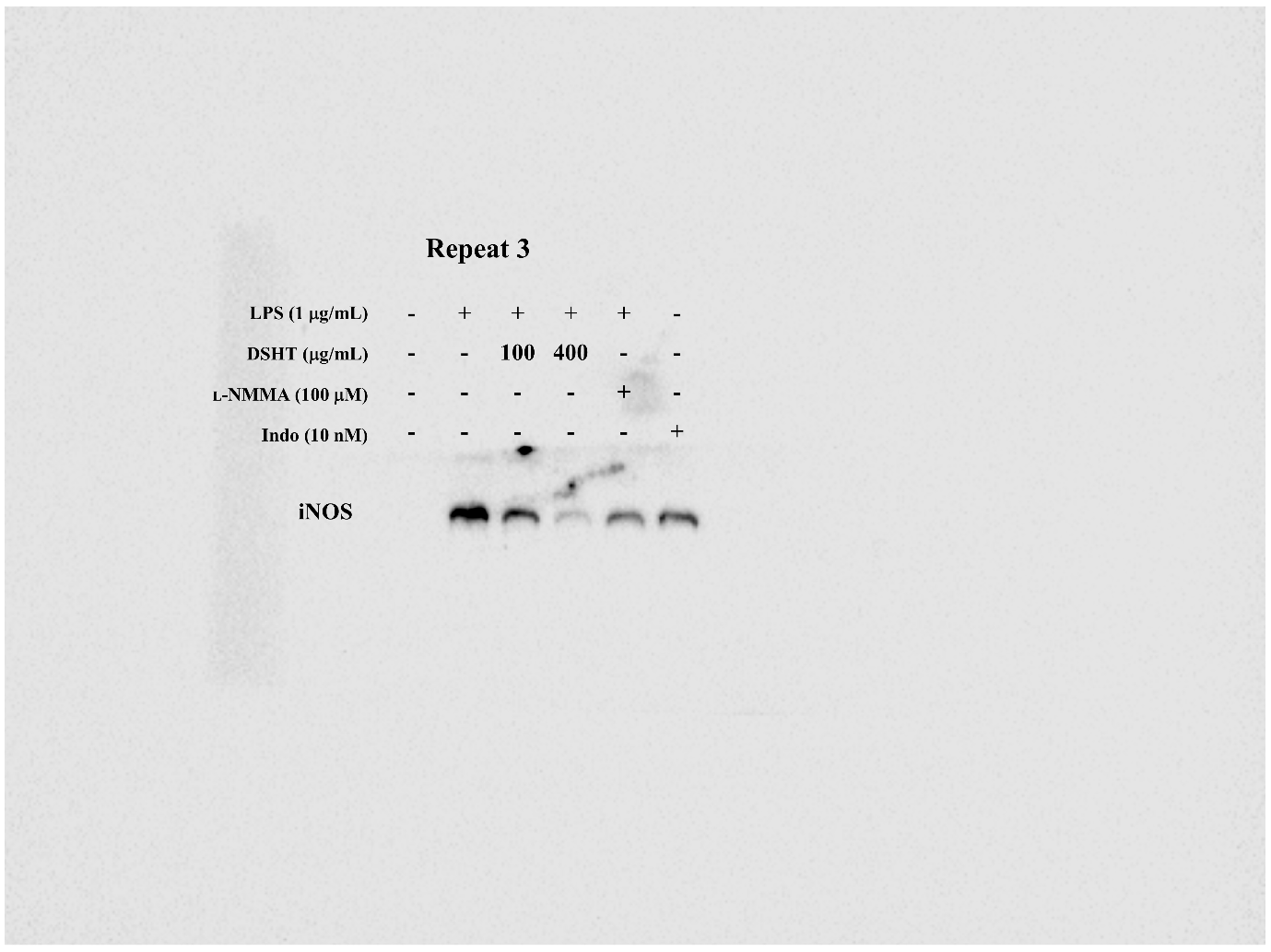


**Supplementary Fig 1**. Continued.


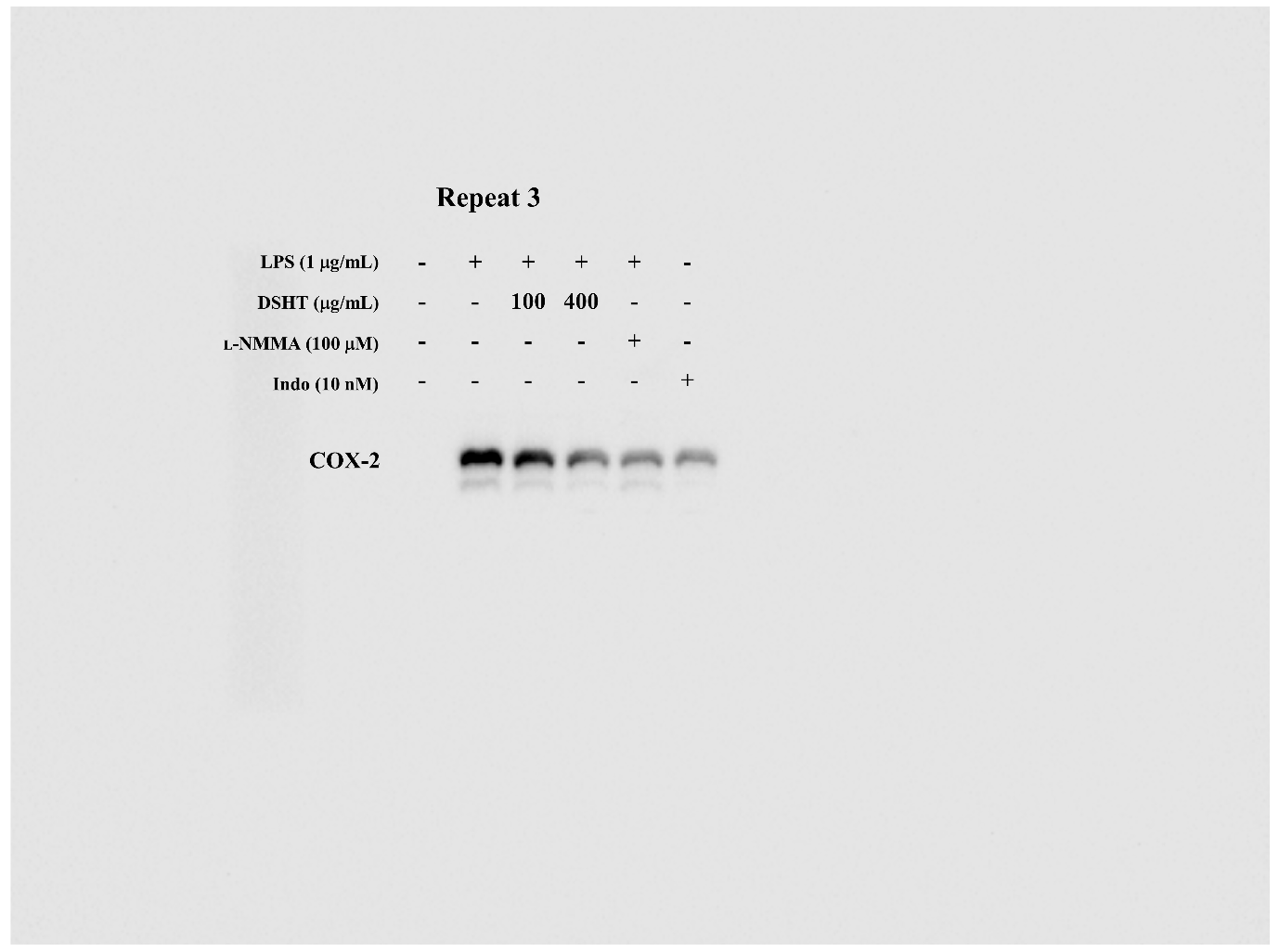


**Supplementary Fig 1**. Continued.


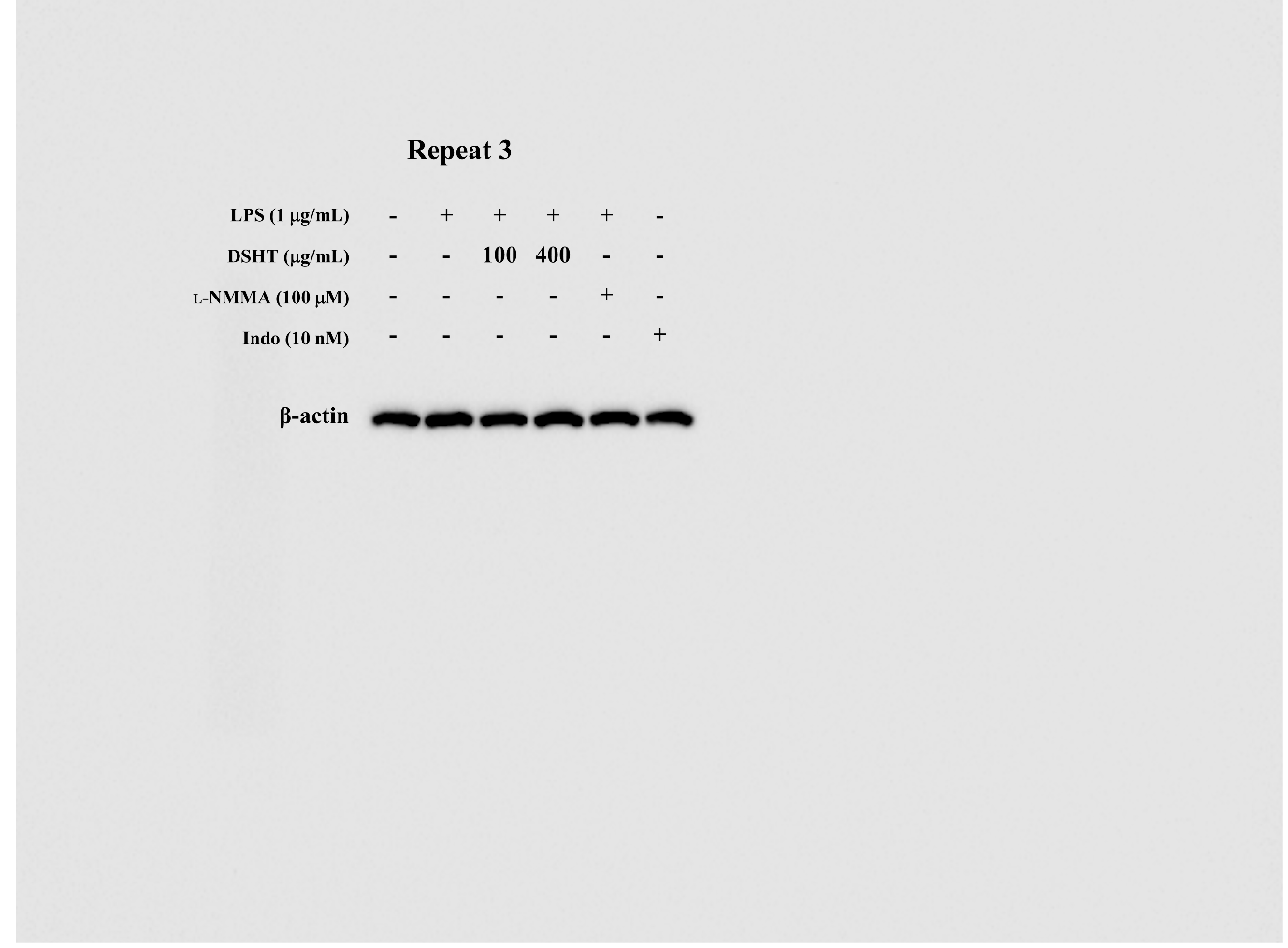


**Supplementary Fig 1**. Continued.

**
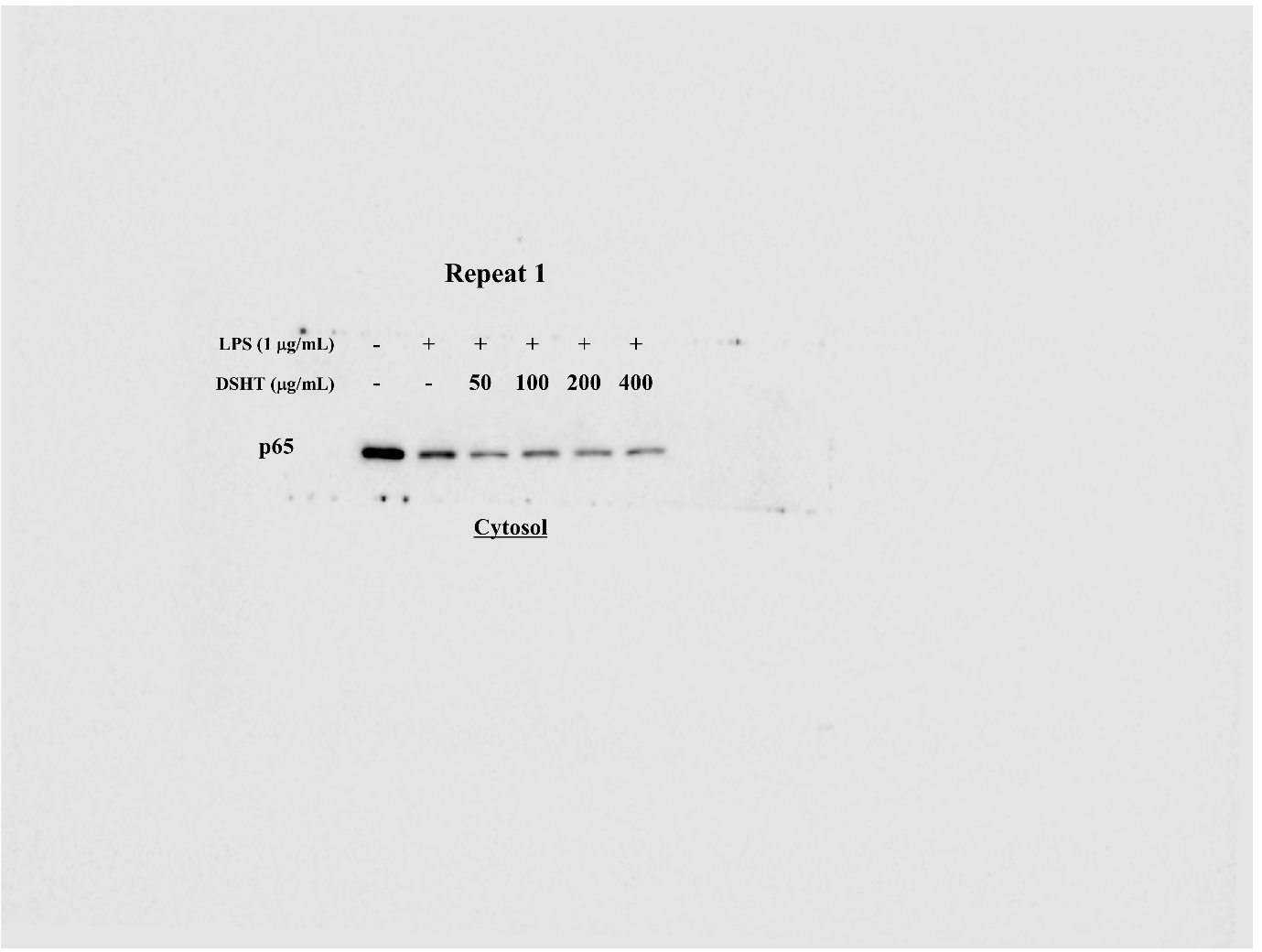
**

**Supplementary Fig 2**. Full-length western blots for NF-κB p65, IκB-α, GAPDH and Lamin B1 for three repeats. Blot was cut in two before being incubated with specific antibodies for NF-κB p65 and IκB-α. After detection, two membrane was stripped before being incubated with specific antibodies for GAPDH and Lamin B1, respectively. Main figures are displayed using red box. Western blot used for Fig 4.

**
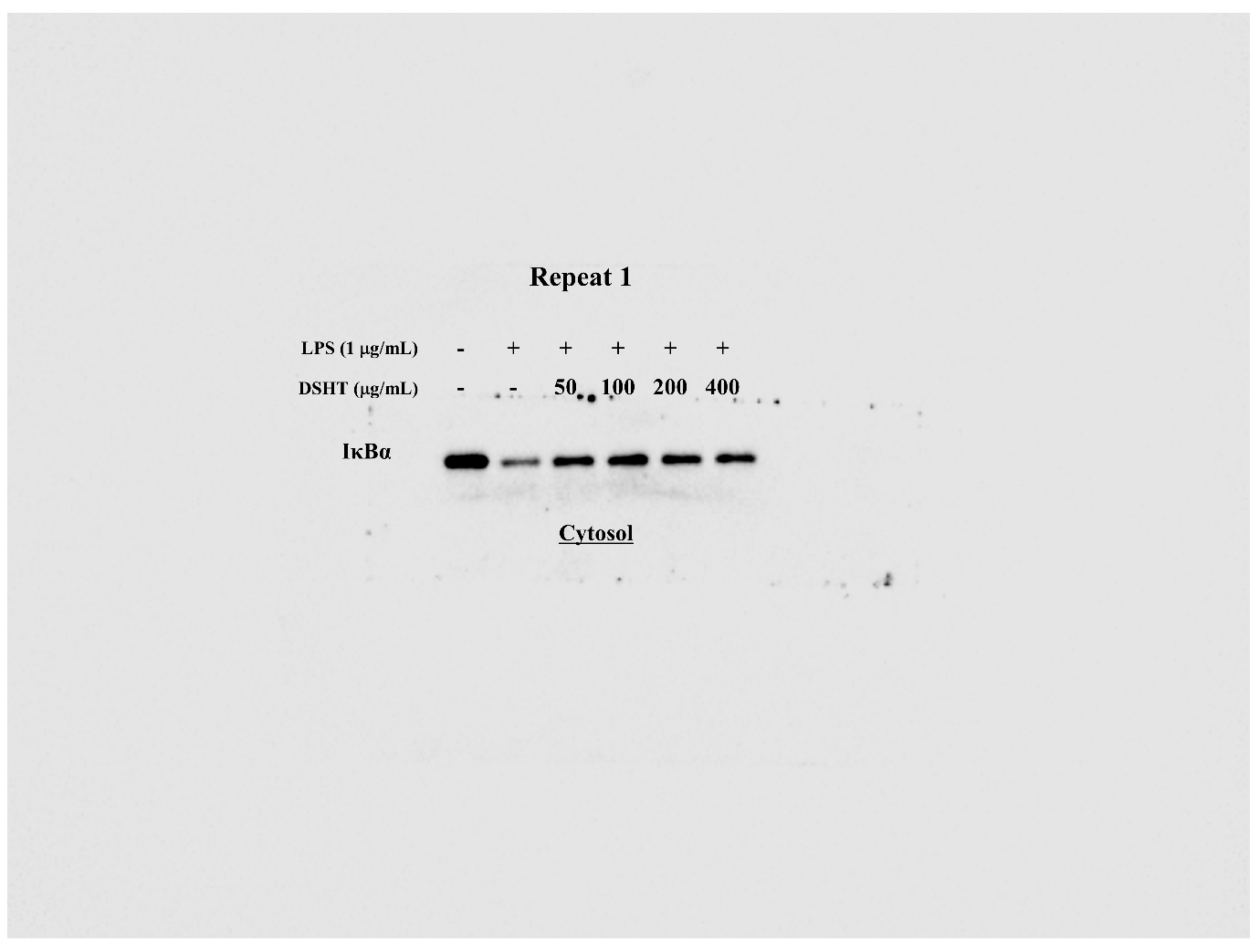
**

**Supplementary Fig 2**. (Continued).


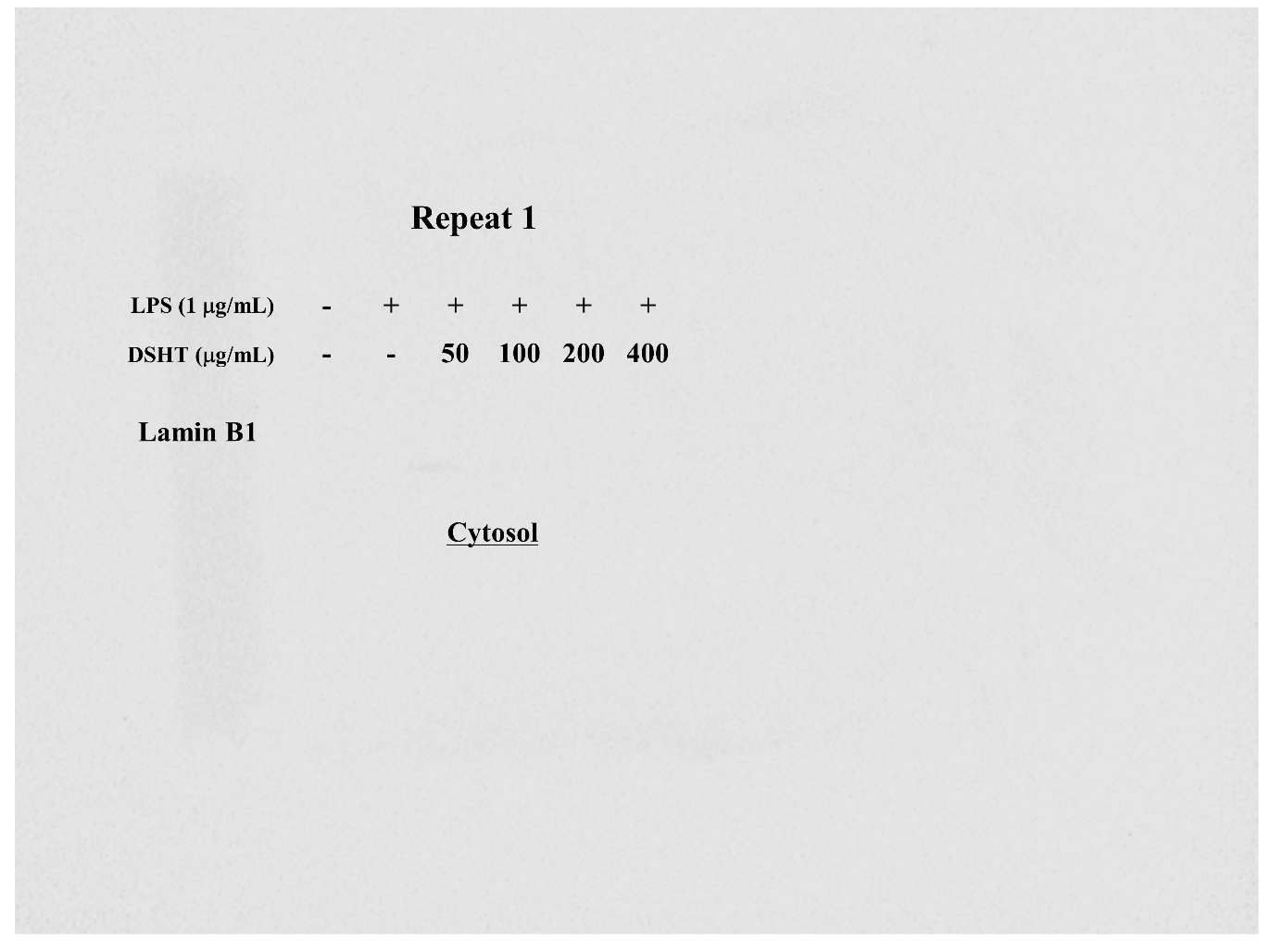


**Supplementary Fig 2**. (Continued).


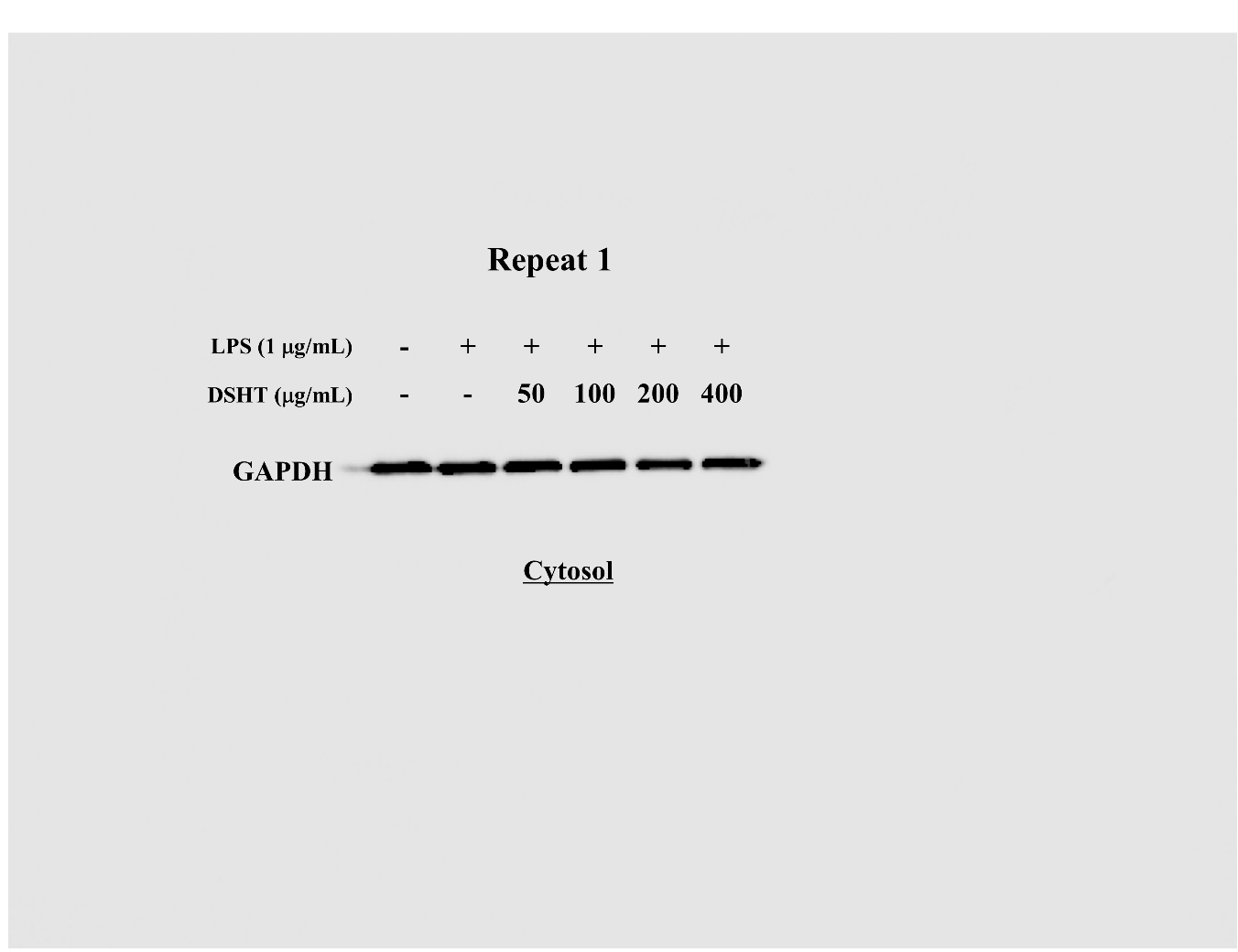


**Supplementary Fig 2**. (Continued).


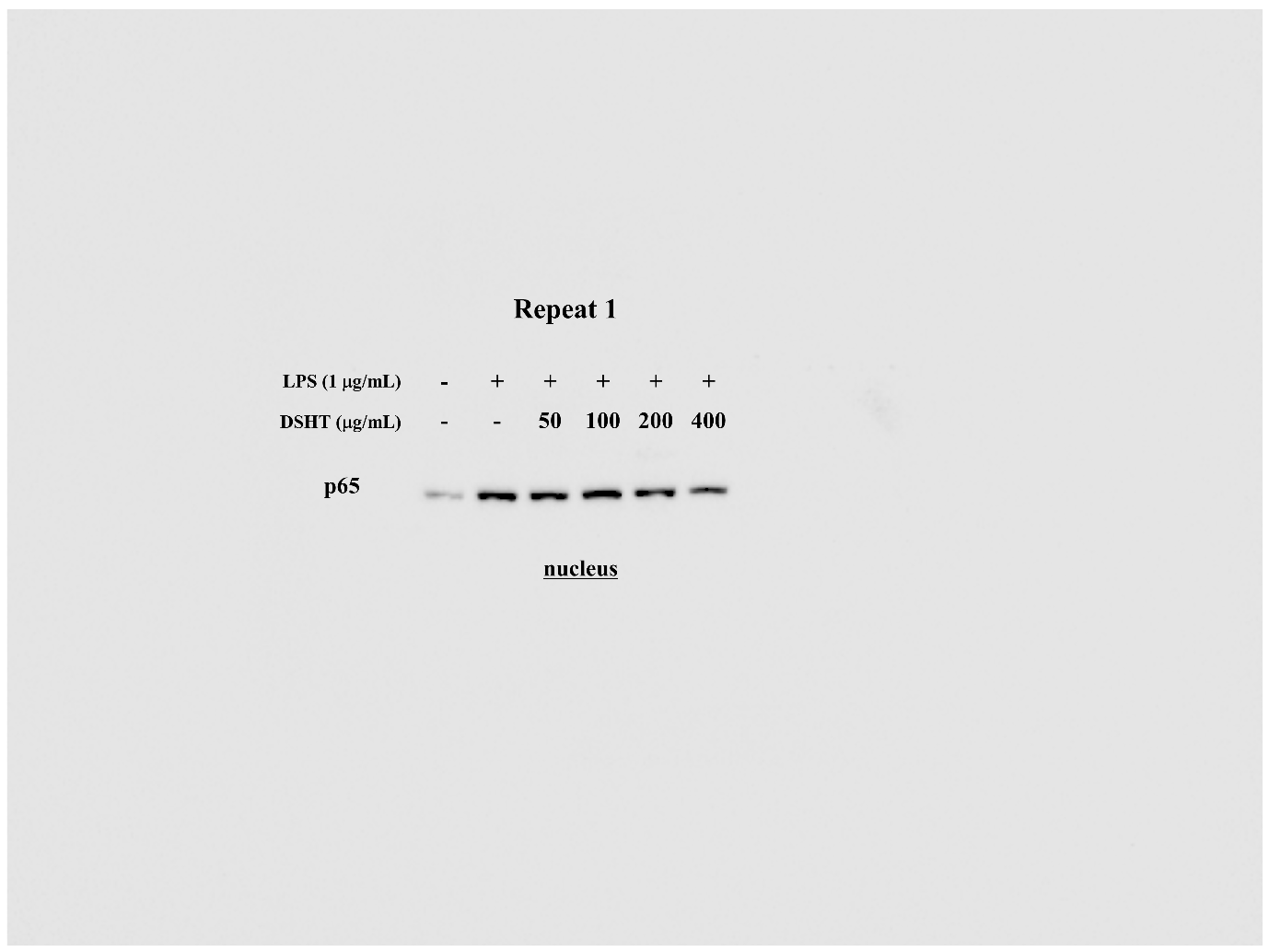


**Supplementary Fig 2**. (Continued).


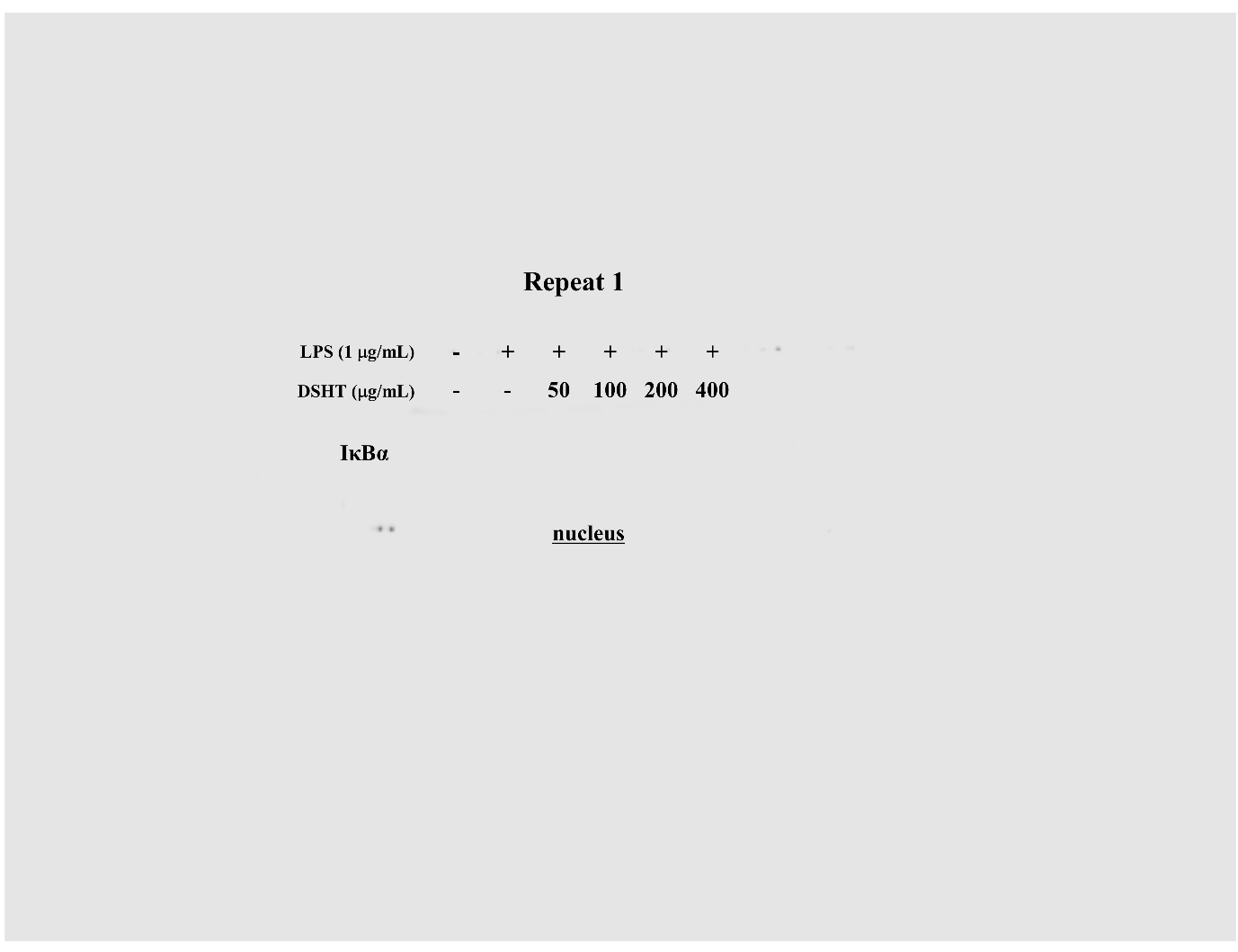


**Supplementary Fig 2**. (Continued).


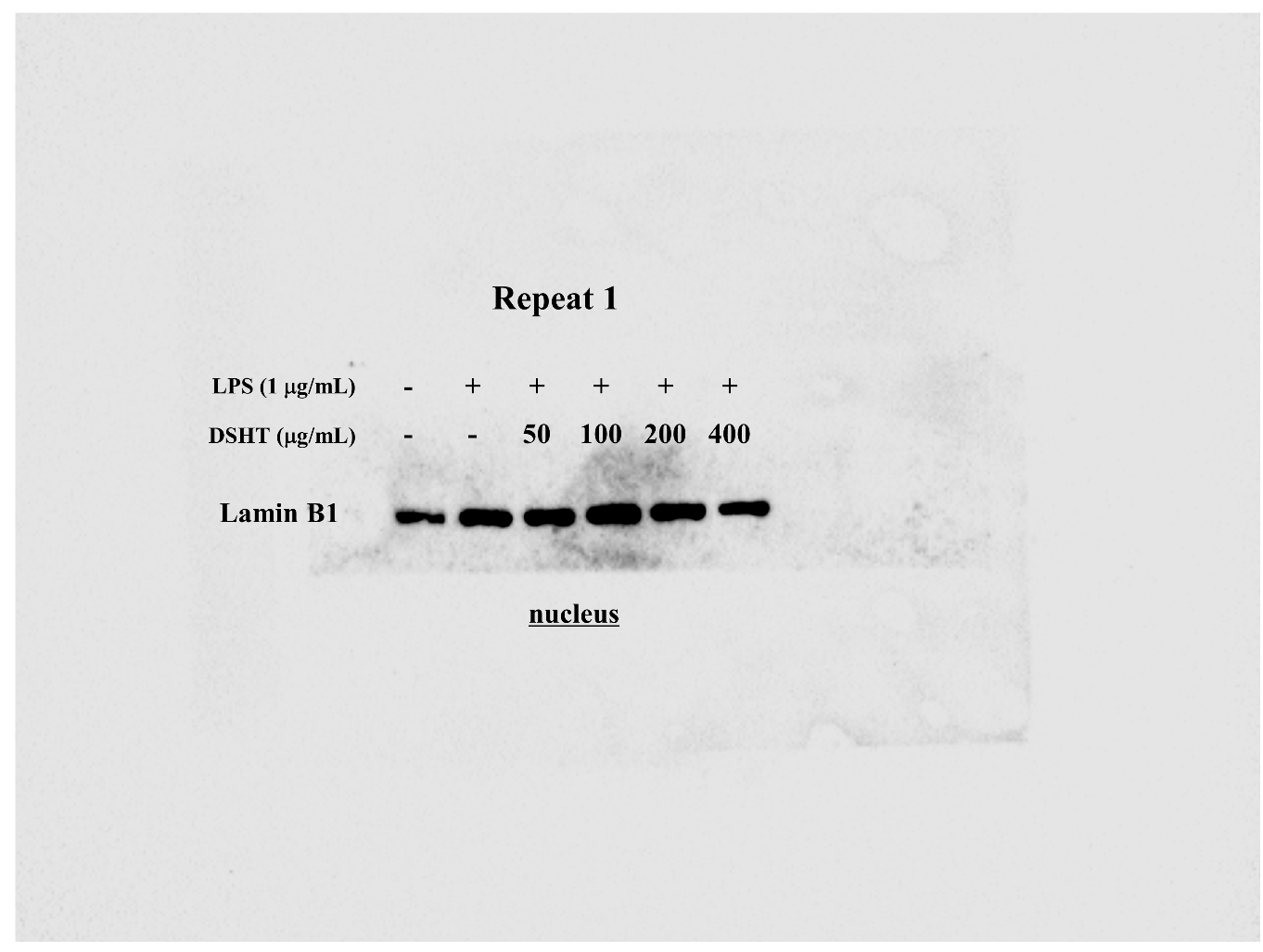


**Supplementary Fig 2**. (Continued).


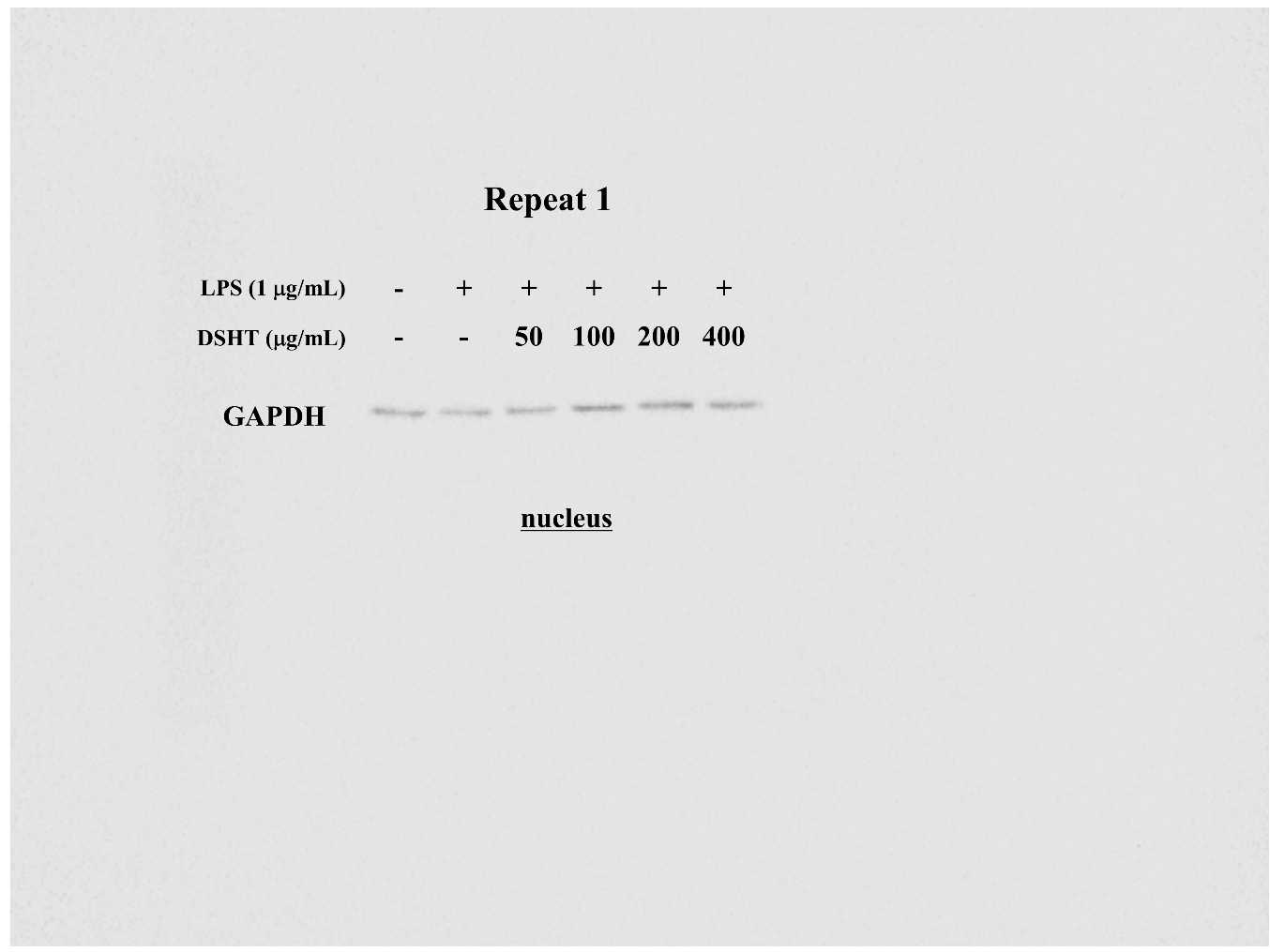


**Supplementary Fig 2**. (Continued).


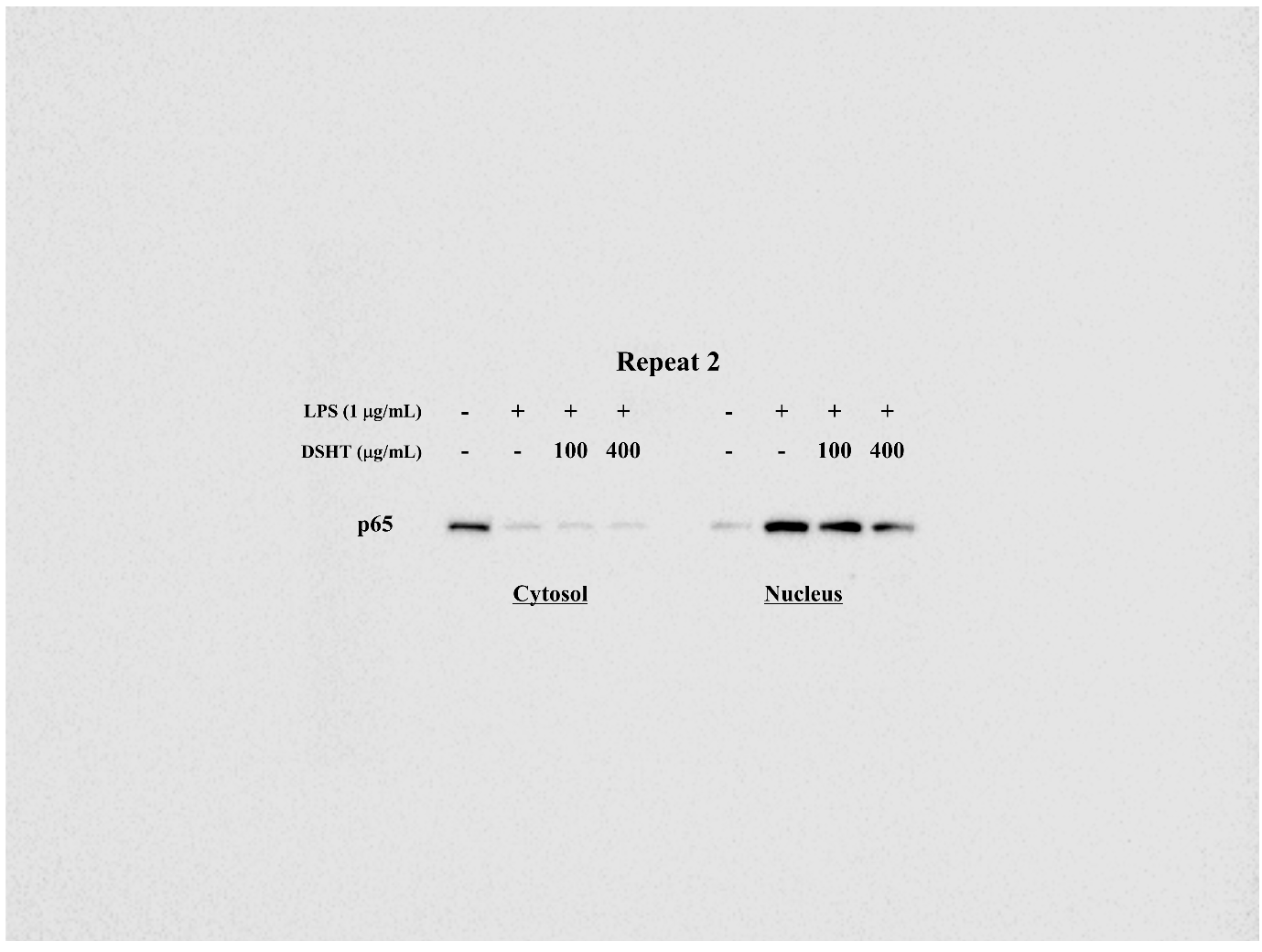


**Supplementary Fig 2**. (Continued).


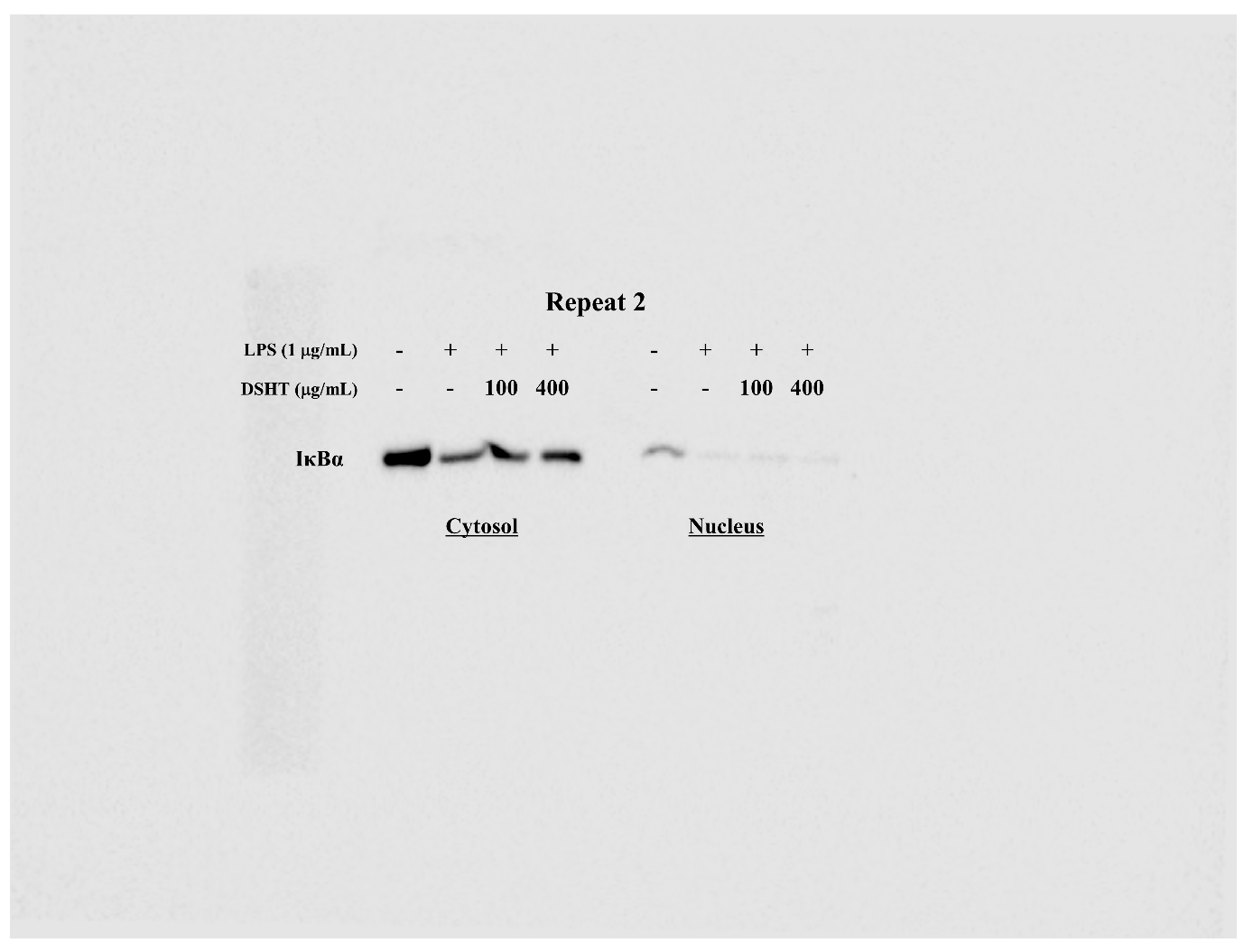
**Supplementary Fig 2**. (Continued).


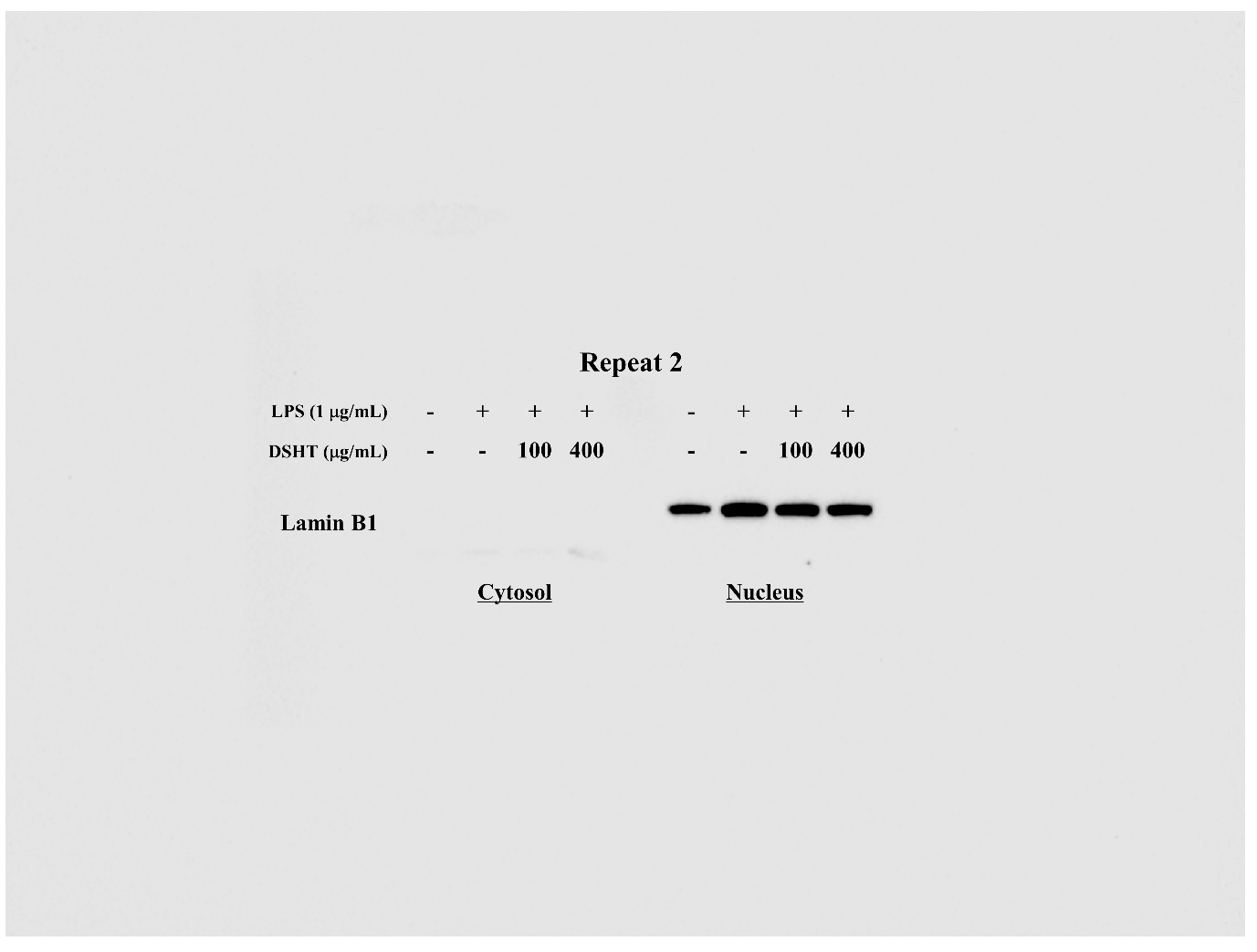


**Supplementary Fig 2**. (Continued).


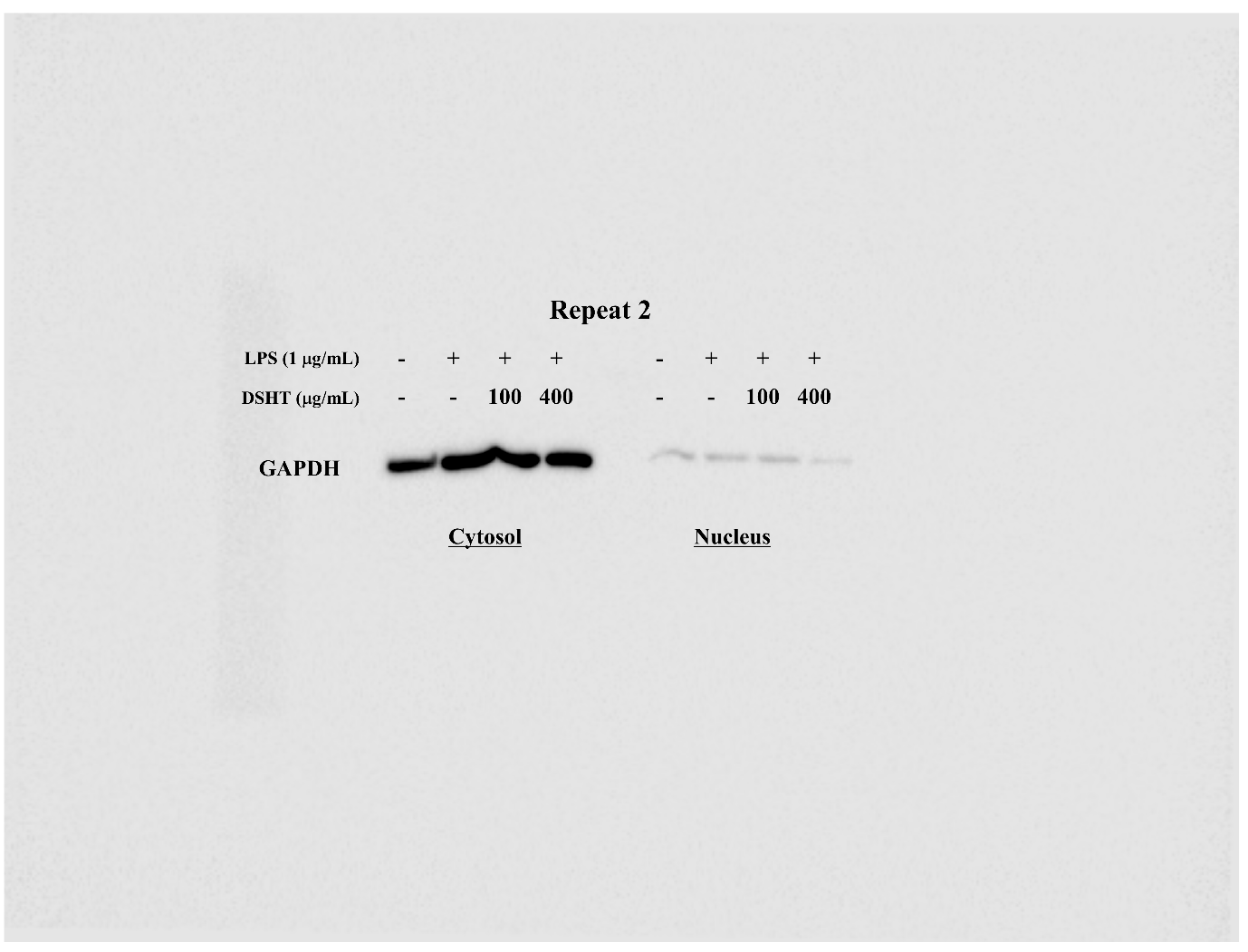


**Supplementary Fig 2**. (Continued).


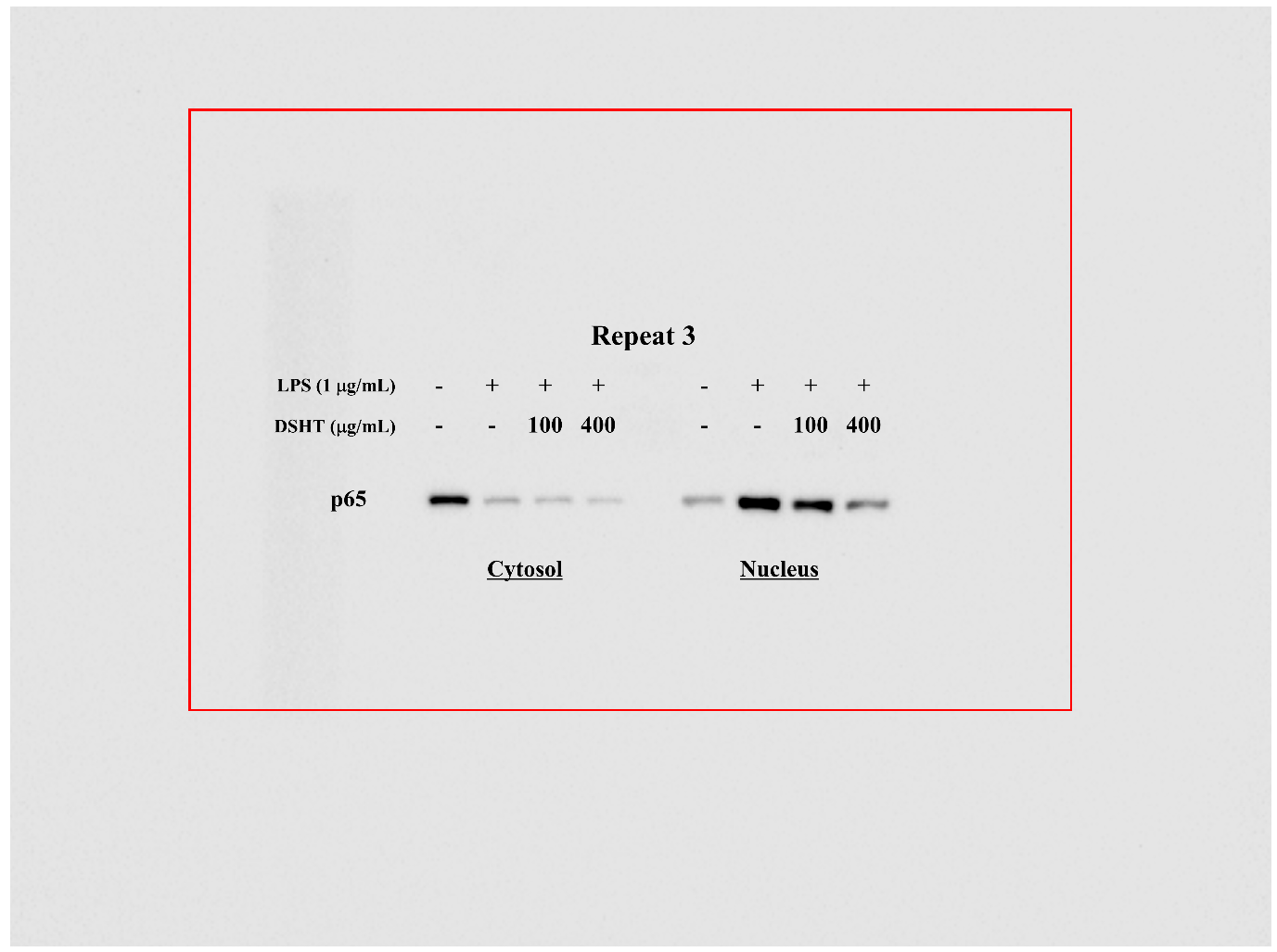


**Supplementary Fig 2**. (Continued).


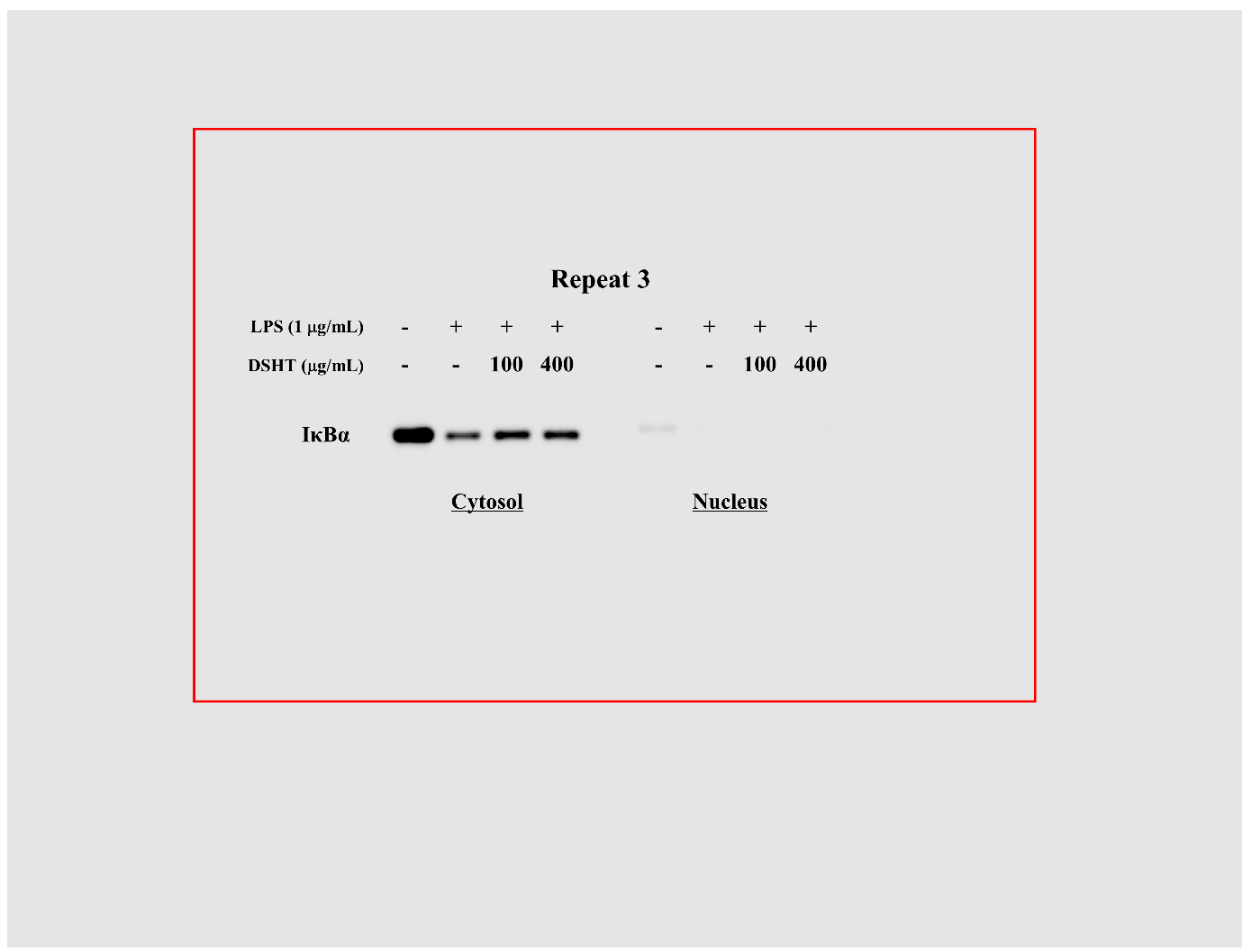


**Supplementary Fig 2**. (Continued).


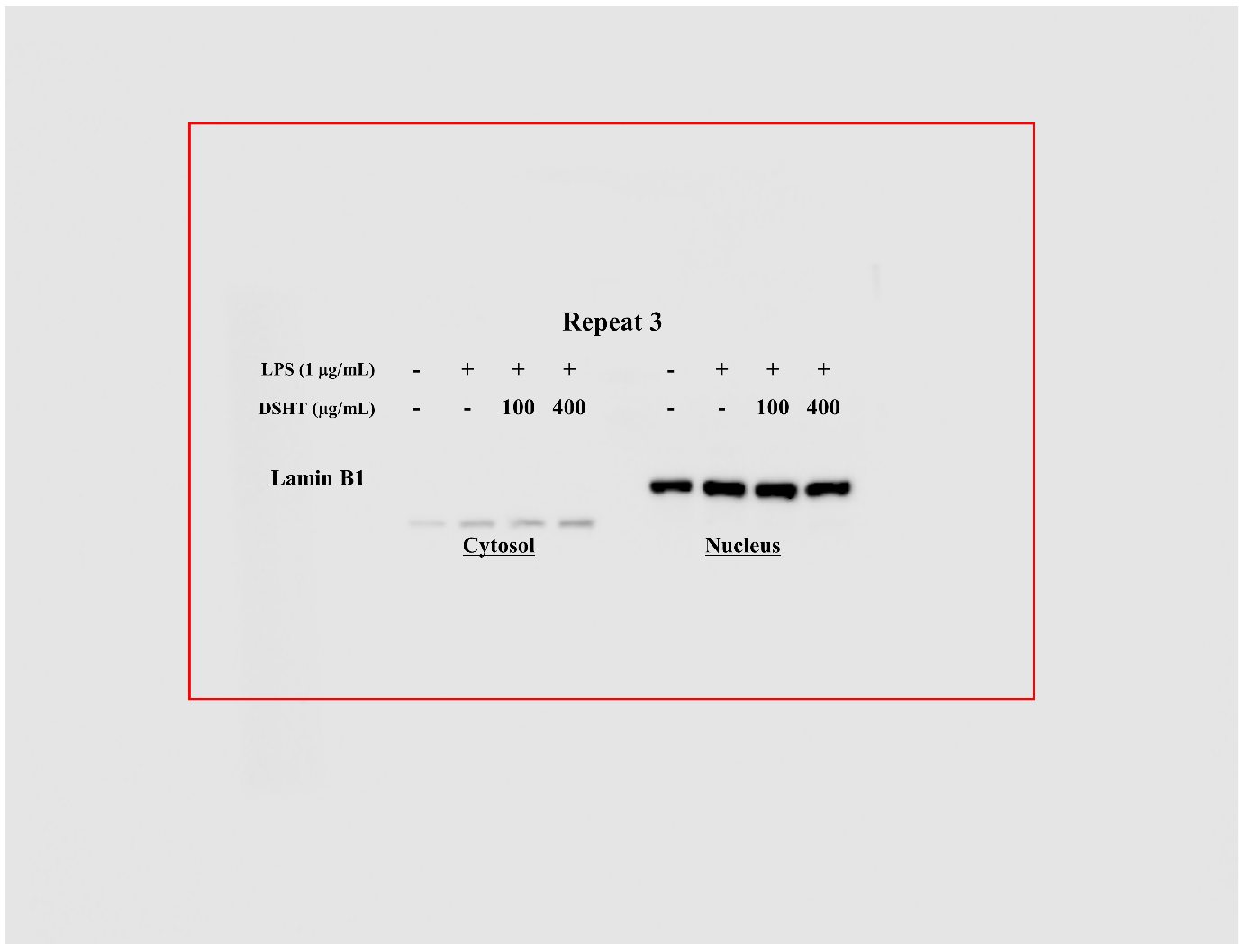


**Supplementary Fig 2**. (Continued).


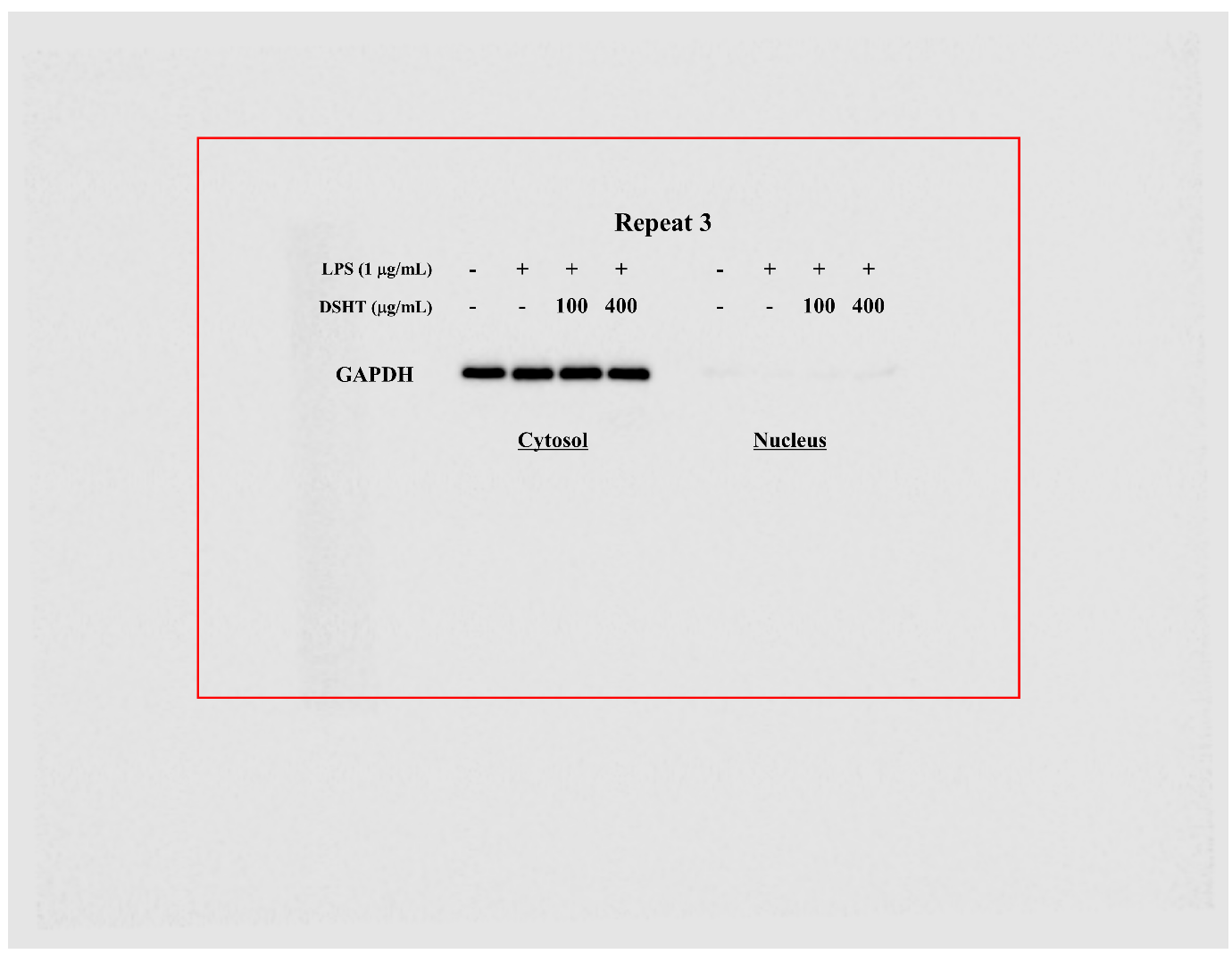


**Supplementary Fig 2**. (Continued).

**
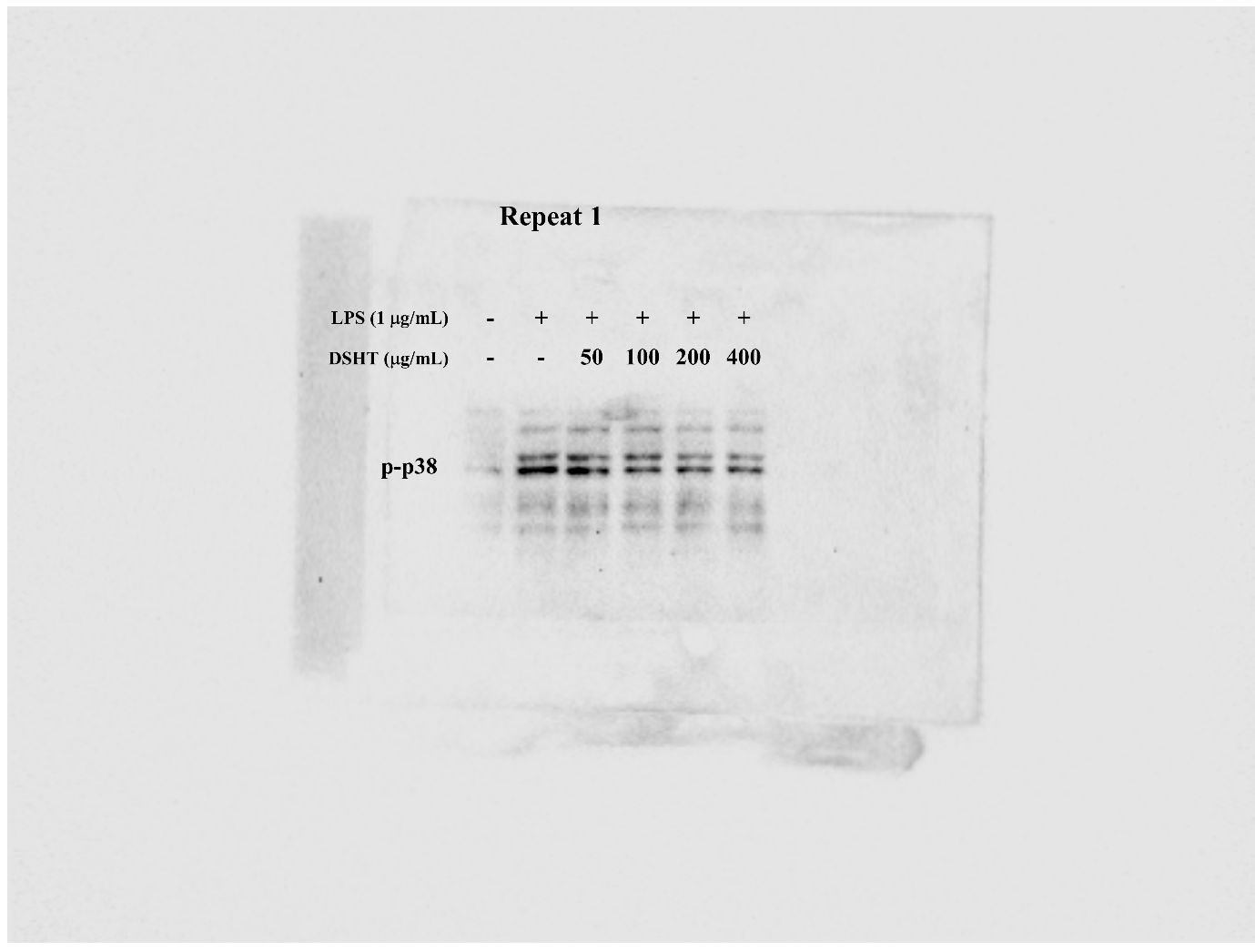
**

**Supplementary Fig 3**. Full-length western blots for p-p38, p38, p-ERK, ERK, p-JNK, and JNK for three repeats. Blot was incubated with specific antibodies for p-p38, p-ERK, and p-JNK, respectively. After that each membrane was stripped before being incubated with specific antibodies for p38, ERK, and JNK, respectively. Main figures are displayed using red box. Western blot shown in Fig 5.


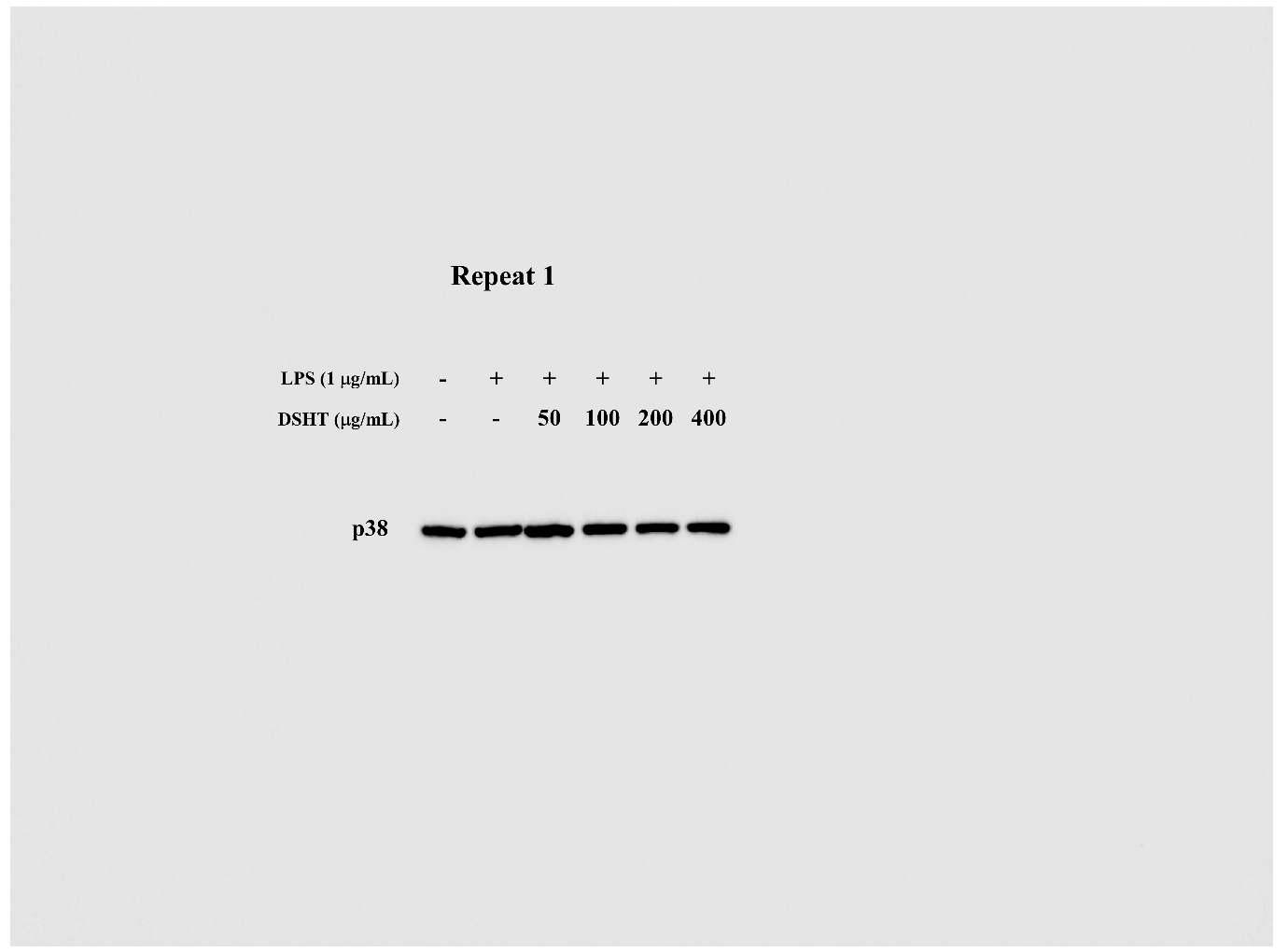


**Supplementary Fig 3**. (Continued).

**
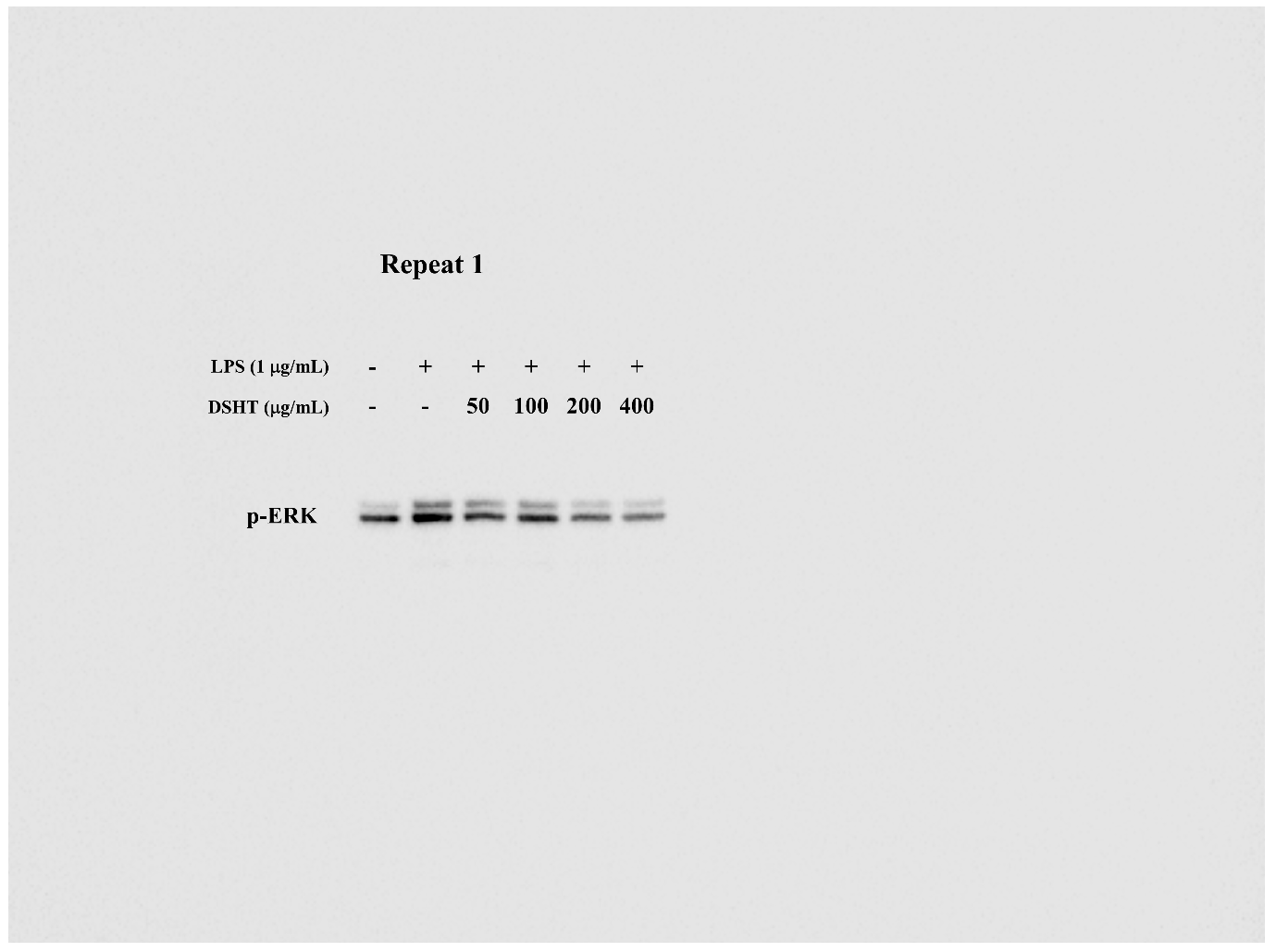
**

**Supplementary Fig 3**. (Continued).

**
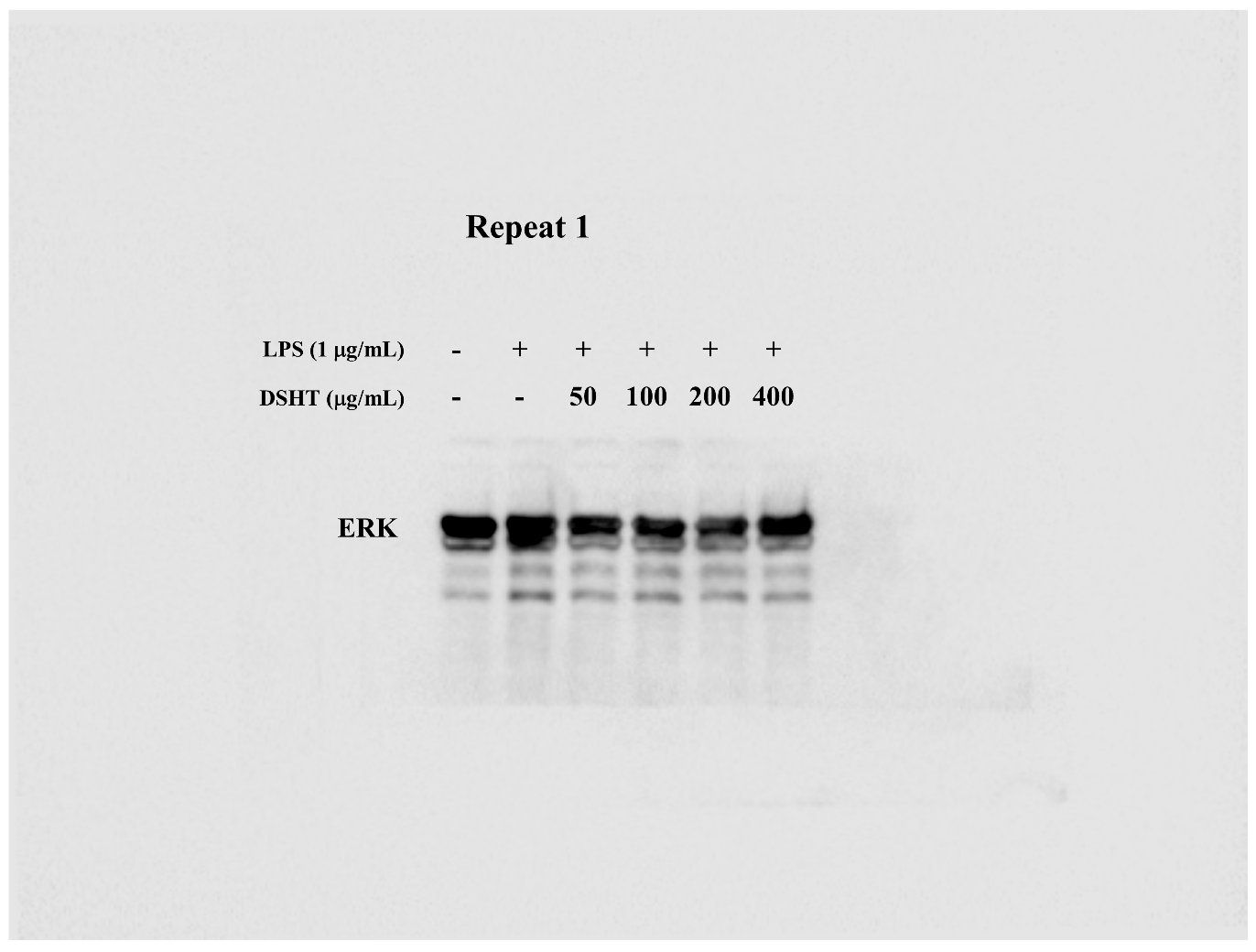
**

**Supplementary Fig 3**. (Continued).


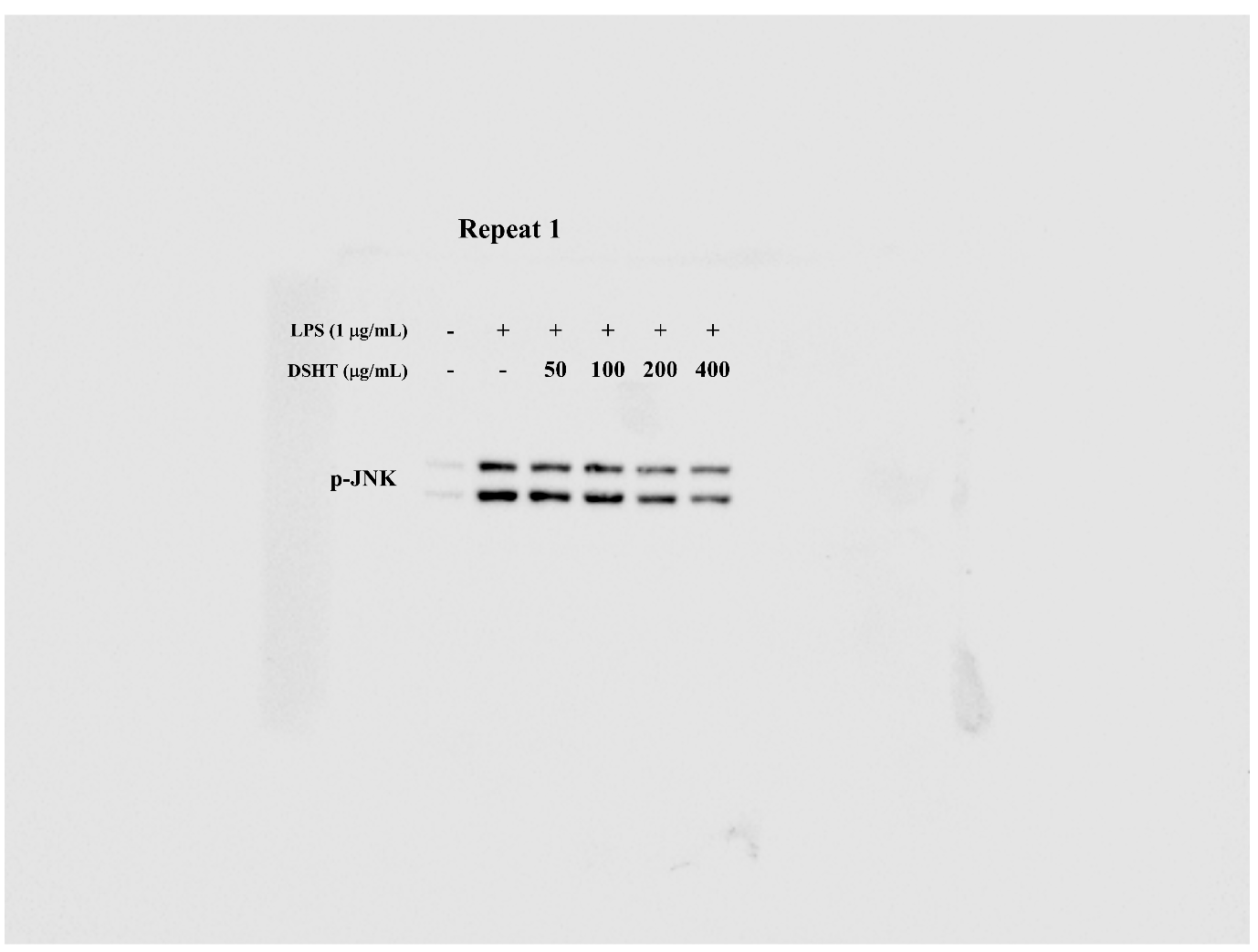


**Supplementary Fig 3**. (Continued).


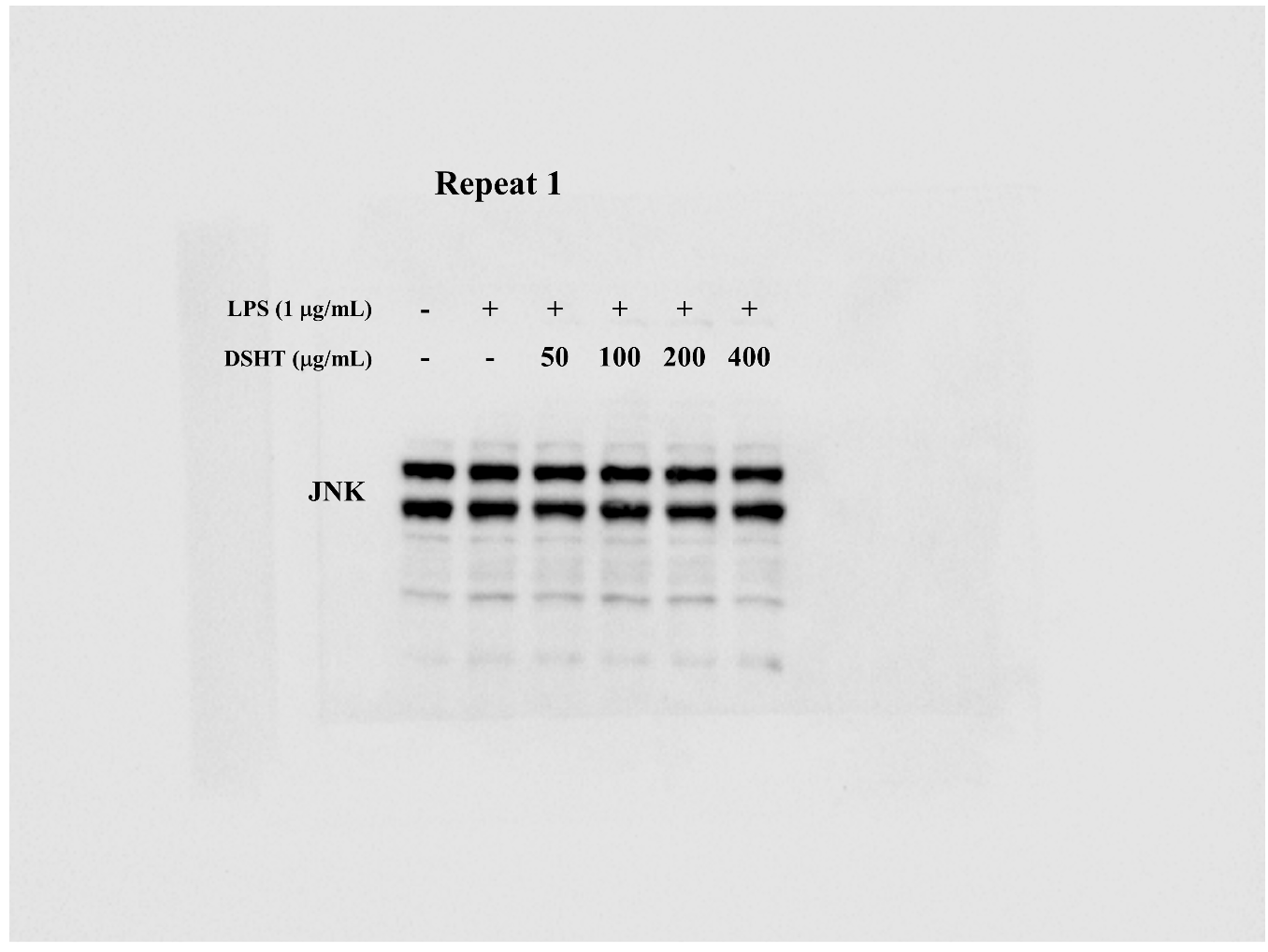


**Supplementary Fig 3**. (Continued).


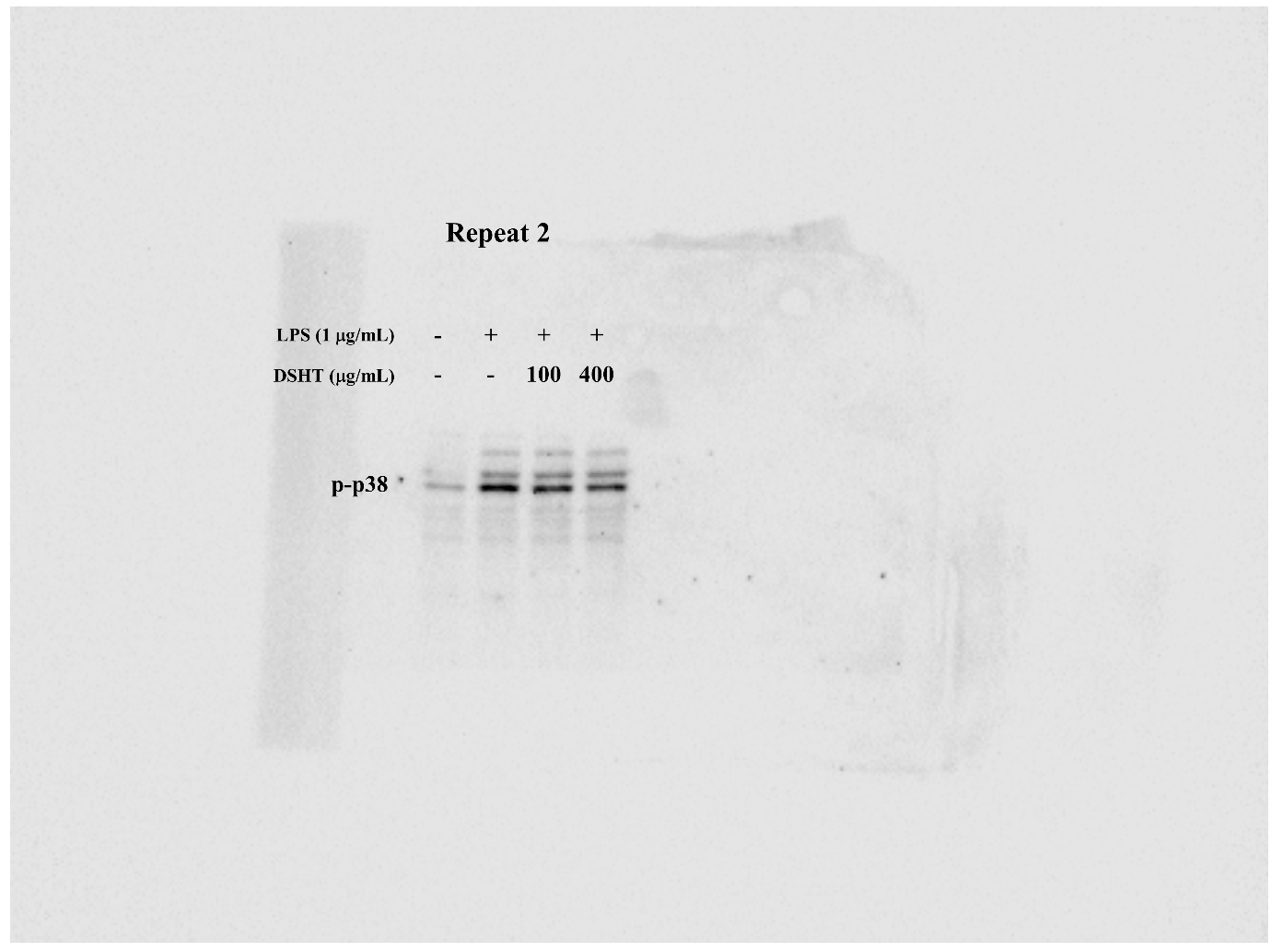


**Supplementary Fig 3**. (Continued).


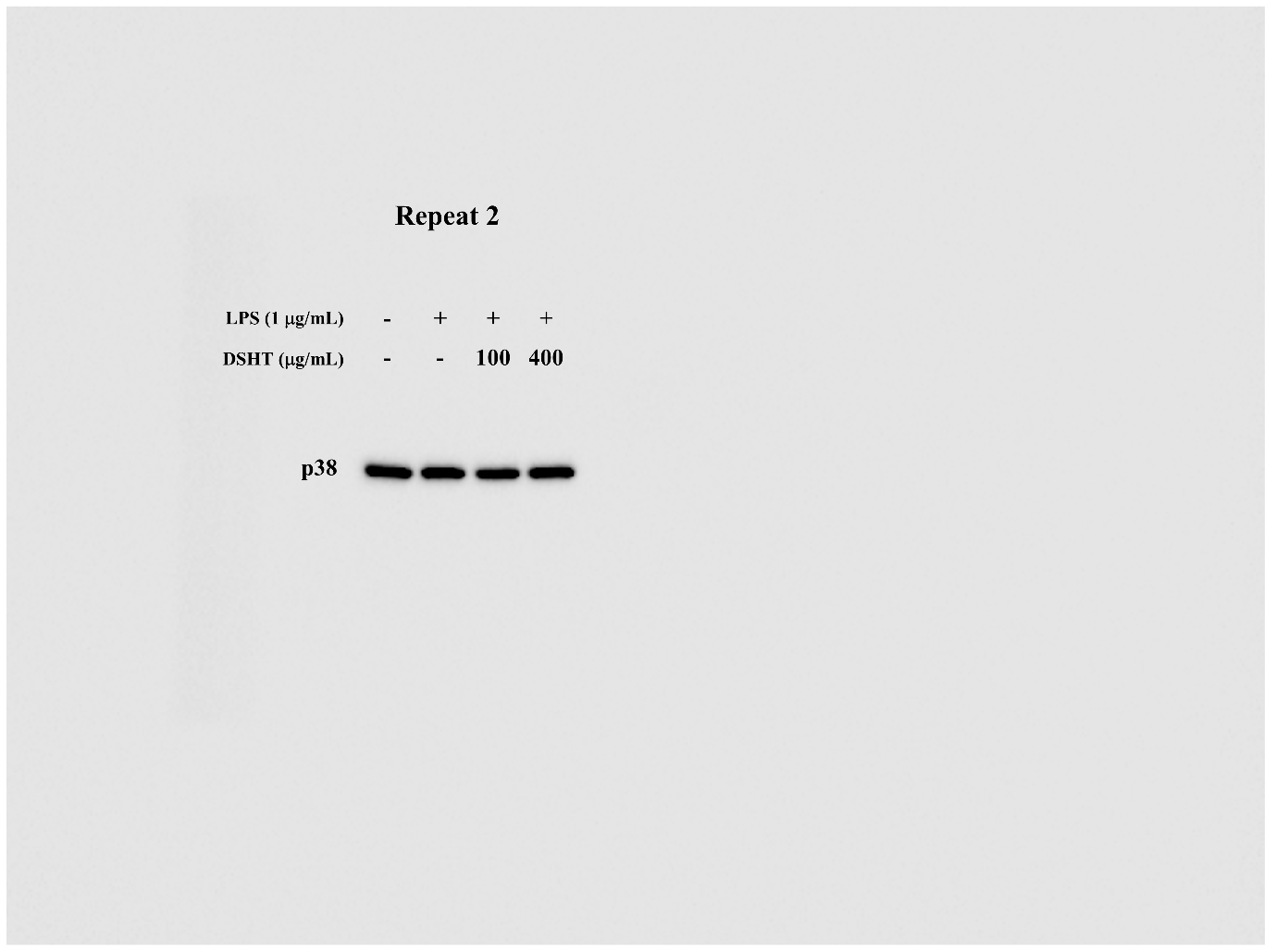


**Supplementary Fig 3**. (Continued).


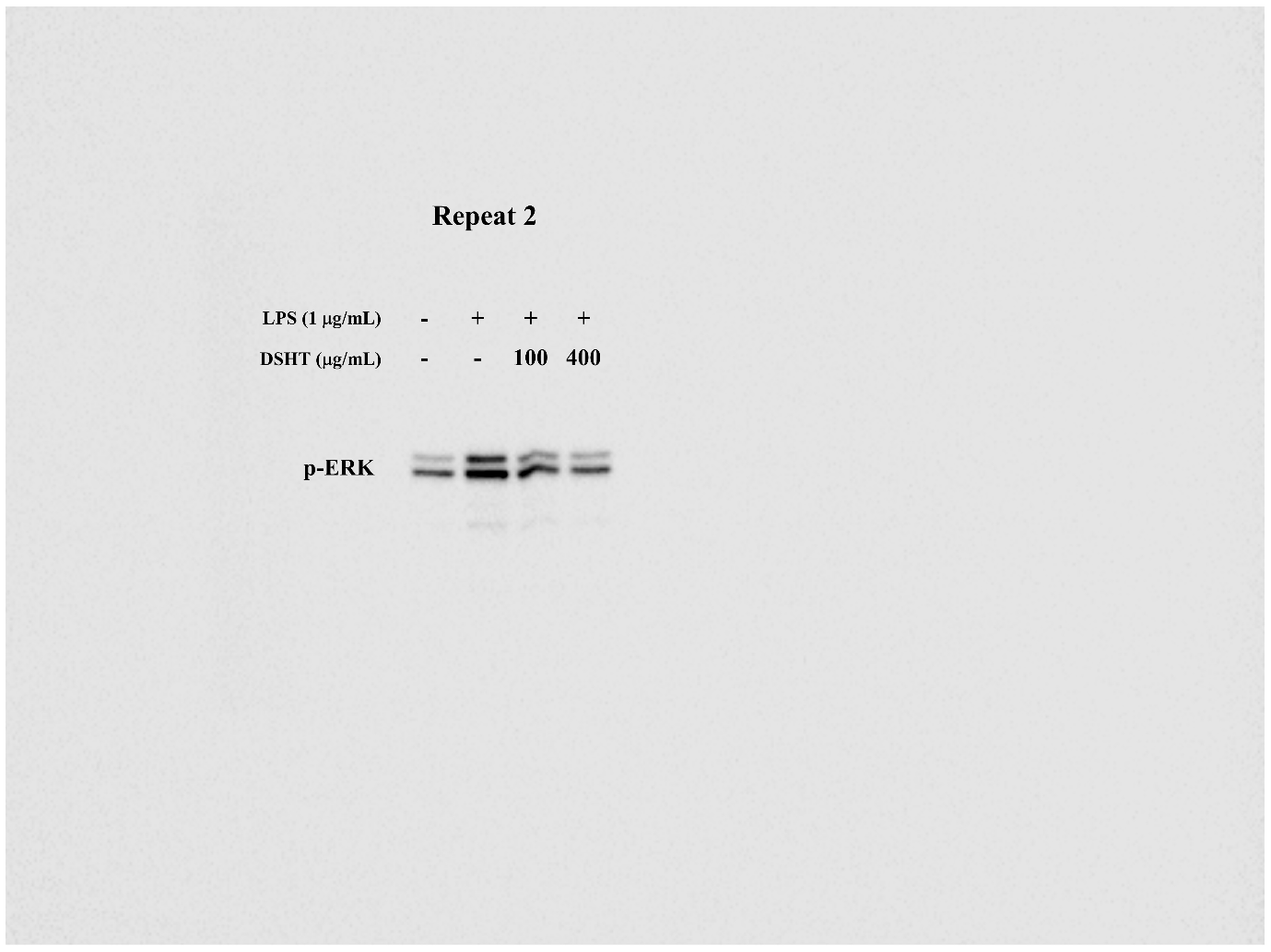


**Supplementary Fig 3**. (Continued).


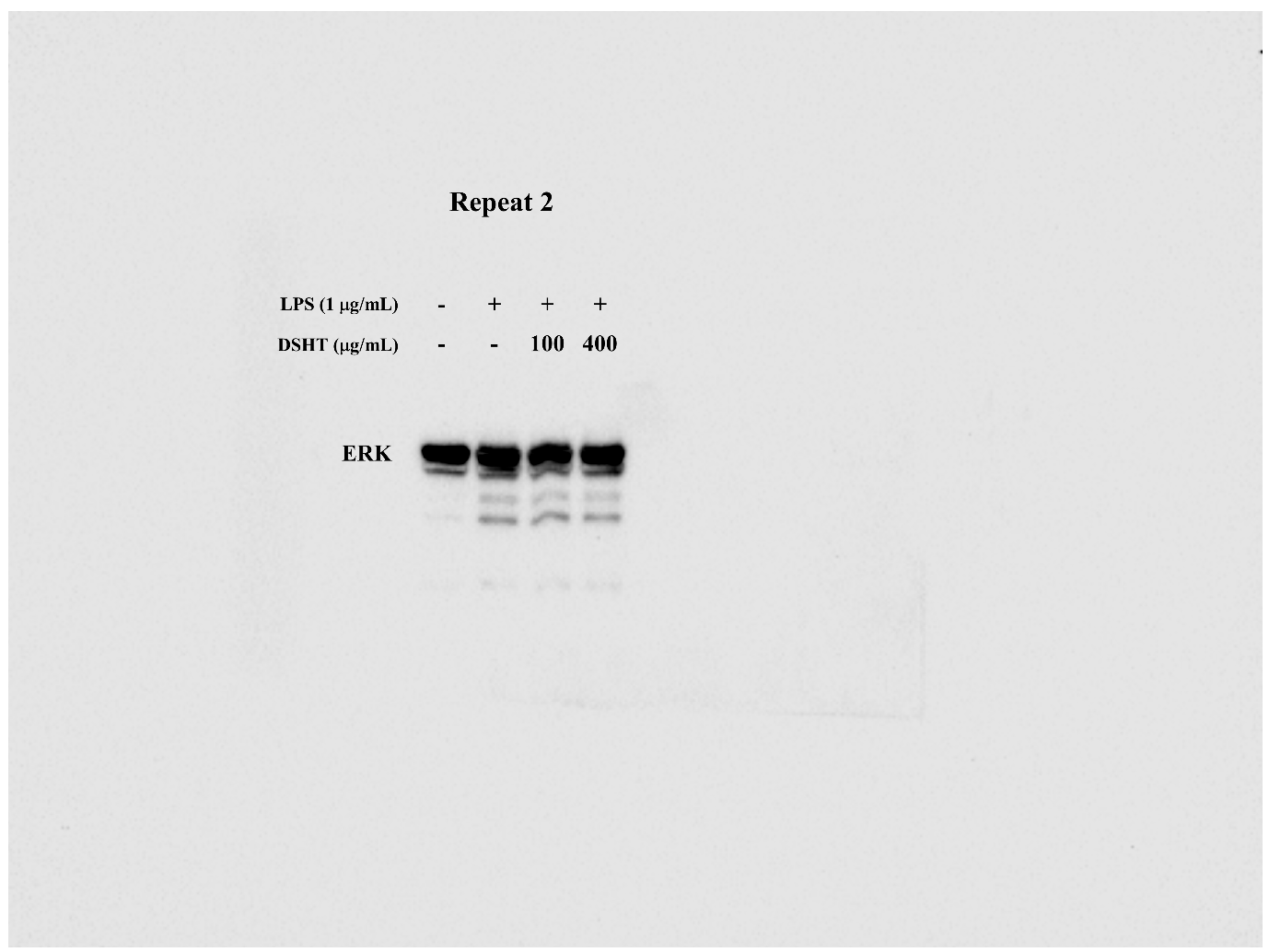


**Supplementary Fig 3**. (Continued).


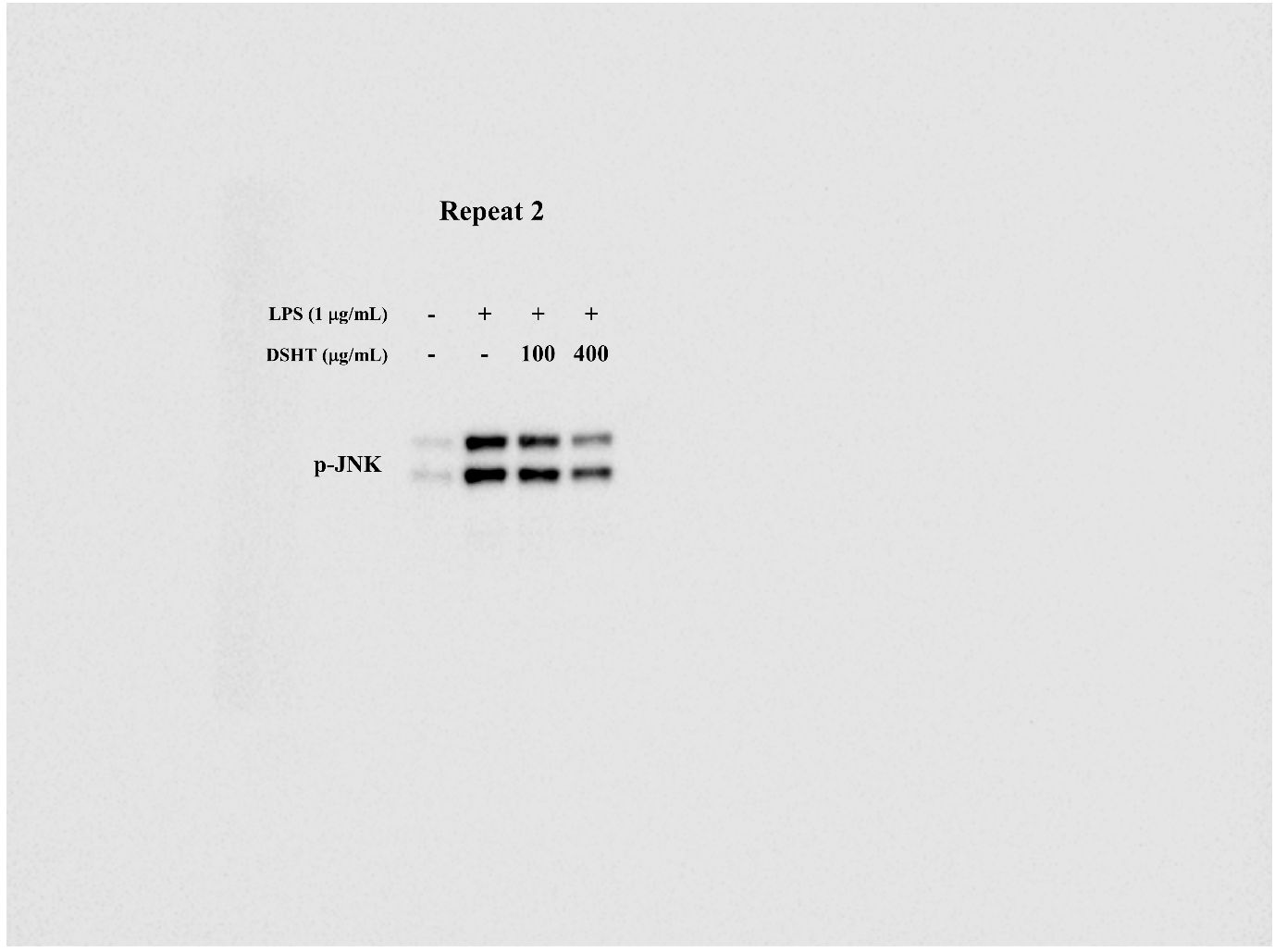


**Supplementary Fig 3**. (Continued).


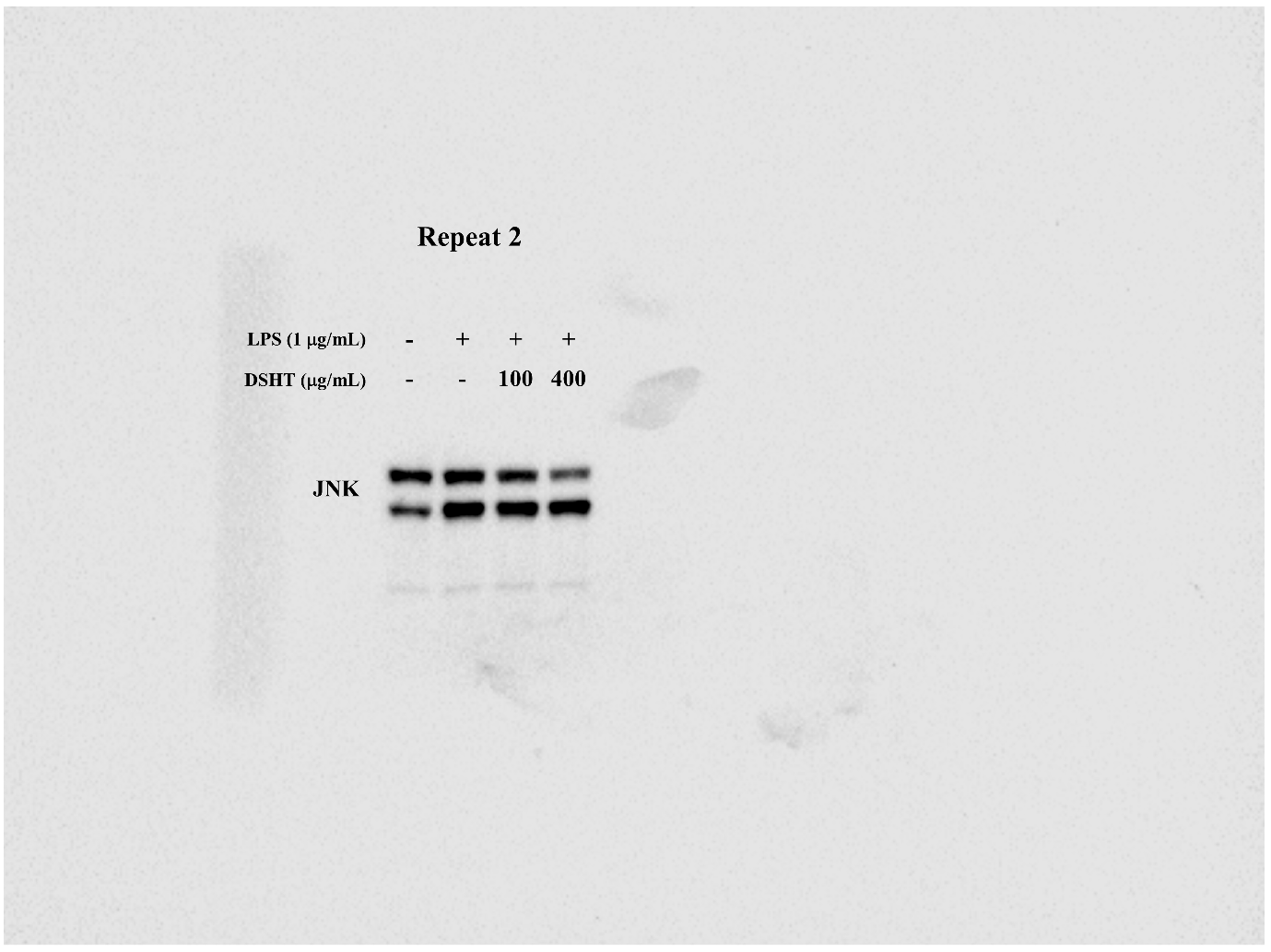


**Supplementary Fig 3**. (Continued).


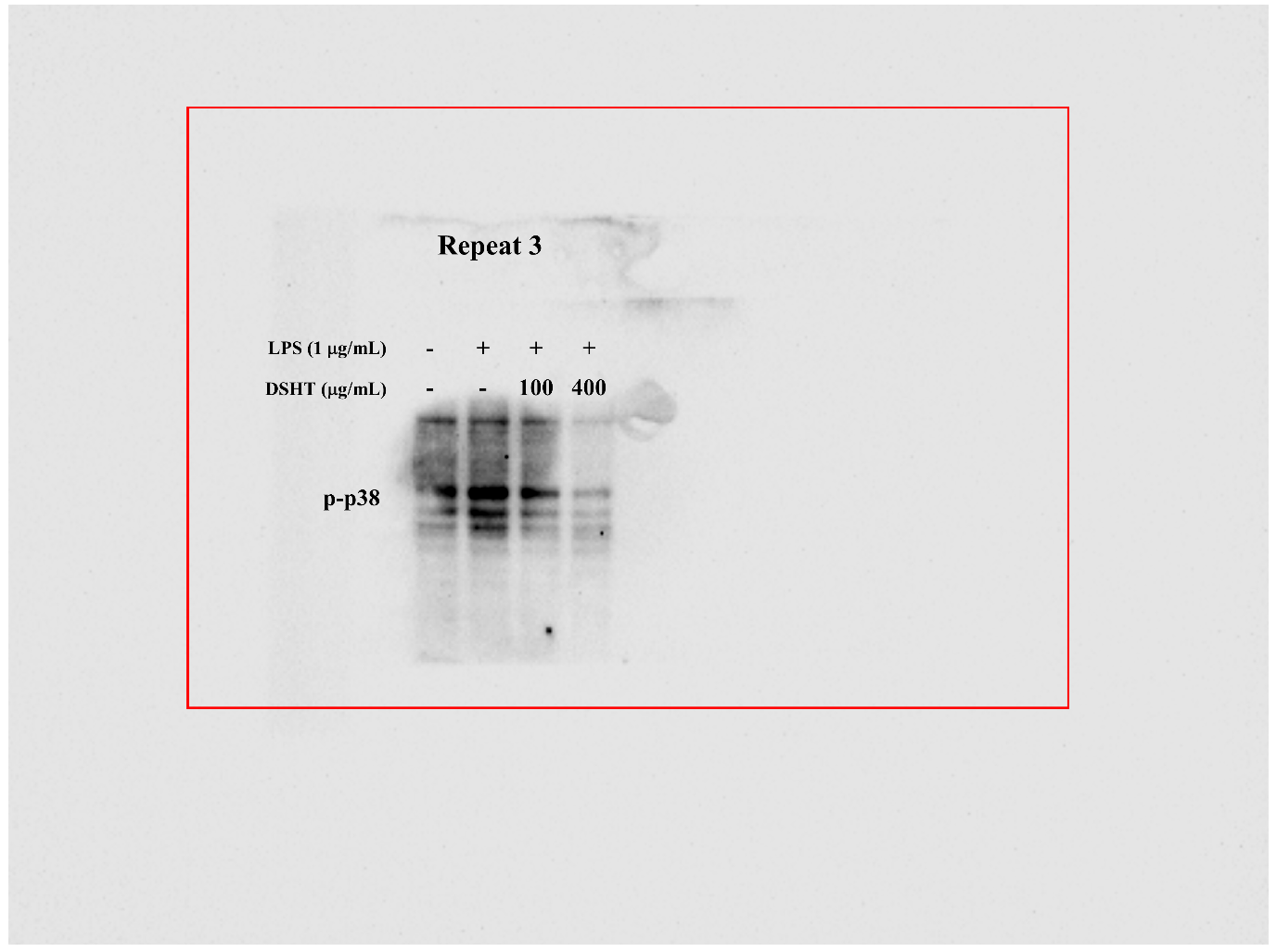


**Supplementary Fig 3**. (Continued).


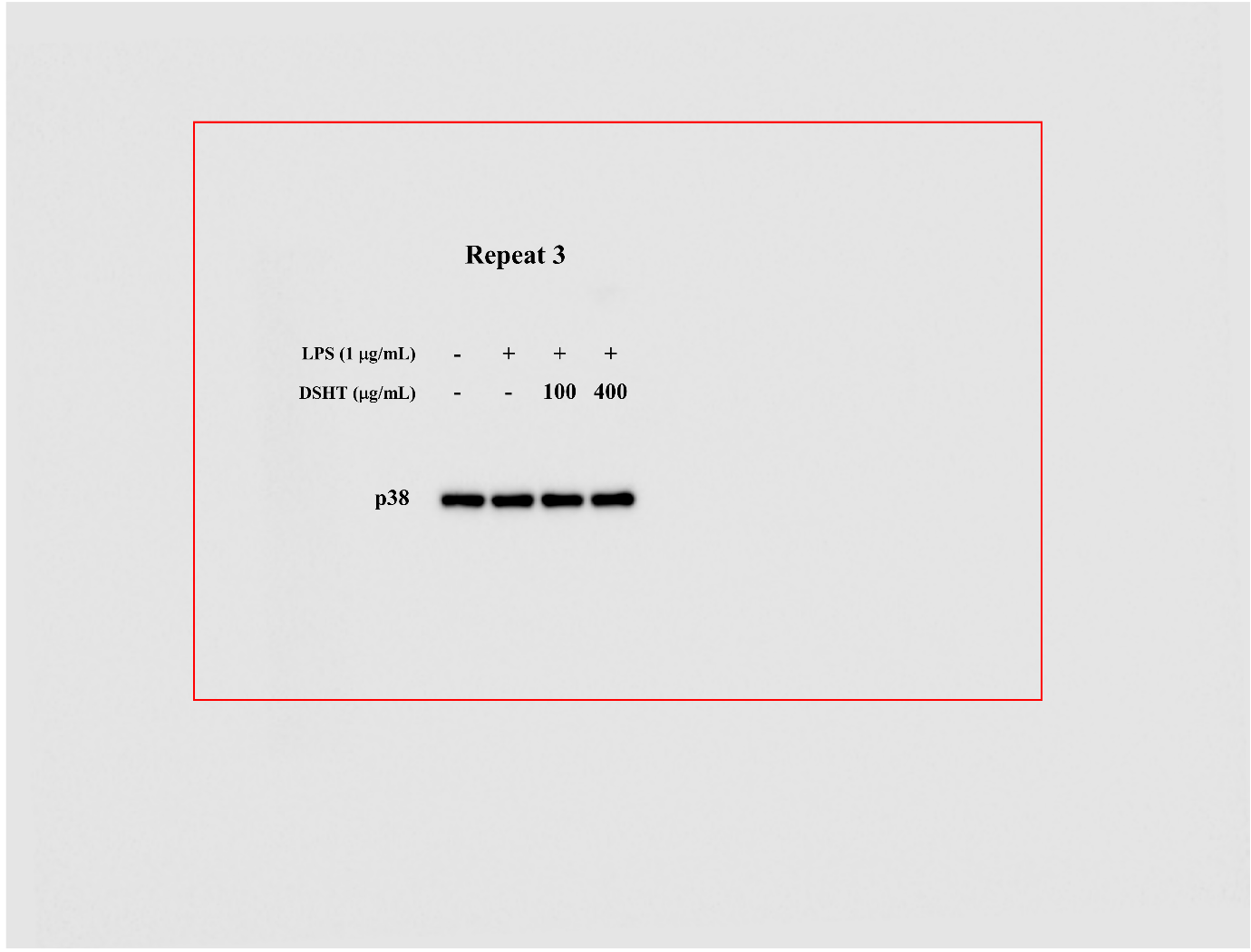


**Supplementary Fig 3**. (Continued).


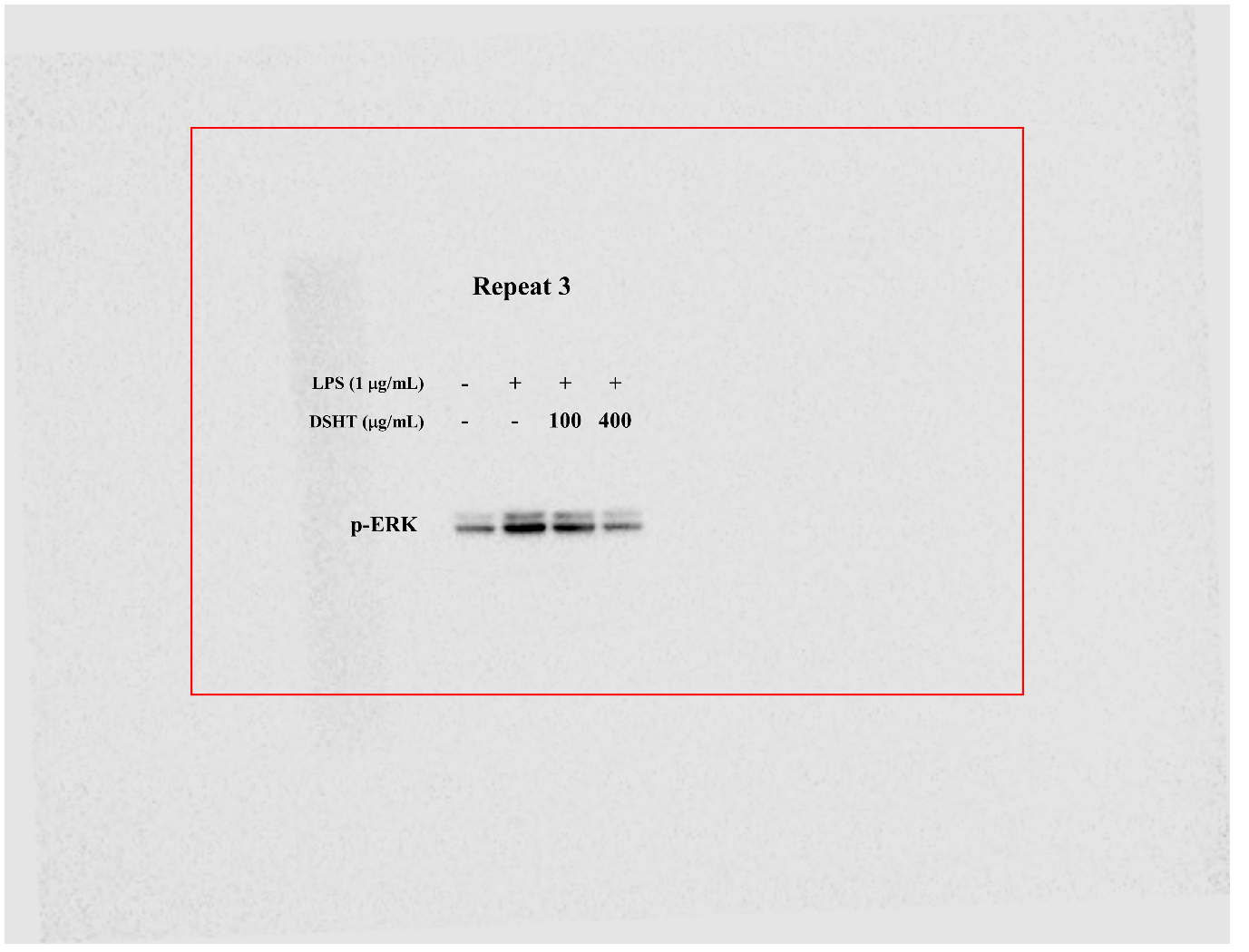


**Supplementary Fig 3**. (Continued).


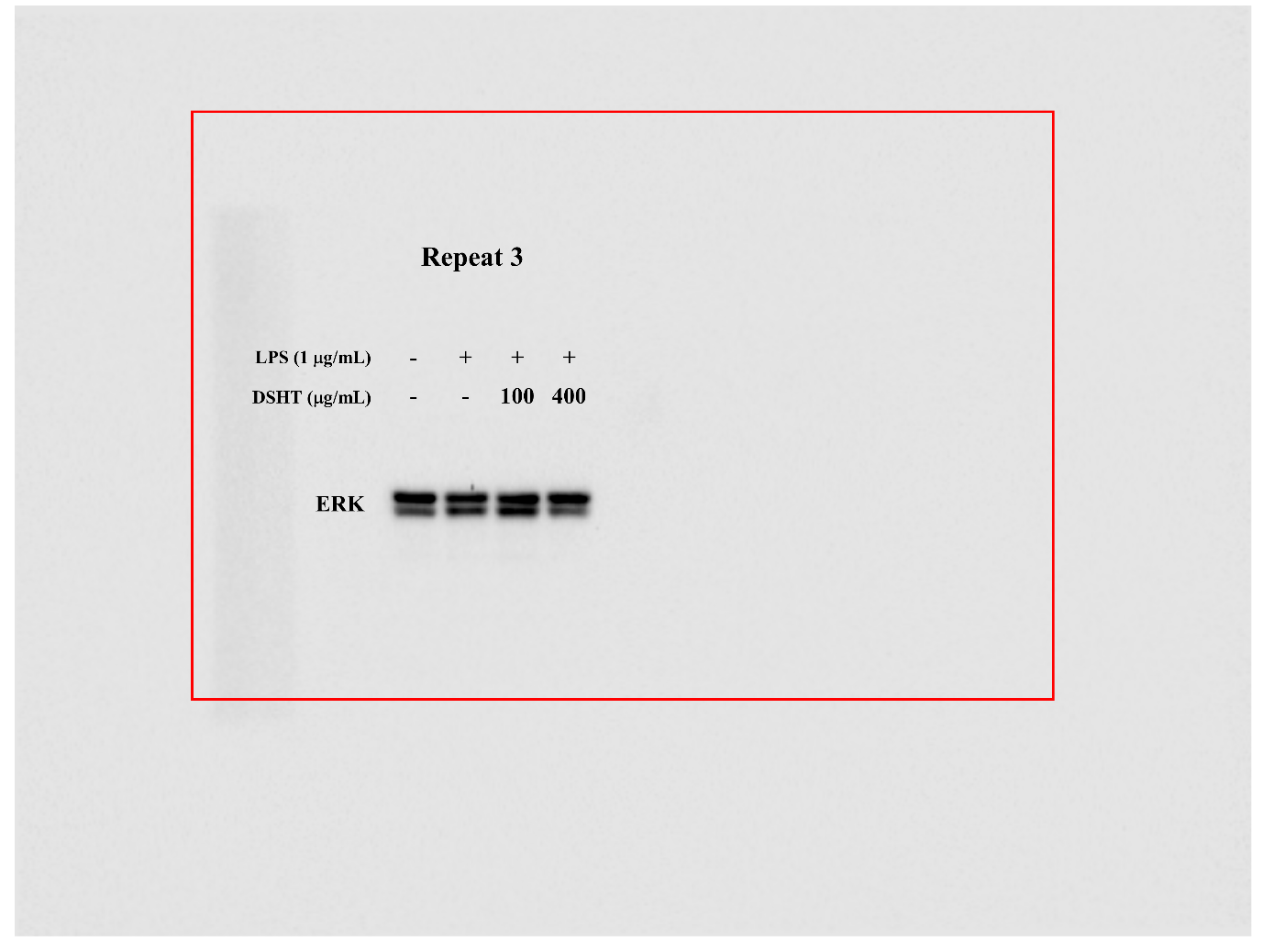


**Supplementary Fig 3**. (Continued).


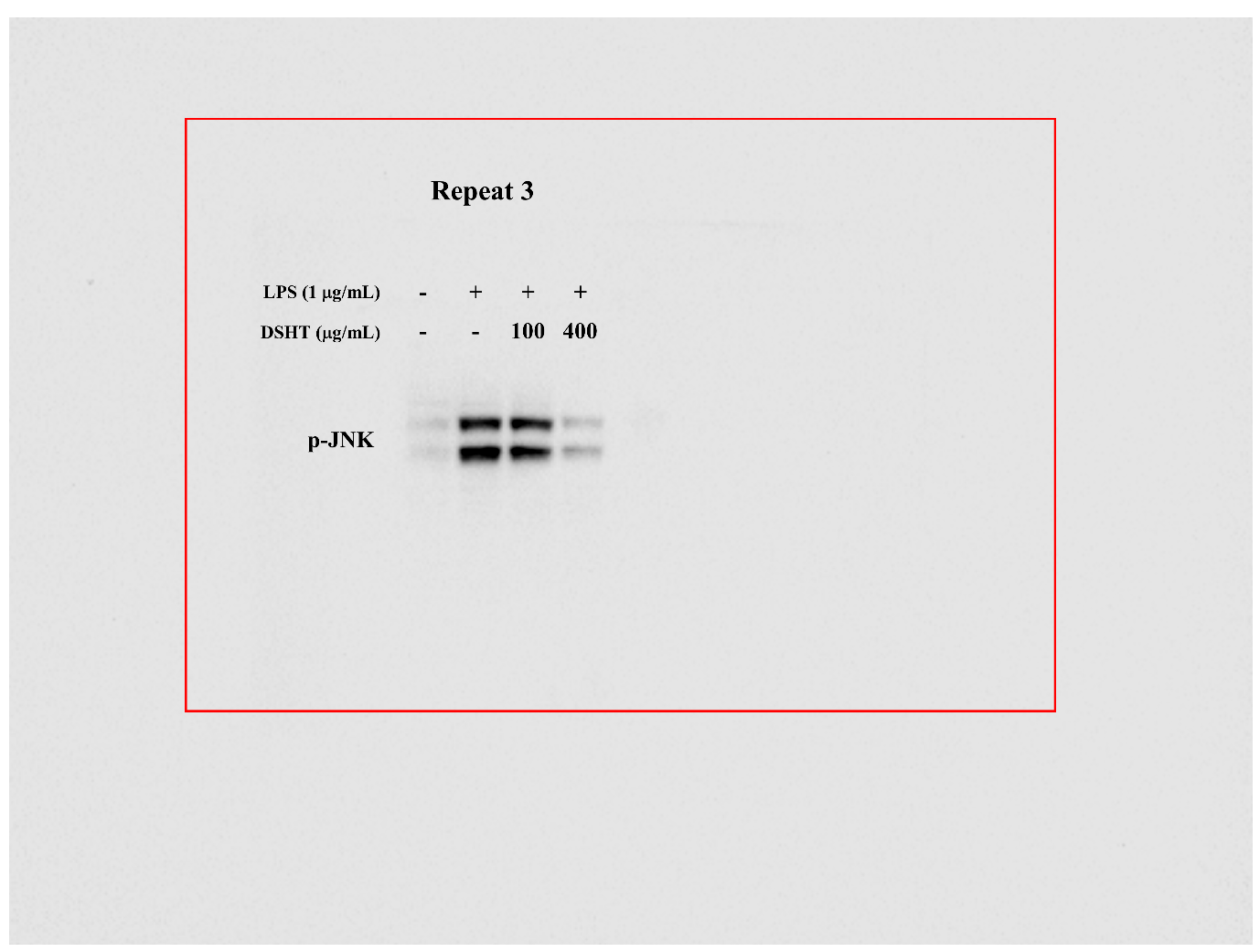


**Supplementary Fig 3**. (Continued).


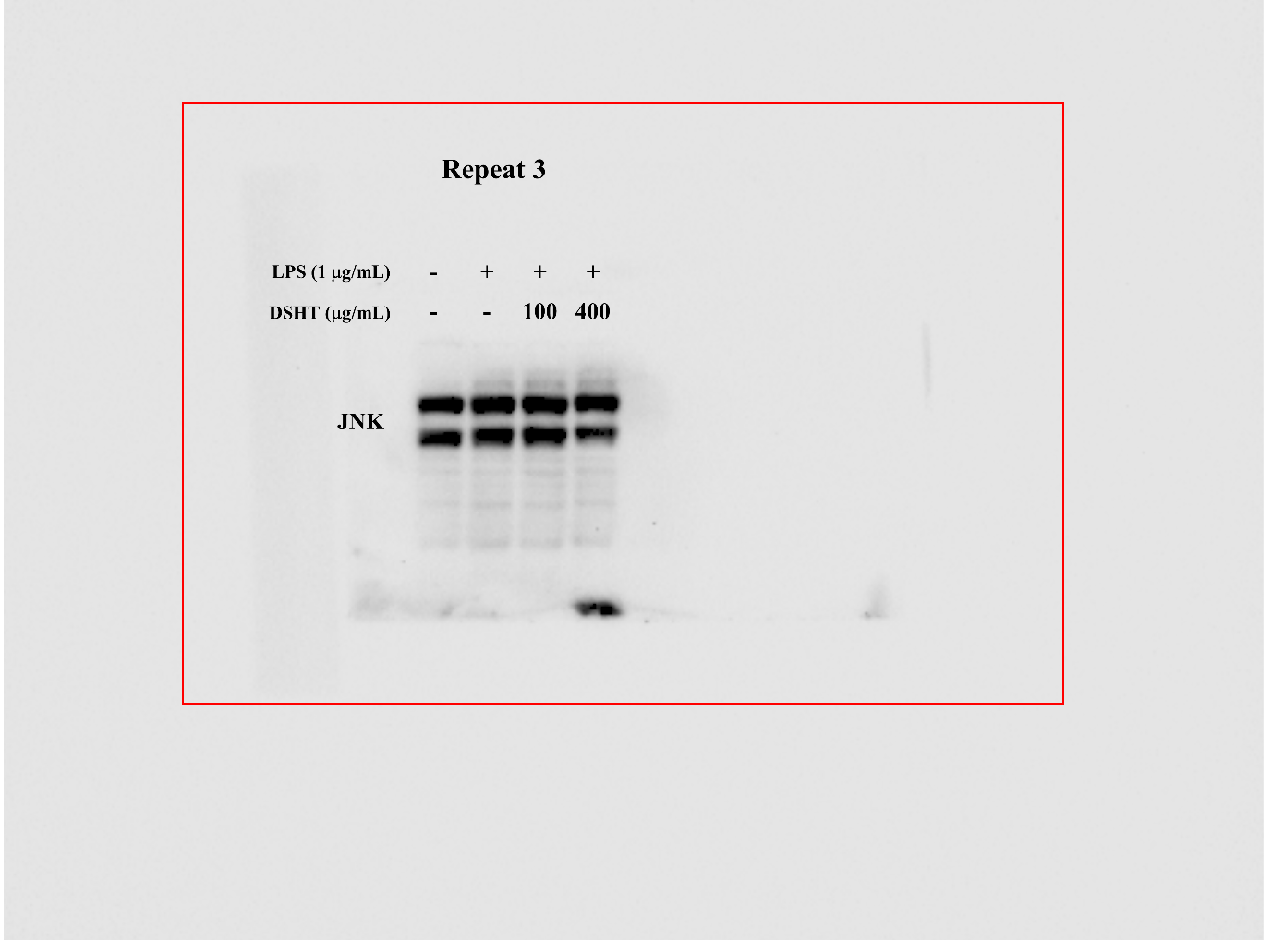


**Supplementary Fig 3**. (Continued).


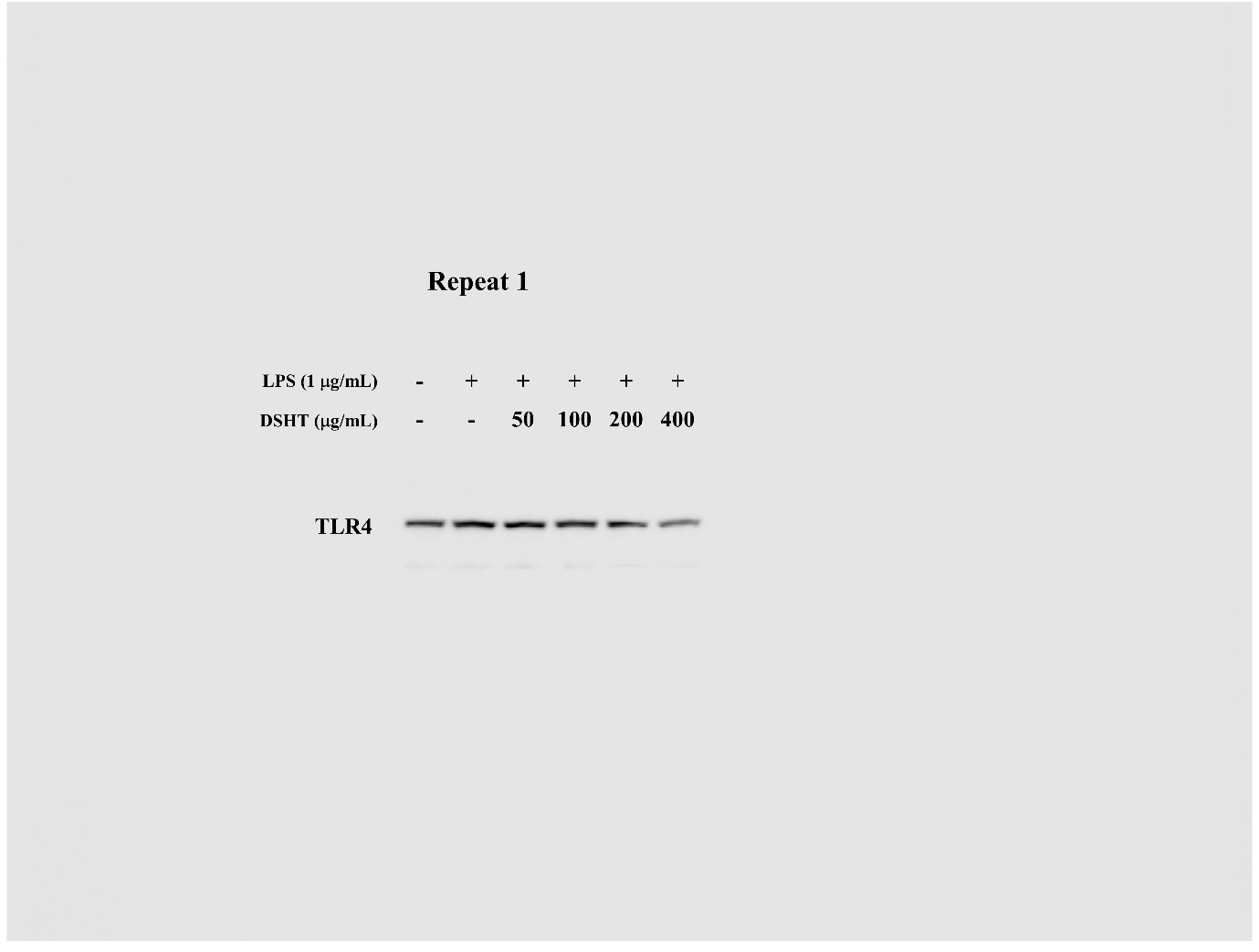


**Supplementary Fig 4**. Full-length western blots for TLR4, MyD88 and β-actin for three repeats. Blot was cut in three before being incubated with specific antibodies. Main figures are displayed using red box. Western blot used for Fig 6.


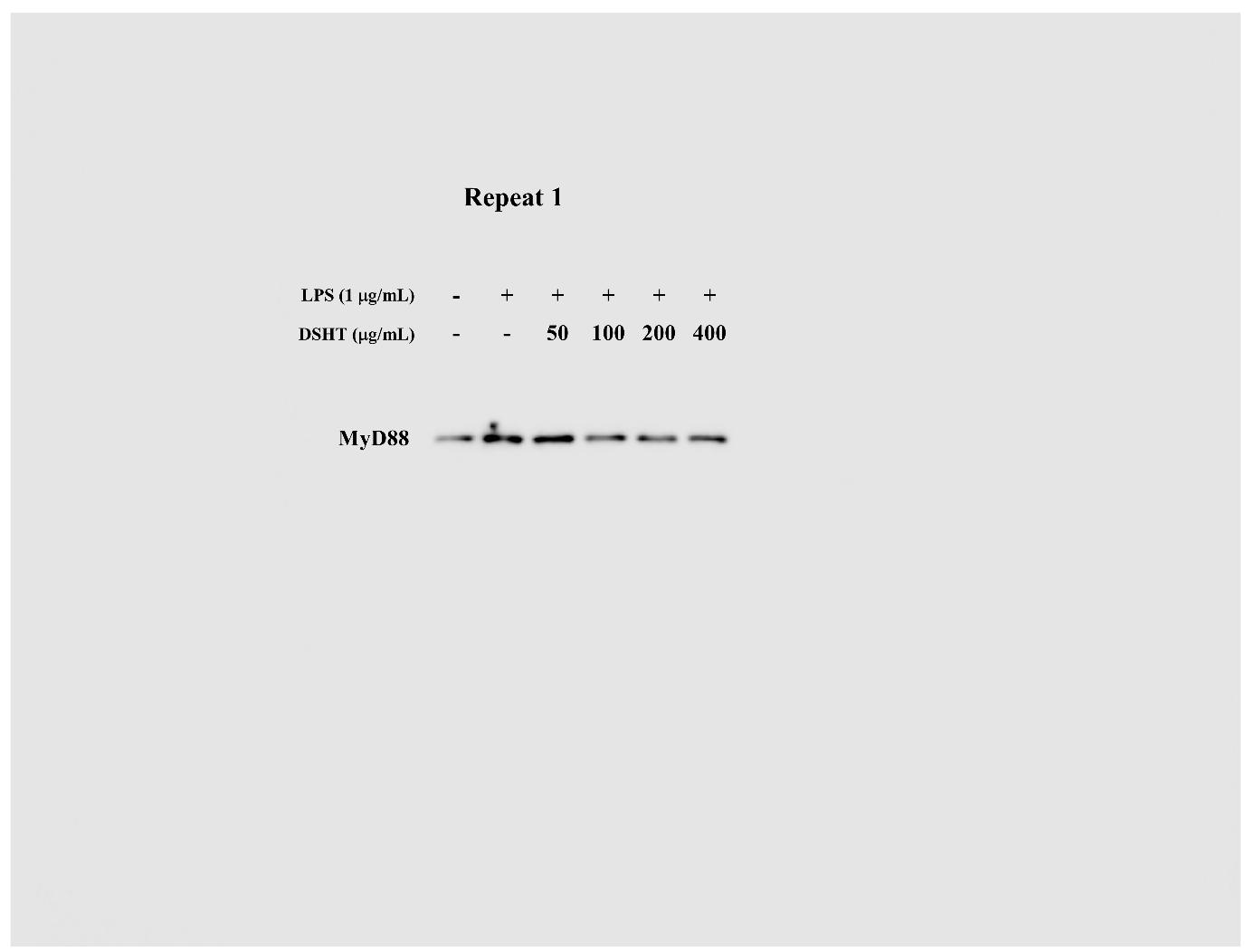


**Supplementary Fig 4**. (Continued).


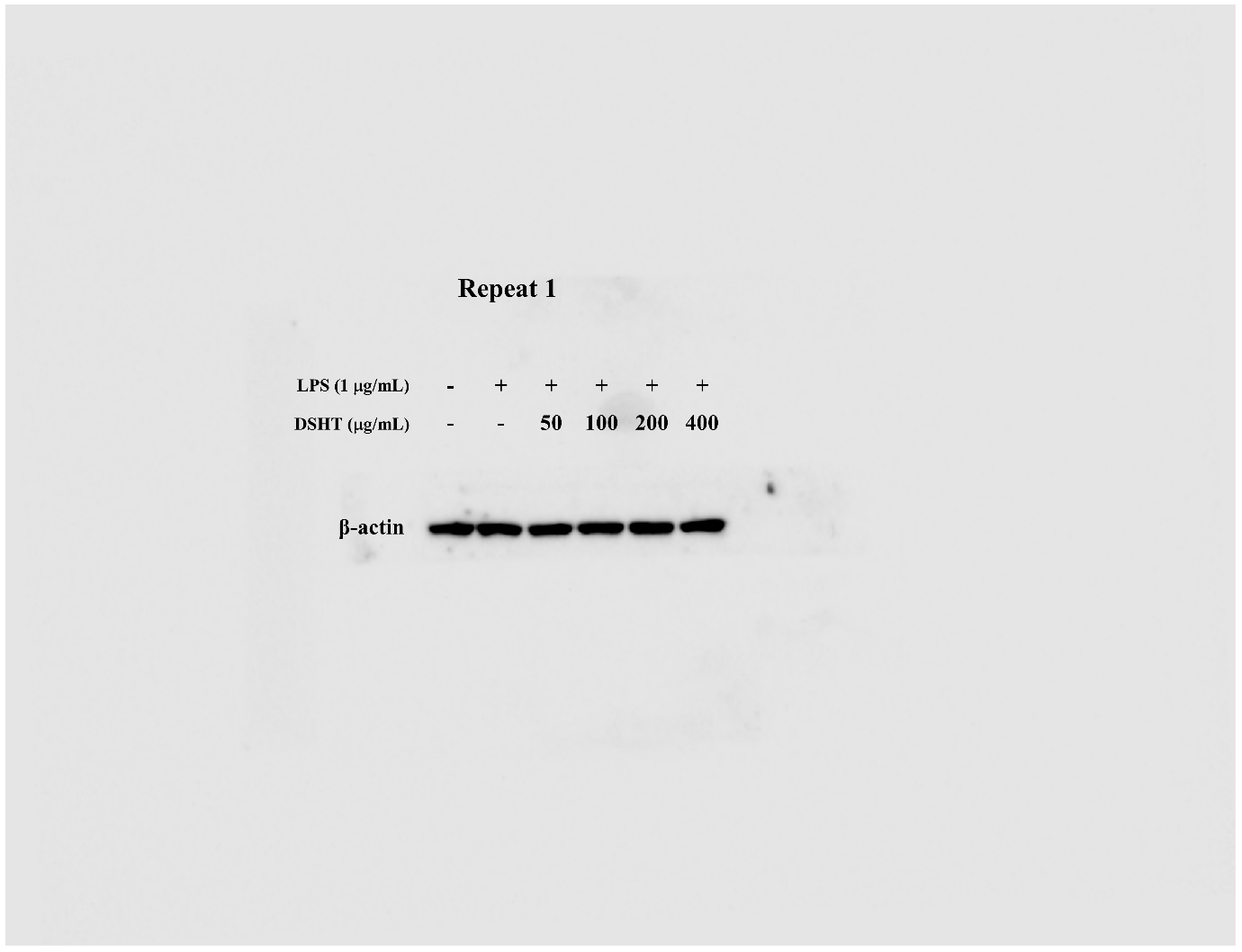


**Supplementary Fig 4**. (Continued).


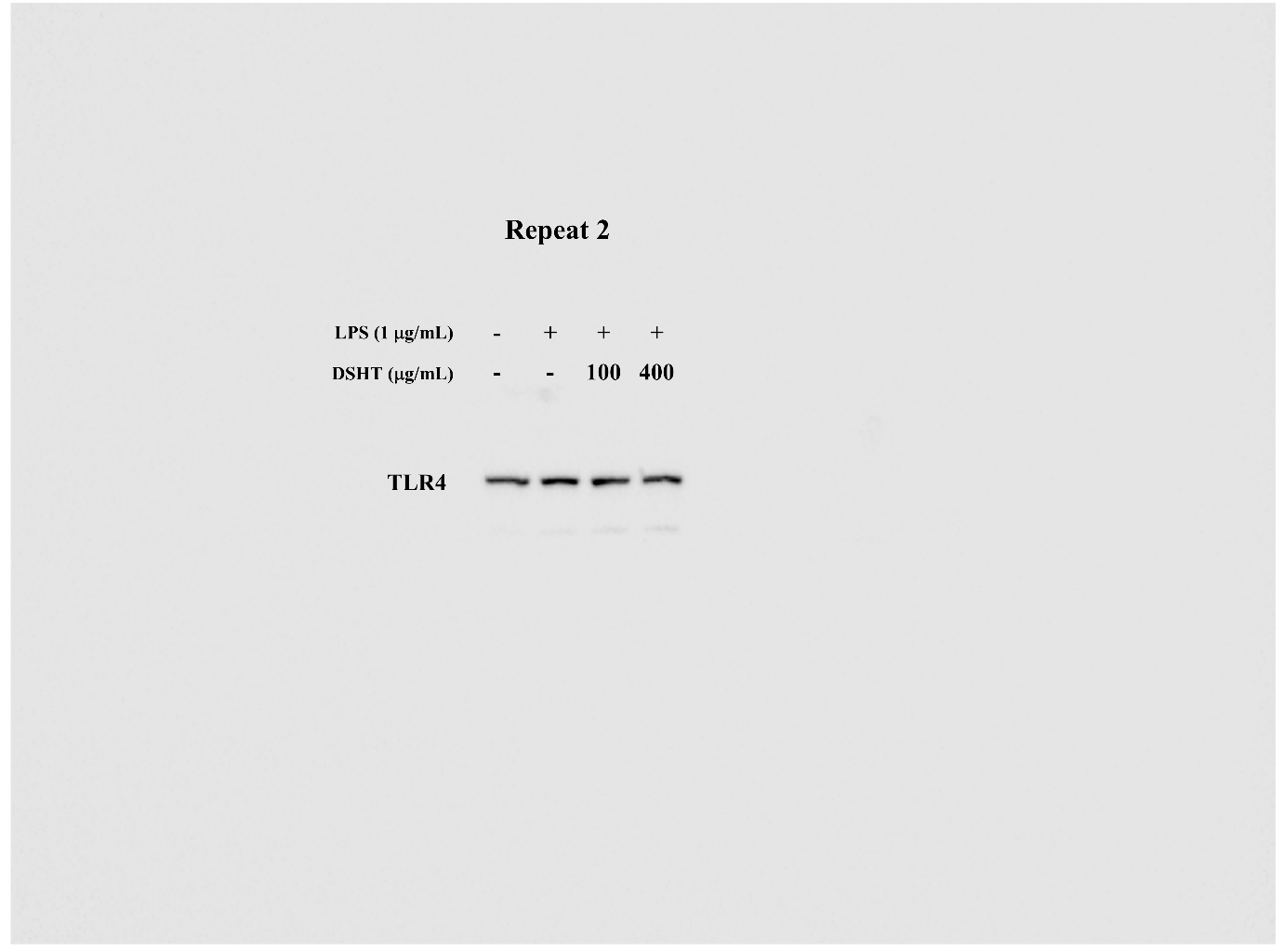


**Supplementary Fig 4**. (Continued).


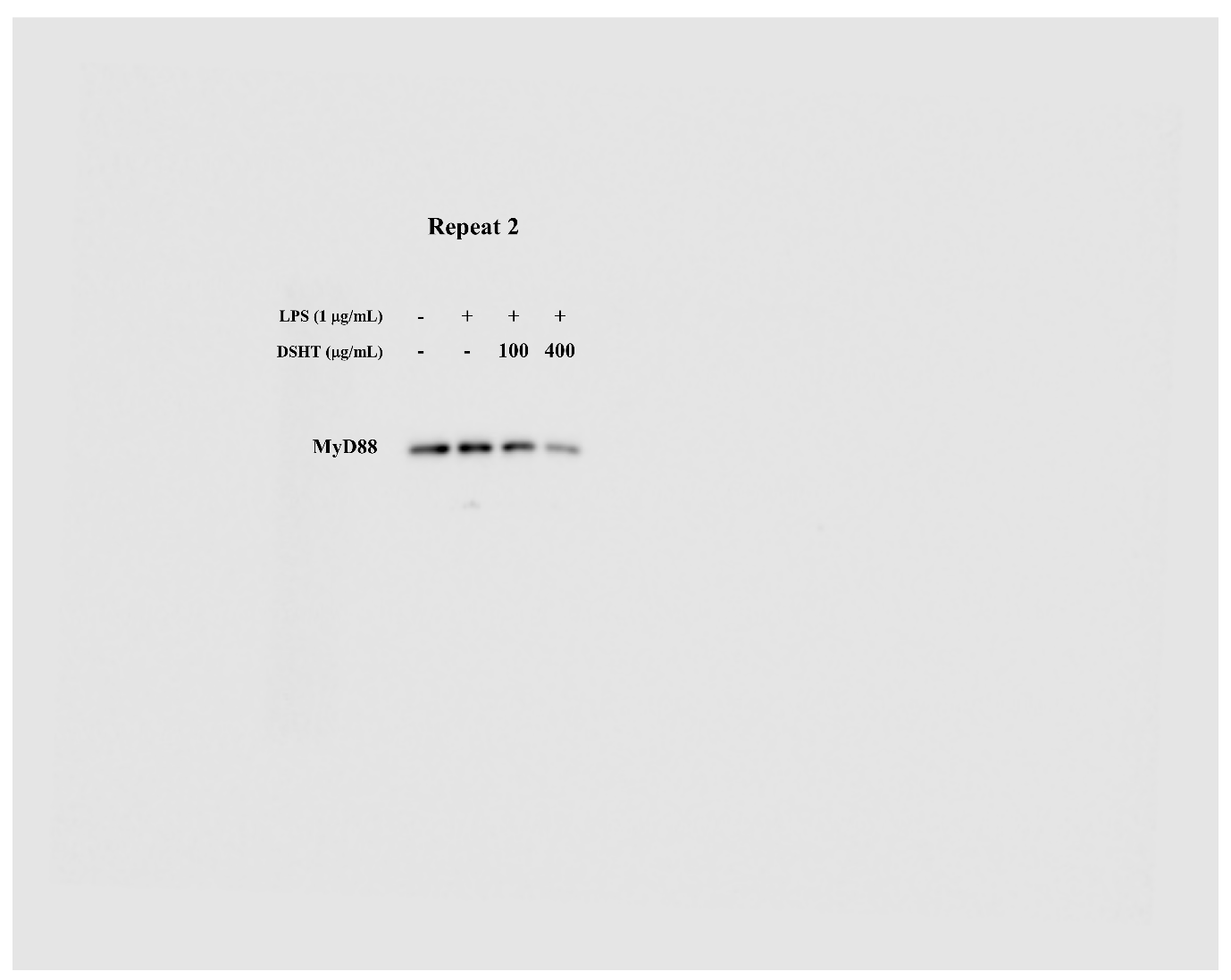


**Supplementary Fig 4**. (Continued).


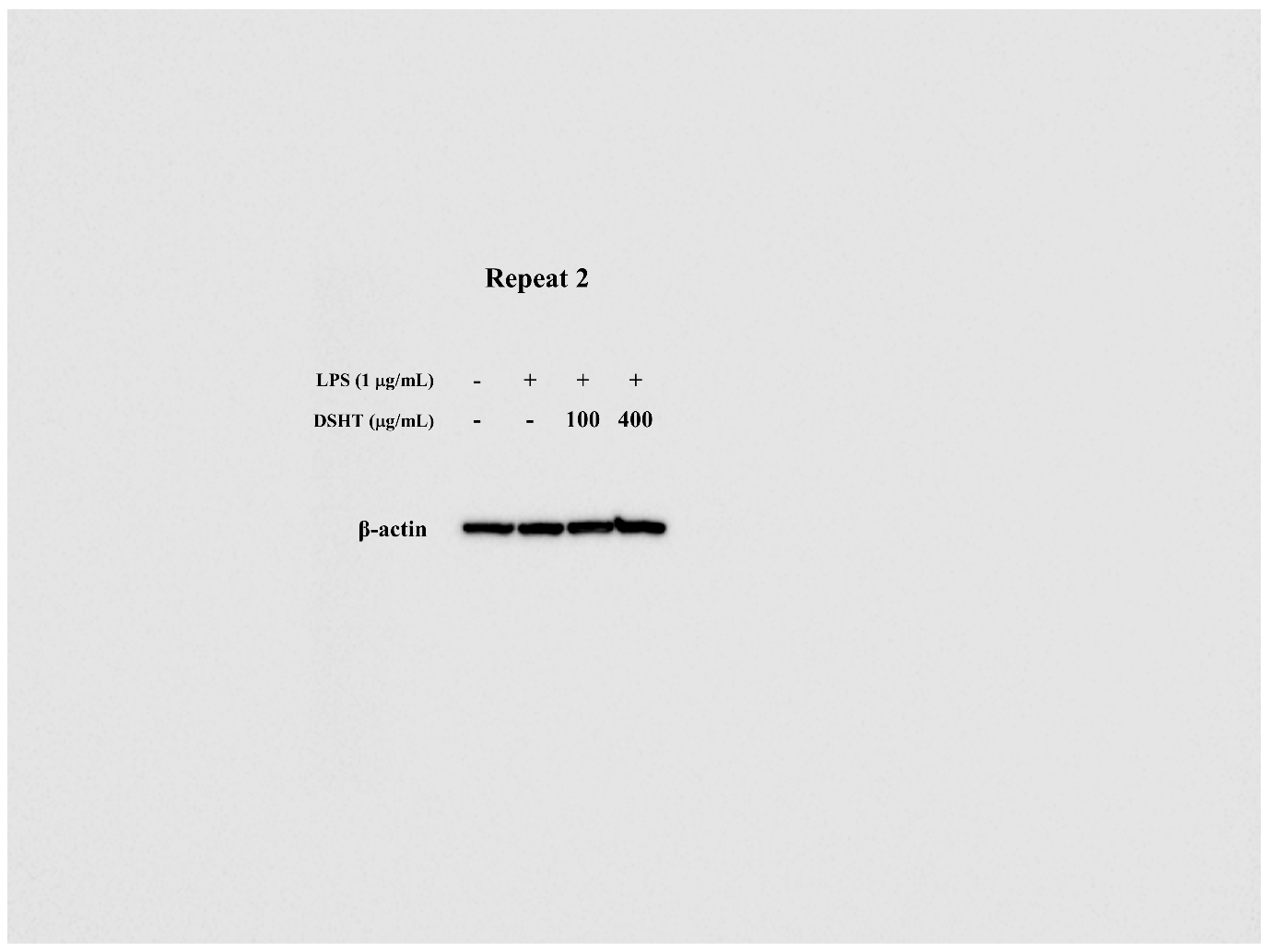


**Supplementary Fig 4**. (Continued).


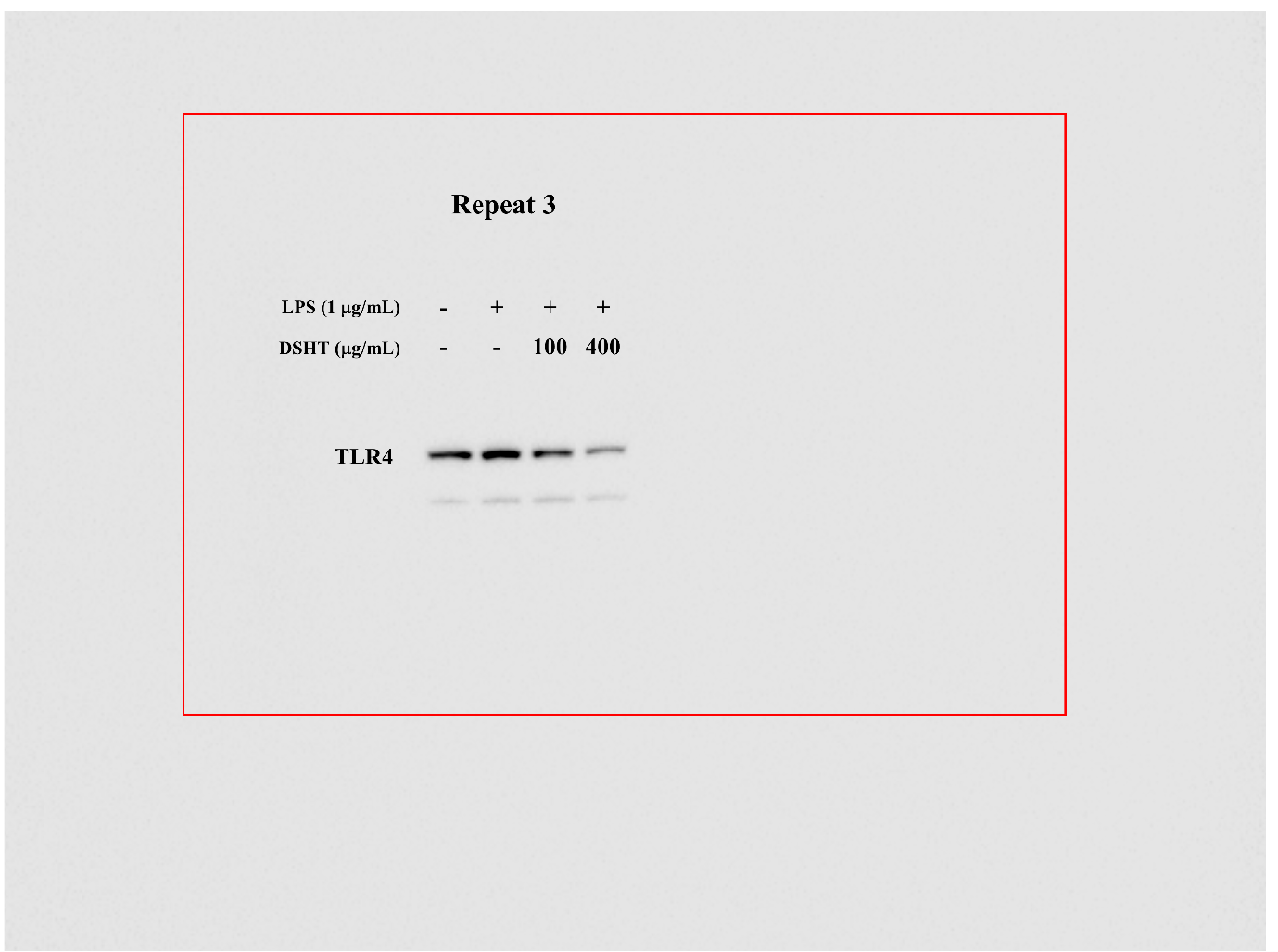


**Supplementary Fig 4**. (Continued).


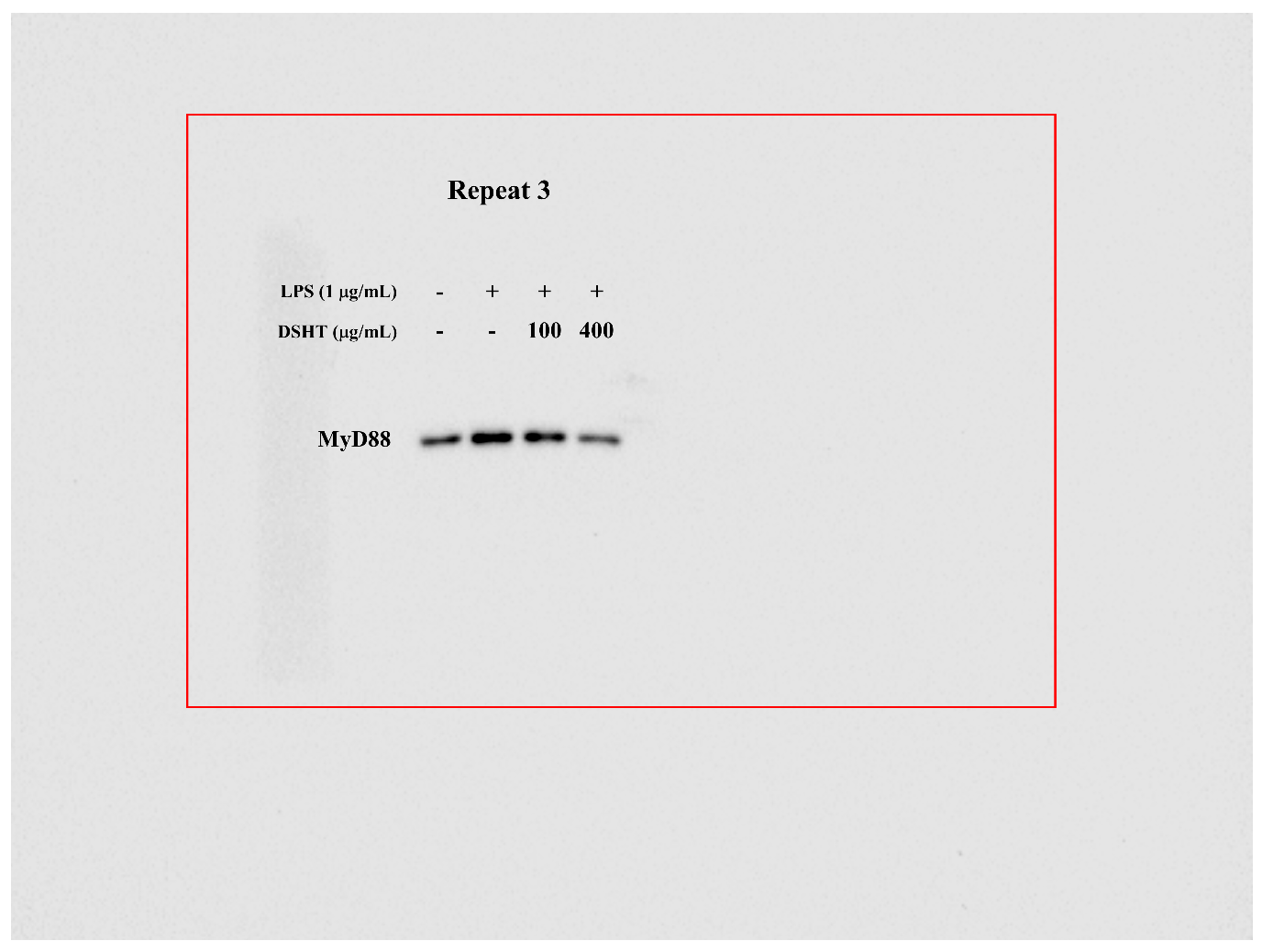


**Supplementary Fig 4**. (Continued).


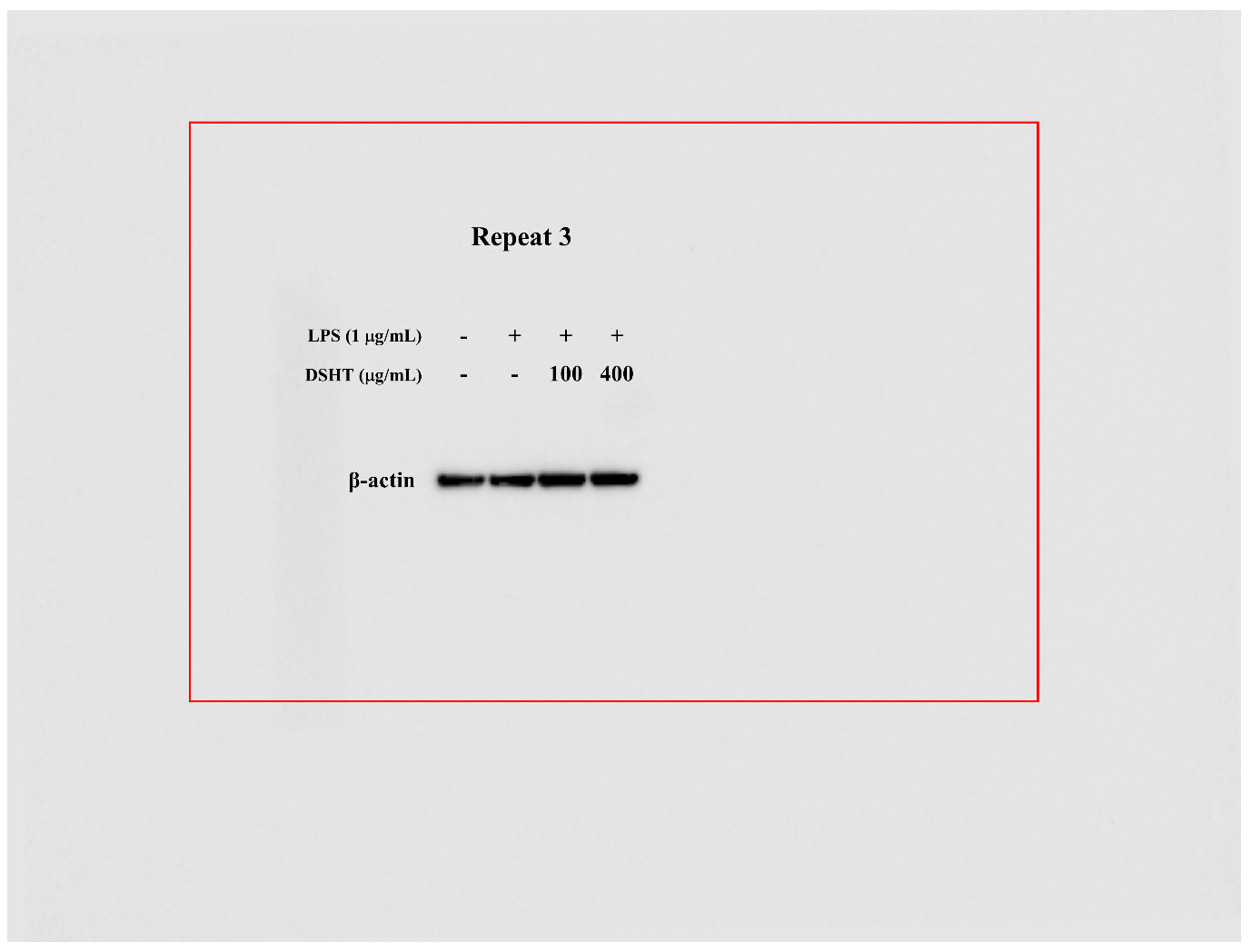


**Supplementary Fig 4**. (Continued).


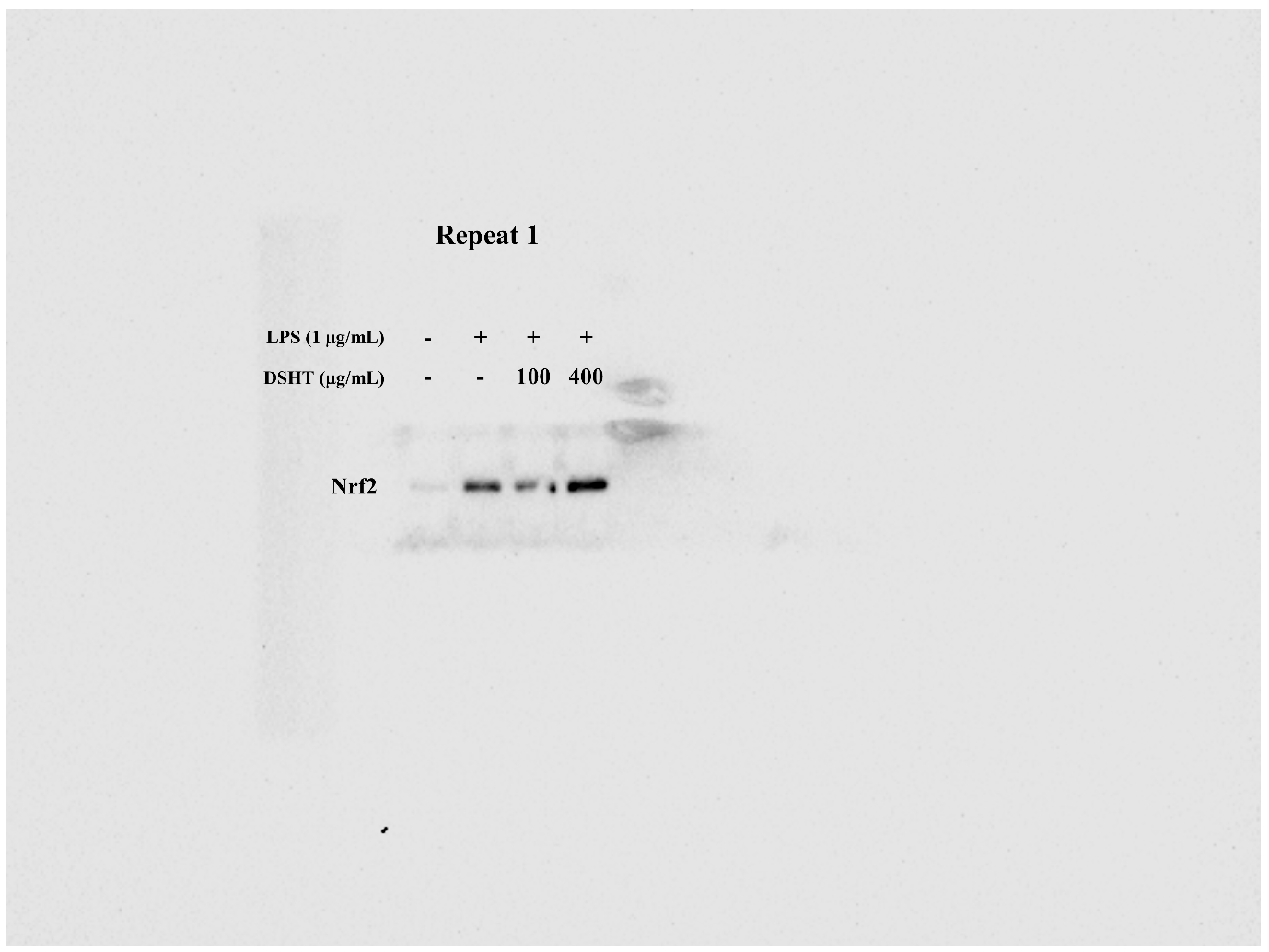


**Supplementary Fig 5**. Full-length western blots for Nrf2, HO-1 and β-actin for three repeats. Blot was cut in three before being incubated with specific antibodies. Main figures are displayed using red box. Western blot used for Fig 8.


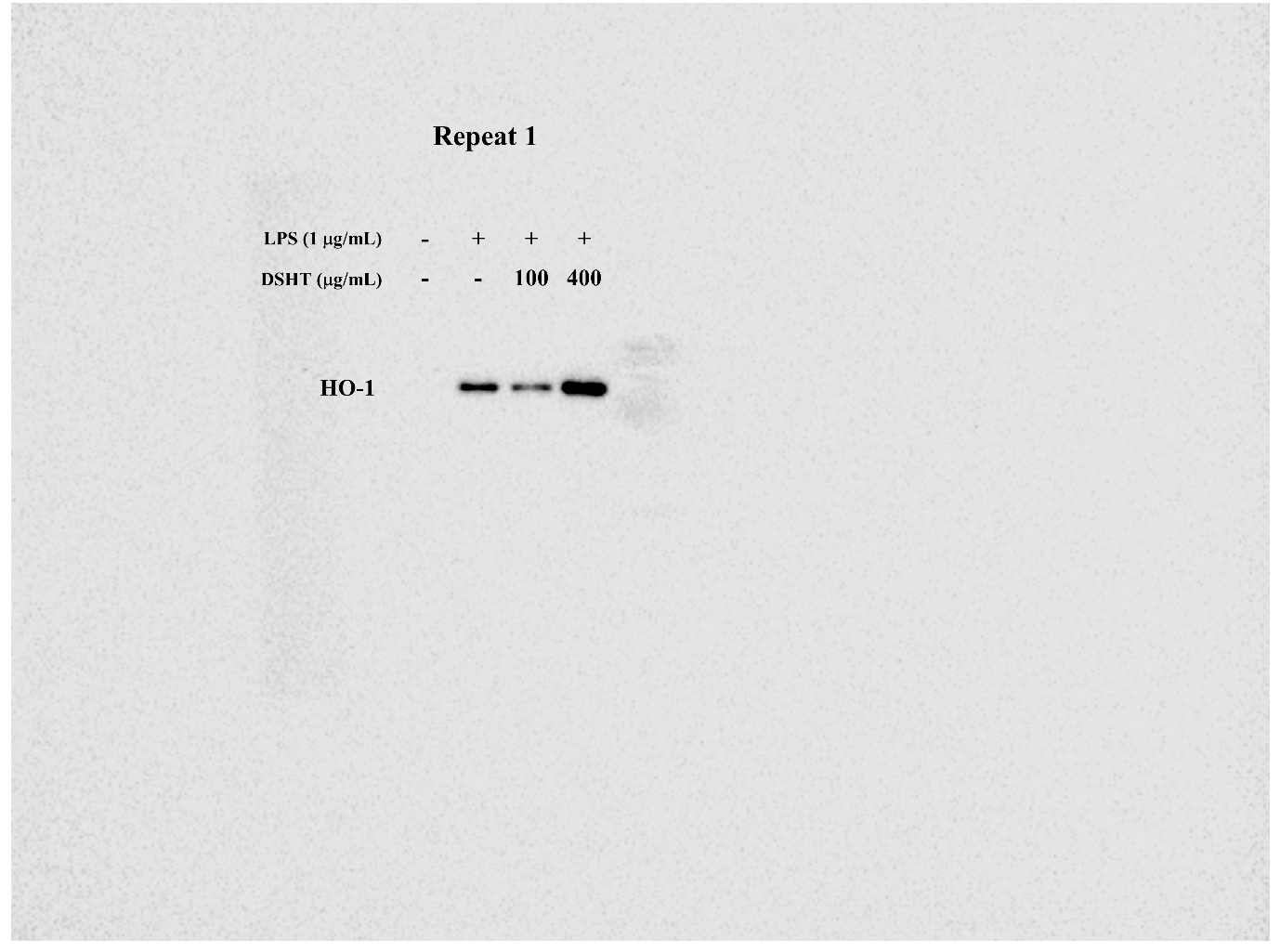


**Supplementary Fig 5**. (Continued).


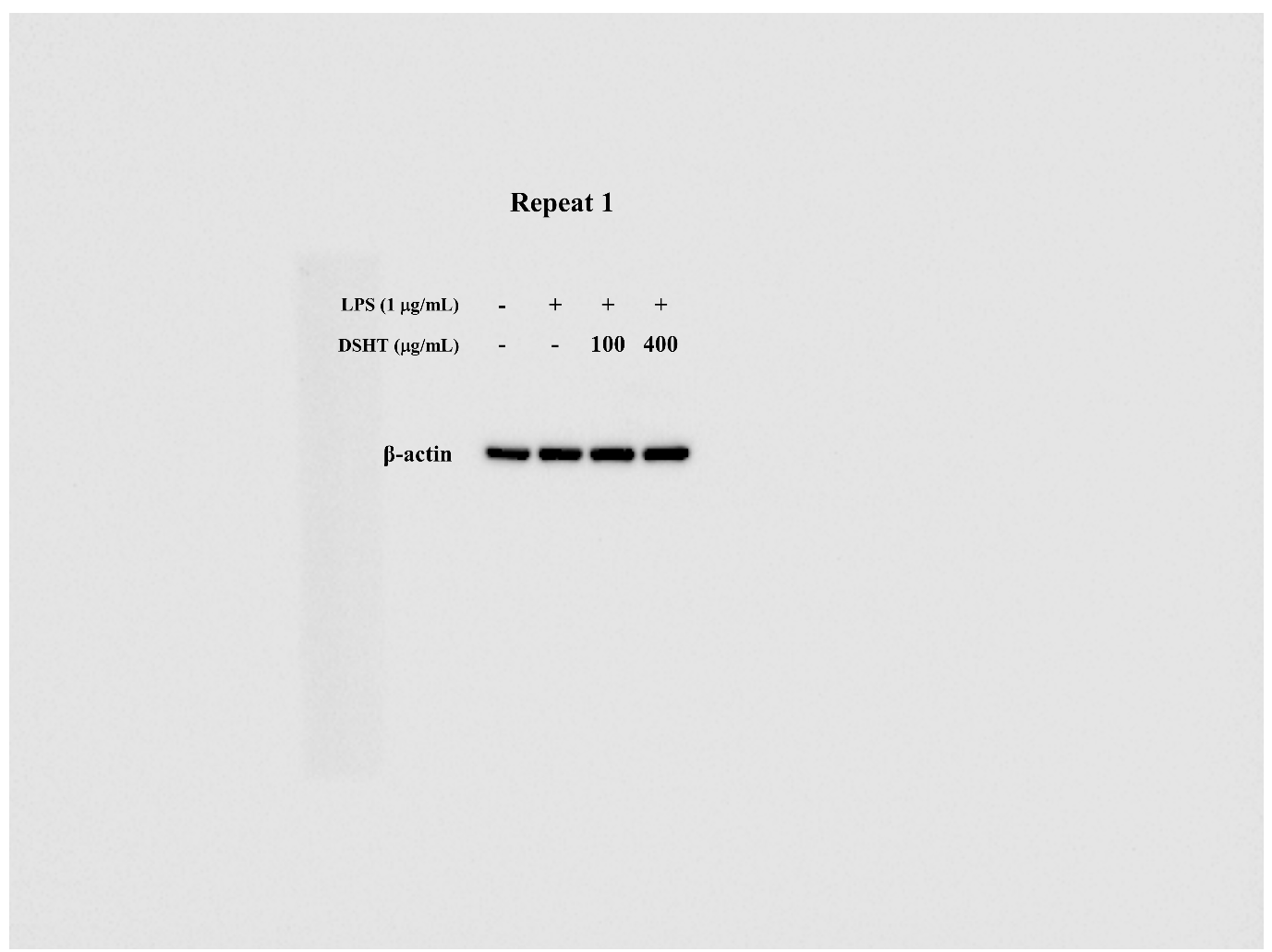


**Supplementary Fig 5**. (Continued).


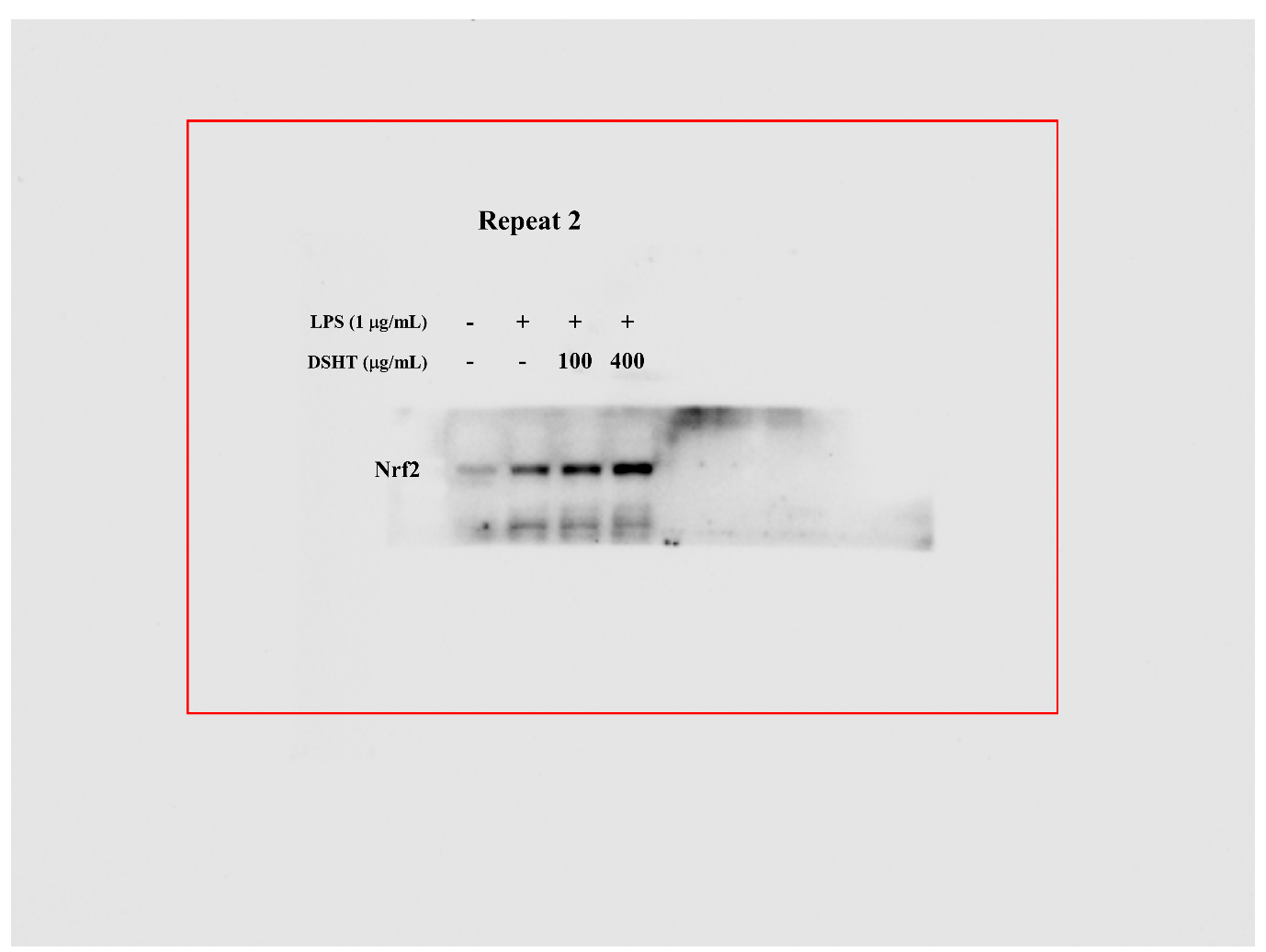


**Supplementary Fig 5**. (Continued).


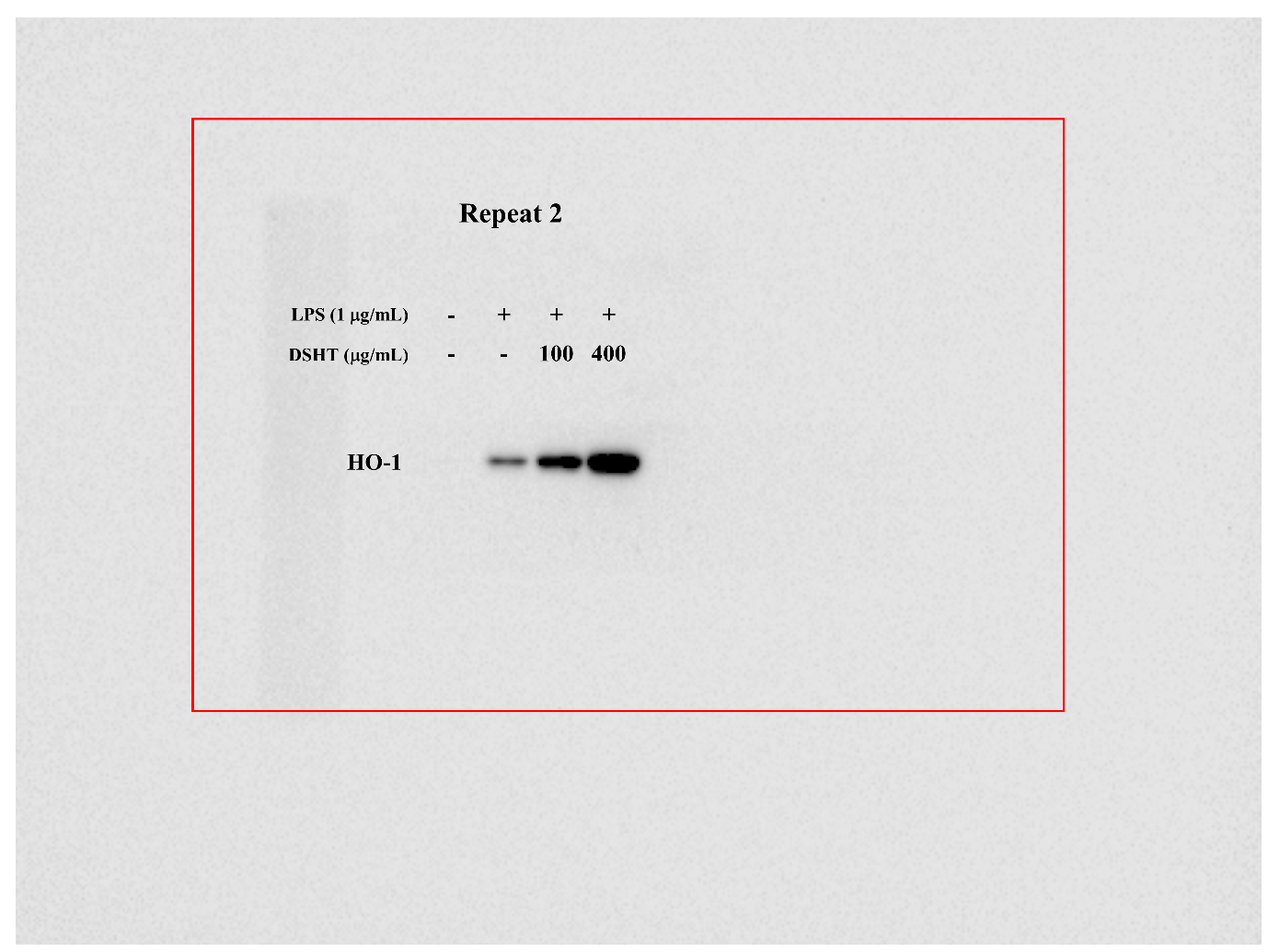


**Supplementary Fig 5**. (Continued).


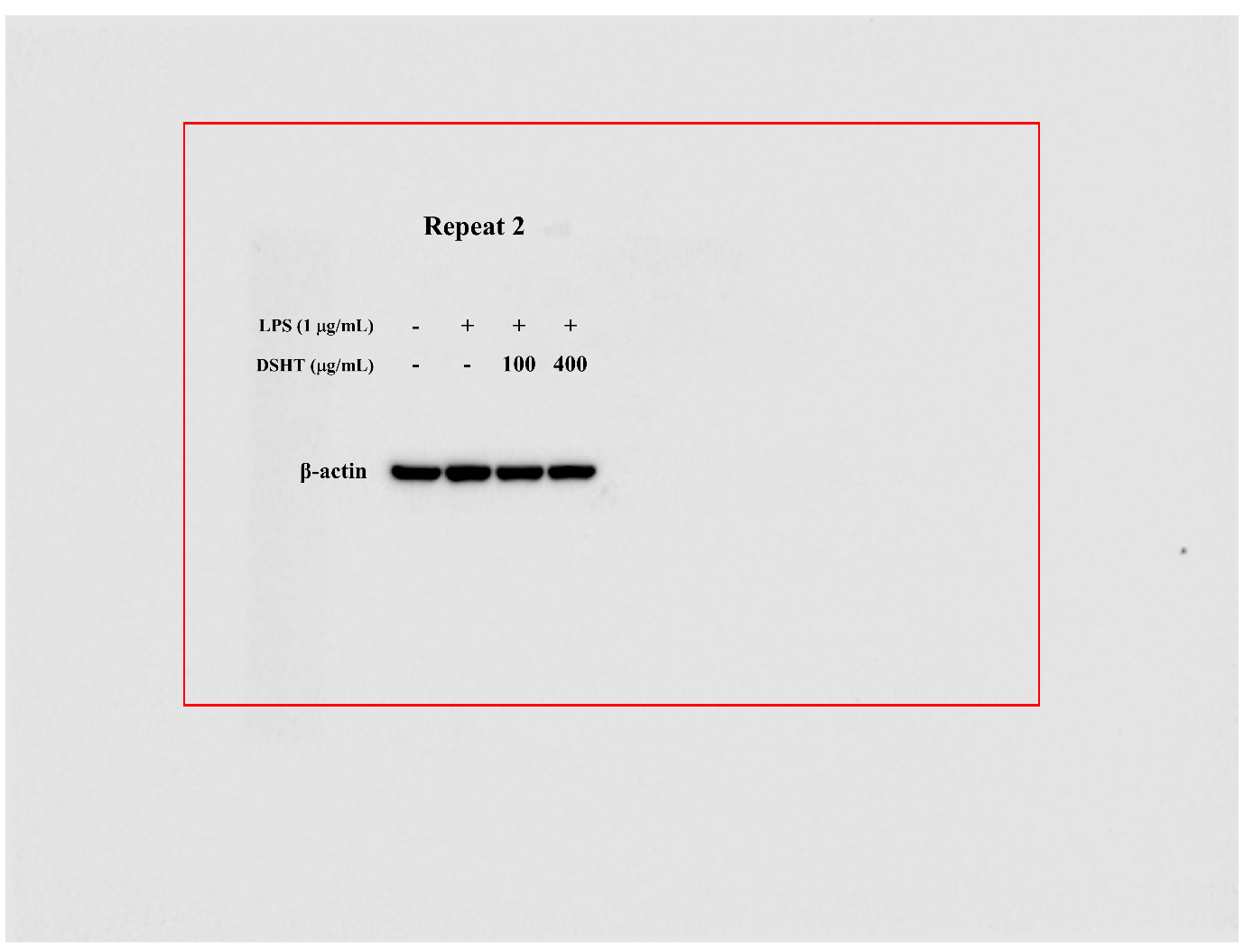


**Supplementary Fig 5**. (Continued).


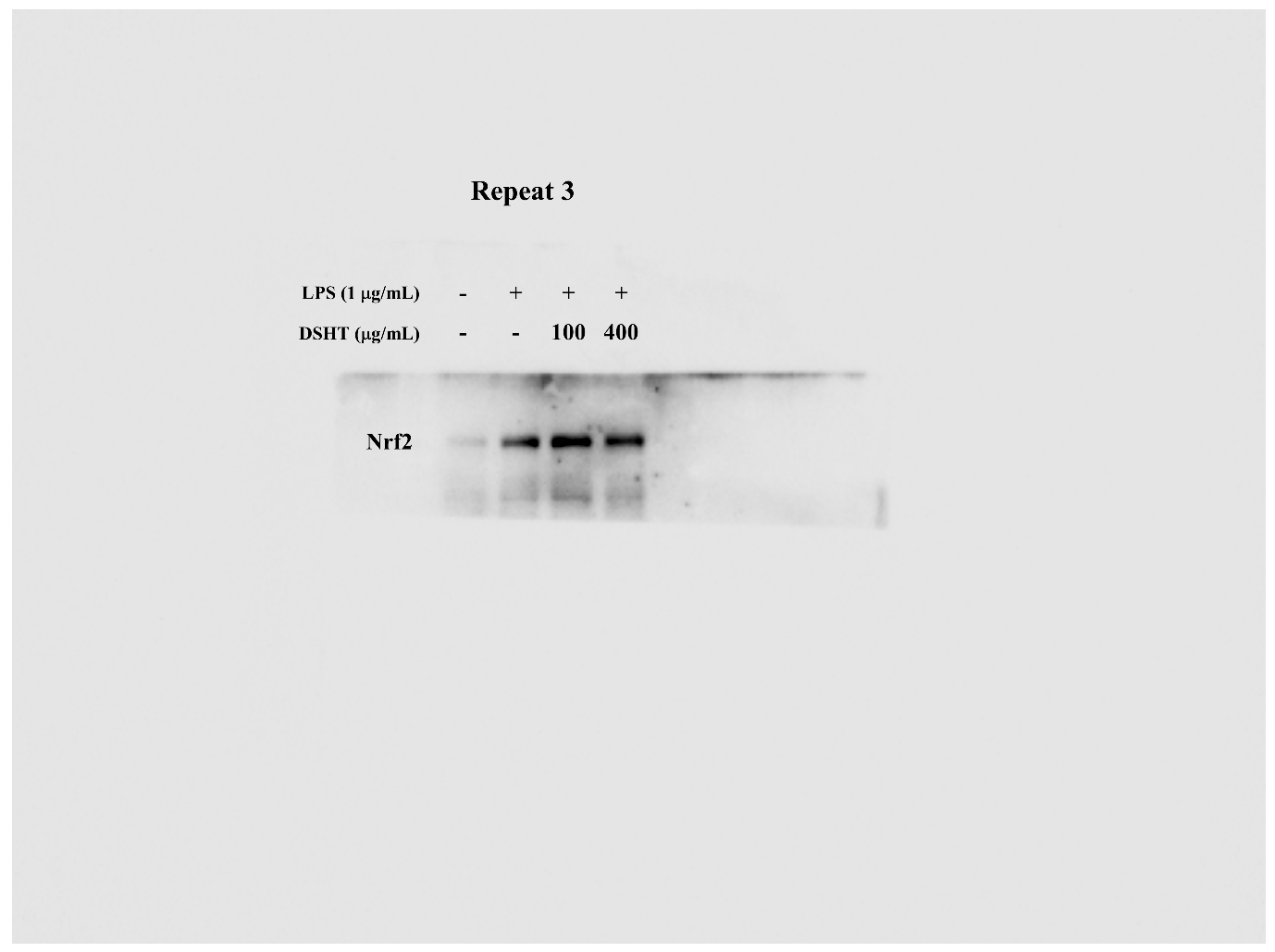


**Supplementary Fig 5**. (Continued).


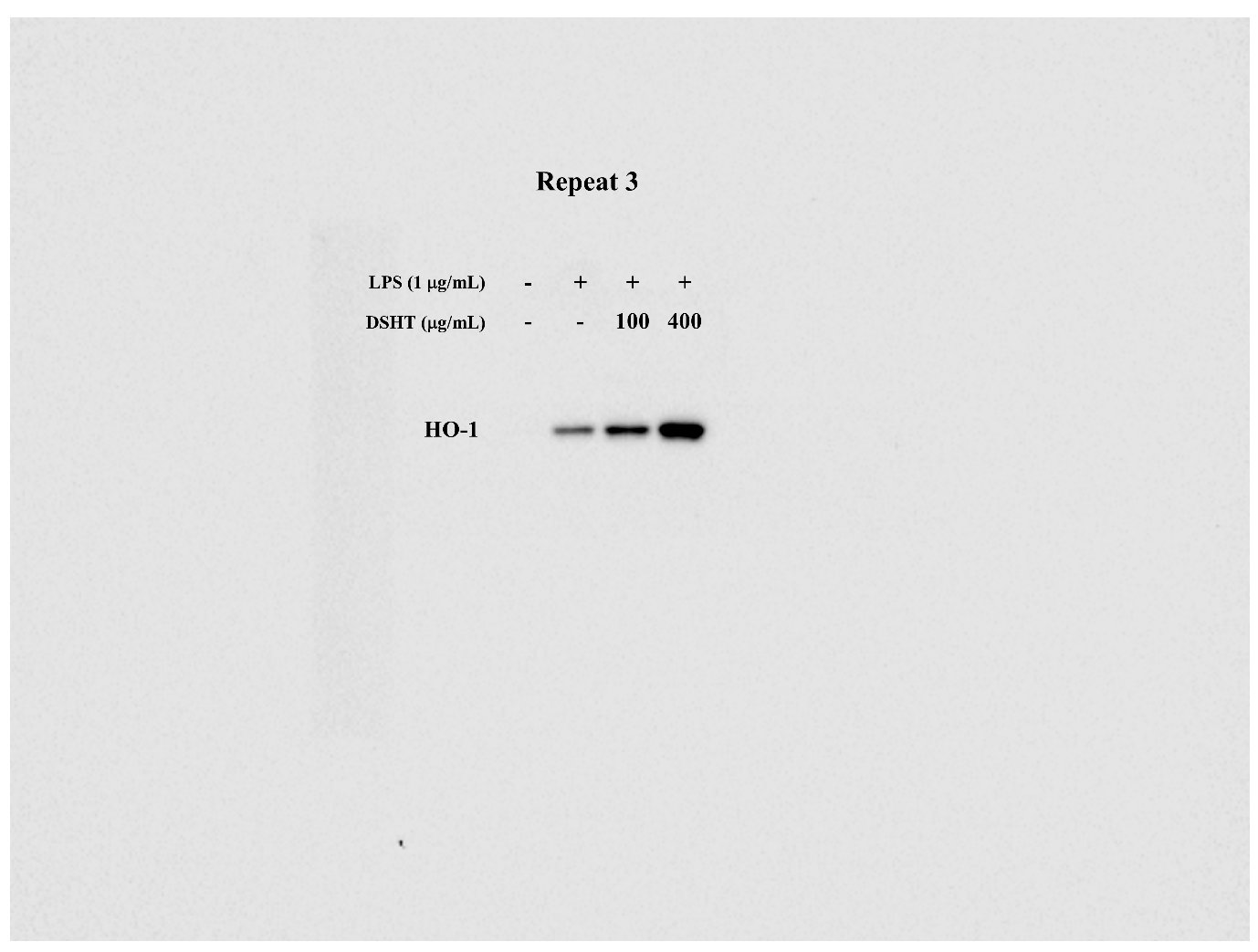


**Supplementary Fig 5**. (Continued).


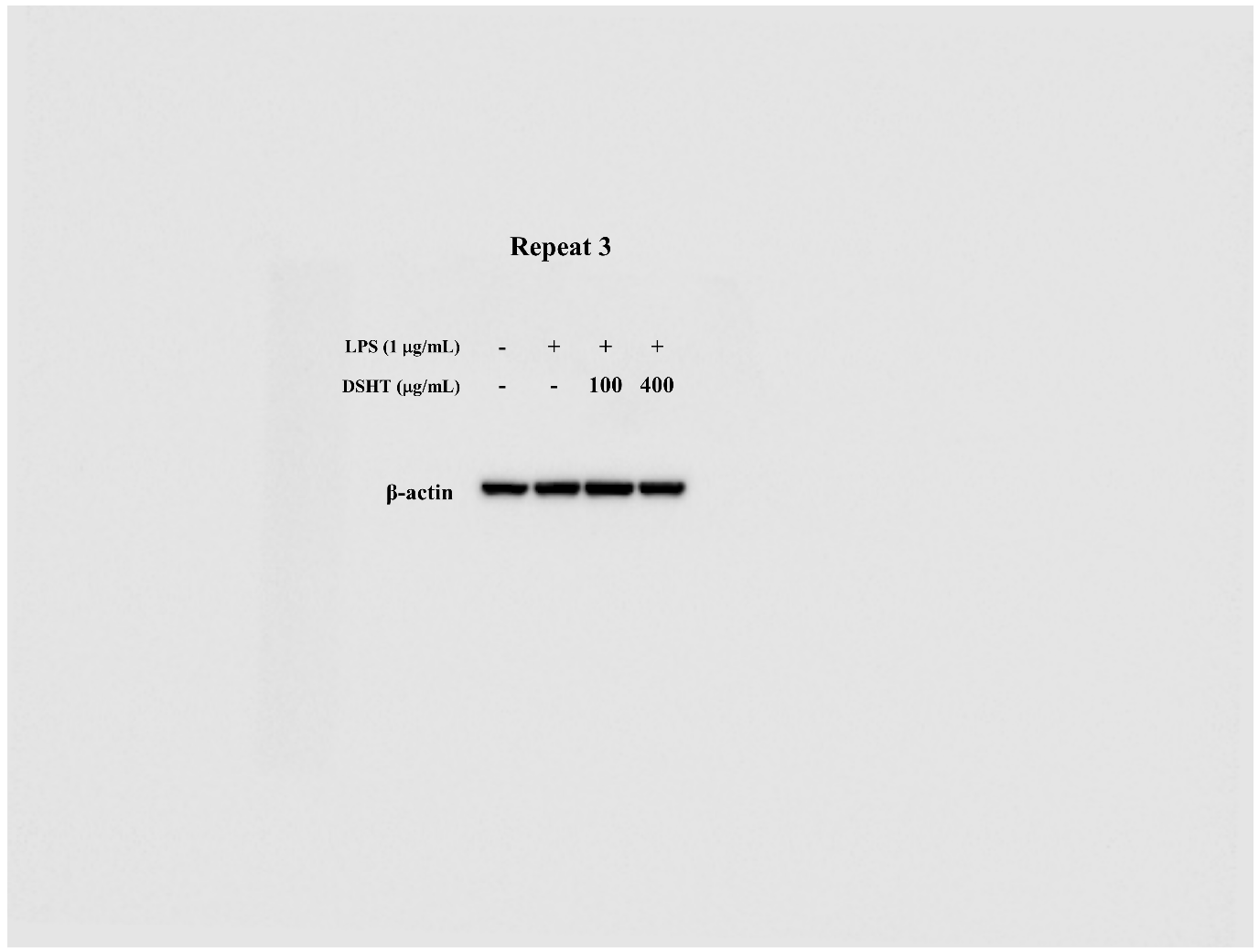


**Supplementary Fig 5**. (Continued).
